# Wood Modification by Furfuryl Alcohol Resulted in a Delayed Decomposition Response in *Rhodonia* (*Postia*) *placenta*

**DOI:** 10.1128/AEM.00338-19

**Published:** 2019-07-01

**Authors:** Inger Skrede, Monica Hongrø Solbakken, Jaqueline Hess, Carl Gunnar Fossdal, Olav Hegnar, Gry Alfredsen

**Affiliations:** a Department of Biosciences, University of Oslo, Oslo, Norway; b Department of Forest Health, Norwegian Institute of Bioeconomy Research, Ås, Norway; c Faculty of Chemistry, Biotechnology & Food Science, Norwegian University of Life Sciences, Ås, Norway; d Department of Wood Technology, Norwegian Institute of Bioeconomy Research, Ås, Norway; University of Toronto

**Keywords:** *Pinus radiata*, *Postia placenta*, furfurylation, gene regulation, radiata pine, transcriptome, wood decay, wood modification

## Abstract

The aim of this study was to investigate differential expression profiles of the brown rot fungus Rhodonia placenta (previously Postia placenta) harvested at several time points when grown on radiata pine (Pinus radiata) and radiata pine with three different levels of modification by furfuryl alcohol, an environmentally benign commercial wood protection system. The entire gene expression pattern of a decay fungus was followed in untreated and modified wood from initial to advanced stages of decay. The results support the current model of a two-step decay mechanism, with the expression of genes related to initial oxidative depolymerization, followed by an accumulation of transcripts of genes related to the hydrolysis of cell wall polysaccharides. When the wood decay process is finished, the fungus goes into starvation mode after five weeks when grown on unmodified radiata pine wood. The pattern of repression of oxidative processes and oxalic acid synthesis found in radiata pine at later stages of decay is not mirrored for the high-furfurylation treatment. The high treatment level provided a more unpredictable expression pattern throughout the incubation period. Furfurylation does not seem to directly influence the expression of core plant cell wall-hydrolyzing enzymes, as a delayed and prolonged, but similar, pattern was observed in the radiata pine and the modified experiments. This indicates that the fungus starts a common decay process in the modified wood but proceeds at a slower pace as access to the plant cell wall polysaccharides is restricted. This is further supported by the downregulation of hydrolytic enzymes for the high treatment level at the last harvest point (mass loss, 14%). Moreover, the mass loss does not increase during the last weeks. Collectively, this indicates a potential threshold for lower mass loss for the high-furfurylation treatment.

**IMPORTANCE** Fungi are important decomposers of woody biomass in natural habitats. Investigation of the mechanisms employed by decay fungi in their attempt to degrade wood is important for both the basic scientific understanding of ecology and carbon cycling in nature and for applied uses of woody materials. For wooden building materials, long service life and carbon storage are essential, but decay fungi are responsible for massive losses of wood in service. Thus, the optimization of durable wood products for the future is of major importance. In this study, we have investigated the fungal genetic response to furfurylated wood, a commercial environmentally benign wood modification approach that improves the service life of wood in outdoor applications. Our results show that there is a delayed wood decay by the fungus as a response to furfurylated wood, and new knowledge about the mechanisms behind the delay is provided.

## Abstract (precis)

Fungi are important decomposers of woody biomass in natural habitats. Investigation of the mechanisms employed by decay fungi in their attempt to degrade wood is important for both the basic scientific understanding of ecology and carbon cycling in nature and for applied uses of woody materials. For wooden building materials, long service life and carbon storage are essential, but decay fungi are responsible for massive losses of wood in service. Thus, the optimization of durable wood products for the future is of major importance. In this study, we have investigated the fungal genetic response to furfurylated wood, a commercial environmentally benign wood modification approach that improves the service life of wood in outdoor applications. Our results show that there is a delayed wood decay by the fungus as a response to furfurylated wood, and new knowledge about the mechanisms behind the delay is provided.

## INTRODUCTION

Wood as a building material has a number of attractive properties, including carbon sequestration during its service life and its aesthetic aspects as a natural material. One of the main challenges with using wood as a building material is its susceptibility to attack by wood-degrading microorganisms. Traditional wood preservatives with a biocidal mode of action (e.g., organic- or copper-based preservatives) cause concerns due to their perceived negative environmental impacts. In Europe, the Construction Products Regulation and the Biocidal Products Regulation have a major impact on the wood industry, and the number of active ingredients allowed for wood preservatives is decreasing (1). This, together with new consumer awareness and demand for more environmentally focused products (2), pushes the need for new products and wood protection approaches.

The modern wood modification approach, in contrast to more traditional wood preservatives, is to modify the wood matrix so that it can no longer act as a suitable substrate for wood-degrading organisms. Different wood modification approaches were described in detail by Hill (3). Currently, the processes underlying available wood modification products can be classified as chemical processing (acetylation, furfurylation, resin impregnation, etc.), thermo-hydroprocessing (thermal treatment), and thermo-hydromechanical processing (surface densification) (2). The different wood modification processes are at various stages of development, and the most established processes on the market include thermal treatment, acetylation, and furfurylation.

The commercial wood modification process furfurylation uses furfuryl alcohol, which is manufactured industrially by catalytic reduction of furfural obtained from agricultural waste such as sugarcane bagasse or corn cobs (4). It involves a wood impregnation step with furfuryl alcohol and catalysts, followed by a curing step where the furfuryl alcohol is polymerized within the wood cell walls (2, 4). This is a complex chemical reaction, and there is still some discussion whether the furfurylation process only bulks the wood cell wall or if it also causes chemical modifications of the native wood cell wall polymers. As support for the latter theory, it has been shown in a model lignin system that the furfural polymer formed covalent bonds with lignin (5). In addition to this uncertainty, the mechanisms utilized by decay fungi in their attempt to degrade modified wood are not well understood at the molecular level, and this hinders knowledge-based design of the modification methods.

Independently of how the furfurylation modifies the wood, the process results in improved resistance to fungal deterioration (2–4, 6, 7). The level of modification by furfural alcohol is measured by the weight percent gain (WPG) of the wood. Lande et al. (6) concluded that furfurylated wood treated to a WPG of 35% or more has sufficient resistance to brown and white rot decay fungi.

Traditionally, wood-degrading basidiomycetes have been divided into white or brown rot fungi due to the ability of white rot fungi only to degrade lignin along with holocellulose, while brown rot fungi leave the lignin behind as a brown residue. White rot fungi have a larger repertoire of known enzymes that depolymerize the components of the plant cell wall than brown rot fungi (8). It is important to note that this traditional dichotomy is polyphyletic, and it has been suggested, based on comparative studies of 33 basidiomycetes, that a continuum exists, without a clear distinction between the types of decay (8). Brown rot fungi dominate the decomposition of conifer wood in boreal forests even if only 6% of fungal wood decay species produce the classical brown rot decay (9). Moreover, brown rot causes more challenges than white rot for wood in service outdoors (10). Brown rot fungi depolymerize cellulose rapidly during incipient stages of wood colonization, resulting in considerable losses in strength even at early decay stages (11, 12). The full extent of feedback mechanisms regulating brown rot decay is not known. However, it is generally agreed that brown rot fungi utilize a nonenzymatic system that rapidly depolymerizes cell wall components in early stages of decay, prior to degradation by traditional cellulases and hemicellulases (13–18). This two-step mechanism has been further supported by gene expression and secretome patterns in the brown rot model species *Rhodonia placenta* (13). It is generally agreed that reactive oxygen species (ROS) produced via extracellular mechanisms (e.g., the Fenton reaction) are important during incipient brown rot degradation. However, there have been various mechanisms proposed to explain the reduction of Fe^3+^ to Fe^2+^ performed by brown rot during decay. It has been proposed that 2,5-dimethoxyhydroquinone (DMHQ) is the extracellular reductant of Fe^3+^, and DMHQ has been demonstrated to play a bioactive role when wood is attacked by members of three divergent fungal lineages, Boletales, Gloeophyllales, and Polyporales (14). A laccase from *R. placenta* has been implicated in oxidative attack of the wood cell wall components and has been demonstrated to oxidize 2,5-DMHQ, which in the presence of iron generates reactive oxygen species (15). One proposed model is often referred to as the chelator-mediated Fenton (CMF) system and involves the solubilization of iron from the environment by oxalic acid that the fungus secretes, followed by the reduction of iron by chelating/reducing secondary metabolites that then can react with hydrogen peroxide within the wood cell wall. It is theorized that this produces hydroxyl radicals through a Fenton-like reaction that depolymerizes lignocellulose and make these polymers accessible to hydrolytic enzymes (16). Whether the CMF functions as a pretreatment of the wood, opening the cell walls and allowing enzymes to penetrate the cell wall, or whether polysaccharide components diffuse into the cell lumen to the enzymes is currently under debate (17). However, there is agreement in that the brown rot oxidative systems and the hydrolytic enzymes cannot occupy the same space, since highly reactive oxygen species (ROS) lead to the inactivation of these enzymes. Zhang and Schilling (18) found that cellobiose appears to play a key role in the transition between the oxidative phase and the hydrolytic phase.

Rhodonia (Postia) placenta is a commonly used brown rot decay fungus in laboratory wood decay tests; strain FPRL 280 is included the European standard CEN-EN 113 (19), and strain ATCC 11538 is included in the American standard E10-16 (20). The genomes of R. placenta strain MAD 698-R and its monokaryotic strain MAD698-R-SB12 have been sequenced by the Department of Energy-Joint Genome Institute (JGI) (21, 22). The species has frequently been used as a model fungus for gene expression studies of untreated wood (13, 18, 22–24) and modified wood (25–31), including furfurylated wood (27, 30). These experiments have shown that several hemicellulases but few potential cellulases were produced when the fungus was grown on ball-milled aspen or glucose as the substrate (23). The expression patterns for oxidoreductase-encoding genes support an extracellular oxidative decay system. Furthermore, Skyba and colleagues (32) demonstrated that the gene expression profile of *R. placenta* (and Phanerochaete chrysosporium) was influenced by wood substrate composition (three Populus trichocarpa genotypes) and the duration of incubation. For early stages of modified wood, a possible shift toward increased expression of genes related to oxidative metabolism and concomitant reduction of several gene products related to the breakdown of holocellulose in furfurylated wood compared to unmodified wood have been suggested (27).

In this study, we investigated the differential expression profiles of the brown rot fungus *Rhodonia placenta* harvested at several time points when grown on *Pinus radiata* (radiata pine) with three different levels of furfurylation. For comparison, we also investigated the gene expression during decay of unmodified radiata pine. Radiata pine is native to central and southern coastal California but is also widely planted throughout the Southern Hemisphere. Radiata pine was selected in this study because it is the most frequently used wood species by the largest commercial producer of furfurylated and acetylated wood (wood modifications). They use radiata pine because of the low heartwood proportion and because it is easy to modify (i.e., has good penetration).

The overall aim is to understand the mechanisms utilized by brown rot decay fungi in their attempt to degrade modified and unmodified wood. This is important for further optimization of future modified wood products and for an expanded understanding of the fungal decay process in general.

## RESULTS

In the following experiments, the radiata pine wood was modified at three different levels. The three modification levels had mean weight percent gain (WPG) levels of 3.8% ± 0.7%, 24.0% ± 3.5%, and 36.6% ± 5.0%. For simplicity, the treatments are named WPG4, WPG24, and WPG37, respectively. The experiment on the unmodified radiata pine is named “unmodified wood.”

### Mass loss calculations.

**FIG 1:**
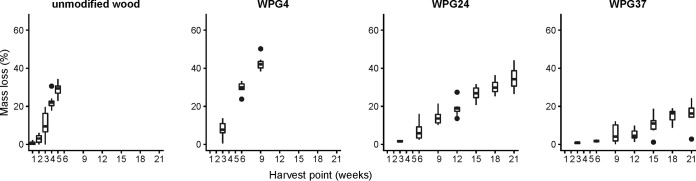
Boxplots of the mass loss of all experiments of *Rhodonia placenta* grown on *Pinus radiata* and different levels of modification by furfurylated radiata pine. The weight of the treatment is included the measurements. Left, *R. placenta* grown on unmodified radiata pine. Wood was harvested at five different harvest points. Second from left, *R. placenta* grown on furfurylated radiata pine, with a weight percent gain (WPG) of 4%. Wood was harvested at three different harvest points. For the third panel from left and the rightmost panel, the wood was harvested at seven harvest points for *R. placenta* grown on furfurylated radiata pine, at WPG 24% (third from left) and WPG 37% (right).

Five weeks after inoculation of *R. placenta* strain FPRL 280 on the unmodified wood blocks, they had a mean mass plus treatment loss of 28.8% ± 4.0%, while the modified WPG37 only had a mean mass plus treatment loss of 13.5% ± 3.3% after 21 weeks (Fig. 1). Mass loss is within the wood protection literature mostly referred to as the mass loss of the entire wood plus treatment system, i.e., the dry weight of the treated wood before fungal inoculation compared to the dry weight after fungal decay. Another way to measure mass loss is to assume that only the wood is decayed and eliminate the WPG of the treatment from the mass loss calculation. The two methods resulted in the same general trends, but the exclusion of WPG results in slightly higher mass losses, as expected (see Fig. S1 in the supplemental material). The differences between the two mass loss calculation approaches for the three furfurylation levels at the last harvesting points were (wood plus treatments versus only wood) WPG4 week 9, 41.7% versus 42.5%; WPG24 week 21, 29.0% versus 34.6%; and WPG37 week 21, 13.5% versus 16.0%. We use the wood plus treatment results as presented in Fig. 1 as our mass loss detection system.

### Differential gene expression.

In order to investigate the genetic basis of the different behavior of *R. placenta* growing on different levels of furfurylated wood, we sequenced the transcriptomes of a selection of time series using Illumina NextSeq sequencing. The sequenced time points for each treatment were sampled according to the following setup: unmodified wood, weeks 1 to 5; WPG4, weeks 3, 6, and 9; WPG24, weeks 3, 6, 9, 12, 15, and 18; and WPG37, weeks 3, 6, 9, 12, 15, and 18.

Even if the genome and transcriptome of the American *R. placenta* strain MAD 698-R have been sequenced, this could not be used for mapping purposes in this study. The genome-sequenced American strain MAD 698-R and the European strain FPRL 280 used in this study are significantly different, with a mapping success of only 40 to 60% when the reads were mapped to the genome of MAD 698-R (results not shown). We therefore produced Illumina HiSeq paired-end data to be used for a transcriptome assembly. The resulting assembly had 56,520 contigs and a total of 114,539 transcripts when including all isoforms (Table S1). The transcriptome covered 99.3% of the conserved BUSCO fungal genes (Table S2). In the transcriptome, 9,355 contigs had an annotation from the UniProt database in BLASTX, and 18,917 contigs were given a PFAM annotation. In all further analyses, the sequence data were mapped to this annotated transcriptome assembly. The mapping success of sequence data to this transcriptome was more than 90% for all libraries.

The gene expression data demonstrate consistent results for each treatment with a gradient clustering of replicate samples according to treatment, with few outliers (Fig. S3). There is some biological variation between replicates as expected from natural variation in wood and the variation in modification of the wood blocks. The unmodified wood and WPG37 experiments display a wider range of biological variation than do WPG24 and WPG4.

### Differential gene expression and functional enrichment across treatments.

To obtain an overall impression of the entire data set, we ran a multifactorial differential expression (DE) analysis, factoring in time and treatment. Thus, all harvest time points were included for all treatments, and we extracted contrasts describing the different modified treatments versus the unmodified wood experiment, i.e., WPG4 versus unmodified wood, WPG24 versus unmodified wood, and WPG37 versus unmodified wood. (Table 1). Functional enrichment analyses of the resulting gene lists were inferred using the annotated PFAM domains and GO terms from the annotated transcriptome. Compared to the unmodified wood, the WPG4 showed upregulation of zinc-binding dehydrogenase domains (PF00107.21) and two GO terms related to zinc ion binding and oxidoreductase activity. The same enrichment was also found to be upregulated in WPG24 and WPG37 compared to unmodified wood. In addition, WPG24 and WPG37 treatments showed upregulation of more oxidoreductase domains, with most in WPG37. No PFAM domains or GO terms were found to be downregulated in WPG4 or WPG24. However, WPG37 showed a strong downregulation of eight PFAM domains and seven GO terms with functions related to protein and peptide degradation, the ubiquitin-proteasome pathway, and the Ras gene family compared to unmodified wood (Table 1), e.g., two domains related to the proteasome (PF10584.4 and PF00227.21) and Ras (PF08477.8 and PF00071.17). These terms were not found among the other comparisons.

**TABLE 1:** Functional enrichment analyses of GO terms and PFAM domains of significant differentially expressed genes between treatments along the time series of *Rhodonia placenta* growing on unmodified radiata pine and three different levels of modification with furfuryl alcohol

Treatment comparison^*a*^	No. of DE genes	GO term(s) and/or PFAM domain(s)	Frequency (no. of genes/total no. [%])	Adjusted *P* value	Description^*b*^	Comment^*c*^
DOWN WPG4–UP unmodified	38	No enrichment				
UP WPG4–DOWN unmodified	32	GO:0008270	8/562 (1.42)	4.699815e−03	MF	Zinc ion binding
		GO:0016491	8/736 (1.09)	3.389974e−02	MF	Oxidoreductase activity
		PF00107.21	4/76 (5.26)	1.536767e−02		Zinc-binding dehydrogenase
UP WPG24–DOWN unmodified	228	GO:0016491	40/736 (5.43)	9.508001e−13	MF	Oxidoreductase activity
		GO:0055114	46/1,163 (3.96)	5.487584e−10	BP	Oxidation-reduction process
		PF00107.21	12/76 (15.79)	4.007640e−07		Zinc-binding dehydrogenase
		PF13602.1	7/30 (23.33)	1.059814e−04		Zinc-binding dehydrogenase
		PF00106.20	13/198 (6.57)	3.016412e−03		Short-chain dehydrogenase
		PF08659.5	11/142 (7.75)	3.531839e−03		KR domain
		PF13561.1	9/105 (8.57)	1.170520e−02		Zinc-binding dehydrogenase
DOWN WPG24–UP unmodified	241	No enrichment				
UP WPG37–DOWN unmodified	907	GO:0016491	88/736 (11.96)	6.355618e−19	MF	Oxidoreductase activity
		GO:0055114	108/1,163 (9.29)	2.403803e−15	BP	Oxidation-reduction process
		GO:0016705	38/326 (11.66)	1.483503e−06	MF	Oxidoreductase activity
		GO:0055085	46/490 (9.39)	2.946082e−05	BP	Transmembrane transport
		GO:0005506	38/367 (10.35)	3.882514e−05	MF	Iron ion binding
		GO:0020037	40/419 (9.55)	1.559430e−04	MF	Heme binding
		GO:0008152	49/664 (7.38)	1.270725e−02	BP	Metabolic process
		GO:0010181	10/54 (18.52)	3.305432e−02	MF	FMN binding
		PF08659.5	27/142 (19.01)	7.135697e−08		KR domain
		PF00106.20	30/198 (15.15)	2.054525e−06		Short-chain dehydrogenase
		PF00107.21	17/76 (22.37)	1.773561e−05		Zinc-binding dehydrogenase
		PF07690.11	29/222 (13.06)	1.097127e−04		Major facilitator family
		PF00067.17	35/309 (11.33)	2.032395e−04		Cytochrome P450
		PF13417.1	9/24 (37.50)	3.098329e−04		Glutathione *S*-transferase
		PF13561.1	18/105 (17.14)	5.010722e−04		Zinc-binding dehydrogenase
		PF00724.15	8/24 (33.33)	3.857038e−03		NADH:flavin oxidoreductase
		PF13602.1	8/30 (26.67)	2.424490e−02		Zinc-binding dehydrogenase
DOWN WPG37–UP unmodified	1,125	GO:0006511	18/86 (20.93)	1.690517e−03	BP	Ubiquitin-dependent protein catabolic process
		GO:0005839	9/23 (39.13)	2.523168e−03	CC	Proteasome core complex
		GO:0051603	9/23 (39.13)	2.523168e−03	BP	Proteolysis involved in cellular protein catabolic process
		GO:0019773	6/10 (60.00)	5.541878e−03	CC	Proteasome core complex, alpha-subunit complex
		GO:0004175	6/11 (54.55)	1.158001e−02	MF	Endopeptidase activity
		GO:0004298	9/28 (32.14)	1.630679e−02	MF	Threonine-type endopeptidase activity
		GO:0033178	5/8 (62.50)	2.652523e−02	CC	Proton-transporting two-sector ATPase complex, catalytic domain
		PF10584.4	6/8 (75.00)	1.571873e−03		Proteasome A_N
		PF00227.21	9/21 (42.86)	2.650923e−03		Proteasome
		PF08477.8	16/68 (23.53)	5.702283e−03		Ras of complex, Roc domain of DAP-kinase
		PF00009.22	13/49 (26.53)	9.992408e−03		GTP-binding elongation factor family, EF-Tu/EF-1A subfamily
		PF00025.16	16/74 (21.62)	1.783714e−02		ADP-ribosylation factor
		PF00071.17	15/71 (21.13)	4.220867e−02		Ras subfamily
		PF00928.16	5/8 (62.50)	4.438262e−02		Adaptor complexes medium subunit domain
		PF01399.22	7/17 (41.18)	4.515427e−02		PCI domain

a Treatments are weight percent gain (WPG) of wood of 4, 24, and 37%. Unmodified indicates unmodified wood. UP, upregulated; DOWN, downregulated.

b Description indicates gene ontology variables. MF, molecular function; BP, biological process; CC, cellular component.

c FMN, flavin mononucleotide; DAP, death-associated protein.

**FIG 2:**
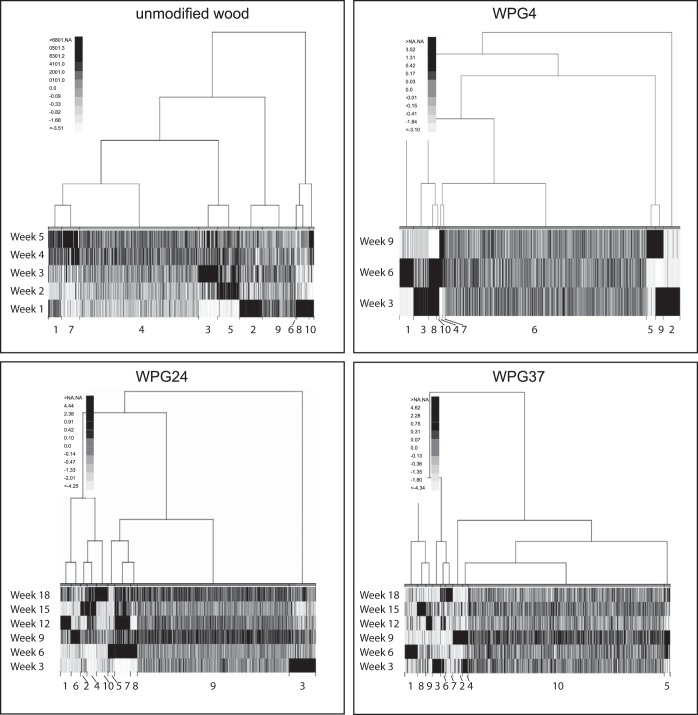
All genes were clustered into 10 groups according to their similarity in expression patterns based on read counts from RNA-seq data. Each treatment was analyzed separately. The figure visualizes the relationship among these gene clusters (tree structure) and their expression pattern related to harvest point (week). Darker color indicates higher expression.

As an alternative analysis method to the multifactorial DE analyses mentioned above, we also clustered the transcripts with similar expression profiles. The read counts were grouped into 10 clusters (K) for each treatment, and samples from unmodified wood, WPG4, WPG24, and WPG37 were analyzed separately (Fig. 2). For these clusters, we did functional enrichment with PFAM and GO terms (Table S3) and also investigated the placement of transcripts including known genes related to plant cell wall decay in these clusters (Table S4).

For most of the clusters, an observed pattern of higher expression in one or a few of the harvest points was found (Fig. 2). For the unmodified wood, two clusters were directly linked to wood decay and carbohydrate-active enzymes (Table S3). One of these clusters (unmodified wood-K2) was related to early depolymerization of hemicellulose and pectin (enriched for glycoside hydrolase 28 [GH28] and GH43 domains and containing the transcripts predicted to encode OxaD, Man5a, and CE16b; see Table 2 for information about the abbreviated gene names) and highly expressed in week 1, while the other cluster (unmodified wood-K5) was related to later stages of cellulose depolymerization (enriched for GH3 and hydrolase activity, and containing the transcripts predicted to encode Cel5b, Cel2, βGlu, Xyl10a, and βXyl; Table 2) and was highly expressed in week 2. Similar enrichments were found for two clusters of WPG4 (WPG4-K2, which is highly expressed early, containing the transcripts predicted to encode Man5a, CE16a, and Gal28a; and WPG4-K5, which was expressed late in contrast to unmodified wood and containing the transcripts predicted to encode OxaD and Xyl10a); however, no clusters can be directly compared across treatments. The following two clusters in WPG24 also have enrichment for GO terms related to hydrolase activity: WPG24-K3, which is highly induced in week 3 and contains the transcripts predicted to encode Gal28a and CE16b; and WPG24-K9, which is a large group with no specific induction pattern across harvesting points, where all the other specific transcripts we investigated were placed. The same pattern was demonstrated for WPG37, where all specific transcripts, except the transcripts predicted to encode CE16b, were placed in a large group (WPG37-K10) with no specific induction time. WPG37-10 also had enrichment for GO terms such as protein binding, oxidation-reduction processes, and catalytic activity (see Table S3 for more details). Functional enrichment was only found for one other cluster of WPG37 (WPG37-K1). This cluster was enriched for functions related to salt and water stress and was highly expressed in the second harvest point, week 6. One cluster with one or a few of the same domains as in cluster WPG37-K1 could be found for all the other treatments (unmodified wood-K1, WPG4-K6, and WPG24-K3). For the WPG24 treatment, this was induced in the first harvest point, thus earlier than for WPG37. In unmodified wood-K1 and WPG4-K6, there were no clear patterns of induction time for clusters with these stress domains (Fig. 2 and Table S3).

**TABLE 2:** Specific genes with functions related to plant cell wall decay investigated specifically in this study^*a*^

Gene by function (abbreviation)	Transcriptome ID	JGI protein ID	Function
Oxalate synthesis and oxalate decomposition			
Glyoxylate dehydrogenase (GlyD)	TRINITY_DN33196_c1_g1	121561	Involved in oxalic acid synthesis
Oxaloacetate acetylhydrolase (OahA)	TRINITY_DN26529_c1_g1	112832	Involved in oxalic acid synthesis
Oxalate decarboxylase (OxaD)	TRINITY_DN21938_c3_g2	43912	Involved in oxalate decomposition
Redox enzymes			
AA3 GMC oxidoreductase (AOx1)	TRINITY_DN18773_c0_g1	44331	Involved in oxidative depolymerization; likely source of H_2_O_2_
AA3 GMC oxidoreductases (AOx2)	TRINITY_DN20417_c3_g1	129158	Involved in oxidative depolymerization; likely source of H_2_O_2_
AA3 GMC oxidoreductase (AOx3)	TRINITY_DN28649_c4_g1	118723	Involved in oxidative depolymerization; likely source of H_2_O_2_
AA3_3 alcohol oxidase (AOx4)	TRINITY_DN21062_c1_g1	55972	Involved in oxidative depolymerization; likely source of H_2_O_2_
AA5 copper radical oxidase (Cro1)	TRINITY_DN21070_c1_g1	56703	Involved in oxidative depolymerization; likely source of H_2_O_2_
AA5 copper radical oxidase (Cro2)	TRINITY_DN9270_c1_g1	104114	Involved in oxidative depolymerization; likely source of H_2_O_2_
AA6 benzoquinone reductases (BqR)	TRINITY_DN21924_c2_g1	124517	Involved in oxidative depolymerization; possibly involved in reduction/regeneration of chelator/reductants
Cellulose degradation			
GH5 endoglucanase (Cel5a)	TRINITY_DN26393_c3_g1_i1	115648	Major endocellulase
GH5 endoglucanase (Cel5b)	TRINITY_DN21725_c8_g1	103675	Major endocellulase
GH12 glucoside hydrolase (XyGEg)	TRINITY_DN33048_c6_g2	121191	Endoglucanase active on cellulose or xyloglucan
AA9 lytic polysaccharide monooxygenase (LPMO)	TRINITY_DN16131_c0_g1	126811	Polysaccharide depolymerization via oxidative cleavage of glycosidic bonds
GH3 beta-glucosidase (βGlu)	TRINITY_DN21749_c2_g1	128500	Hydrolyses cellobiose, releasing glucose
Hemicellulose and pectin degradation			
Endomannanase (Man5a)	TRINITY_DN30802_c4_g1	121831	Involved in glucomannan depolymerization, highly expressed
GH10 endoxylanase (Xyl10a)	TRINITY_DN11072_c0_g2	113670	Involved in xylose depolymerization
GH10b endoxylanase (Xyl10b)	TRINITY_DN17151_c1_g1	105534	Involved in xylose depolymerization
GH3 beta-xylosidase (βXyl)	TRINITY_DN28569_c4_g1	51213	Hemicellulose depolymerization
CE16 carbohydrate esterase (CE16a)	TRINITY_DN26470_c5_g1	125801	Deacetylation of polysaccharides
CE16 carbohydrate esterase family 16 (CE16b)	TRINITY_DN21066_c2_g6	48548	Deacetylation of polysaccharides
GH28 polygalacturonase (Gal28a)	TRINITY_DN7127_c0_g2	111730	Involved pectin depolymerization
Expansins			
Exp1	TRINITY_DN6700_c0_g2	126976	Most likely involved in increasing enzyme accessibility
Exp2	TRINITY_DN24238_c3_g1	128179	Most likely involved in increasing enzyme accessibility

a For identification, the transcriptome ID from our study (strain FPRL 280) and the JGI protein ID (strain MAD 698-R) are used.

### Differential gene expression and functional enrichment within treatments.

Pairwise comparisons of different harvest points within treatments revealed large gene expression differences between the first and later harvest points for all treatments. In the unmodified wood experiment, 2,450 *R. placenta* genes were differentially expressed between weeks 1 and 2, while only 10 genes were differentially expressed between weeks 2 and 3 (Table S5).

The furfurylated wood modification demonstrated a temporal pattern of DE genes similar to that of the unmodified wood experiment (Table S6). For WPG4, there were 103 DE genes between week 3 and week 6 and 481 DE genes between week 3 and week 9. No DE genes were observed between week 6 and week 9. For WPG24, large numbers of DE genes were observed between week 3 and the later time points, but again, there were very few in between the later harvest points. For WPG37, few genes were differentially expressed (between harvest points) in general. However, also here, more DE genes were found between week 3 and the later harvest points and fewer among the later harvest points.

Functional enrichment analyses of the pairwise analyses conformed to the expected two-step decay in this system (Table S7). These results demonstrated that for the unmodified wood experiment, there was an increased expression of functions related to hydrolase and catalytic activity from week 1 to the later harvest points, especially pronounced in weeks 2 and 3 (Table S7). This was also found, but to a lesser extent, in WPG4 and WPG24. In WPG4, most of this response was found between week 3 and week 9, while for WPG24, the response mainly started in the comparison between week 3 and week 12 to week 18. For WPG37, this was not pronounced, and no hydrolase activity was enriched. In the first harvest points of the unmodified wood and WPG4 and WPG24 treatments, we found less enrichment of upregulated functions, with the exception of some upregulation of GO terms related to protein binding and metal ion binding. However, in WPG37, there was an enrichment of several terms related to iron binding, heme binding, oxidoreductase activity, and cytochrome P450s in the upregulated gene set of week 3 compared to week 18. Significant enrichment of a GO term of oxidation-reduction process was found in later stages in WPG4 and WPG24.

As week 1 in the unmodified wood was considered the first initial step of decay, with only 0.8% mass loss, we compared unmodified wood week 1 to all other time points and treatments (Table S8). As with the time series analyses, a signal of downregulation of protein degradation was found in all the WPG37 harvest points compared to unmodified wood. This was seen as enrichment of protein kinases, proteasome, F-box domain, and endopeptidase activity in unmodified wood. This pattern was also found in the early harvest points of WPG24. In contrast, week 1 of the unmodified wood showed upregulation of wood decay-related functions as sugar transporters, GHs, and dehydrogenases.

### Differential expression of specific genes of interest.

**FIG 3:**
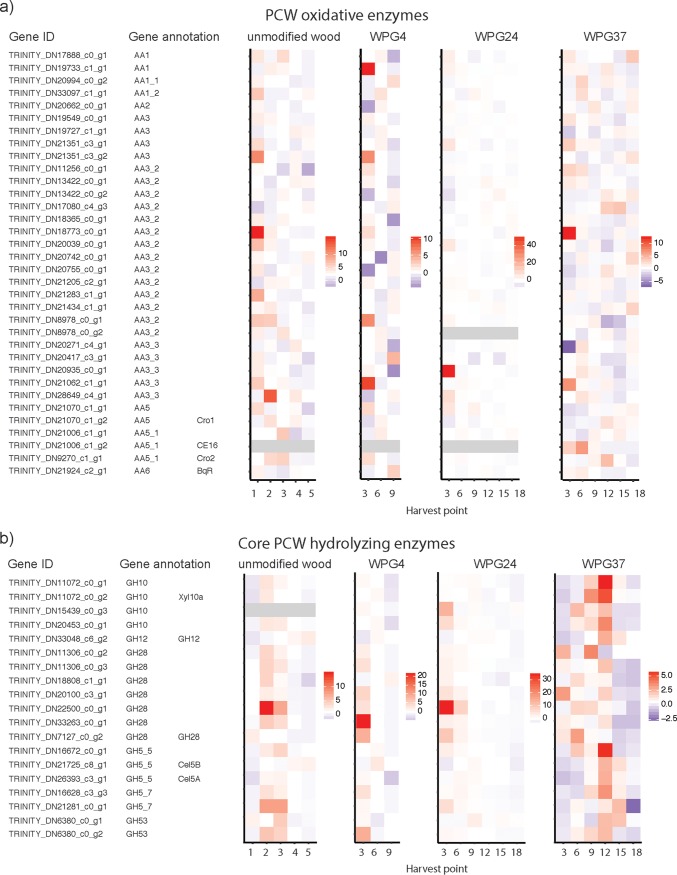

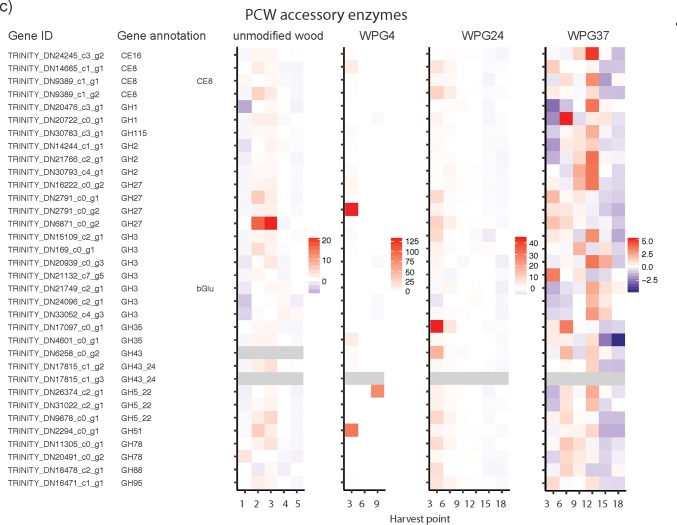
Heatmaps based on dbCAN2 annotations of CAZymes suggested to be involved in plant wall decay, the mean of all replicates from all experiments of *Rhodonia placenta* grown on unmodified radiata pine, and different levels of modification by furfurylated radiata pine. Each experiment is plotted separately, with an independent scale. The gene ID for all transcripts, the dbCAN2 annotations, and the corresponding gene ID from the qPCR are listed. (a) Oxidoreductase enzymes. (b) Core hydrolyzing enzymes. (c) Accessory enzymes suggested to support the core enzymatic apparatus. In the accessory enzyme plot, three genes were removed from the heatmap because of extremely high expression, which hid the signal of the other genes in the unmodified wood plot. See Fig. S2 for the plot with all genes.

The gene expression of annotated carbohydrate-active enzymes (CAZymes) that are involved in wood decay according to Floudas et al. (33) was plotted as heatmaps across treatments (Fig. 3). On unmodified wood, the transcripts predicted to encode the core glycoside hydrolase (GH) enzymes were expressed during intermediate harvest points (weeks 2 and 3), while more of the transcripts encoding oxidoreductase enzymes were more highly expressed early (week 1). In a comparison of the transcripts encoding core enzymes between unmodified wood and WPG37, in both treatments, cellulolytic activity was turned off in the late-decay stages (unmodified wood in week 4 and WPG37 in week 15). The transcripts responsible for the oxidoreductase enzymatic apparatus appear to be turned on for longer and do not have a clear induction time in WPG37 compared to unmodified wood.

### Detailed qRT-PCR analyses of key genes of interest.

Large RNA sequencing (RNA-seq) data sets tend to contain a lot of biological variation, as seen in our principal-component analysis (PCA) plot, which will affect downstream differential expression analyses (Fig. S3). Quantitative reverse transcription-PCR (qRT-PCR) serves as an RNA-seq control but also provides deeper/more-detailed insight into the expression of individual genes. Here, we have used qRT-PCR primers for key genes involved in wood decay and applied a traditional qRT-PCR approach (thus, the same genes as those reported in the cluster analyses; Tables 2, S4, and S9). Plots of both RNA-seq and qRT-PCR data for selected genes are provided in the supplemental material (Fig. S4 to S8). The results below are based on these qRT-PCR data, and RNA-seq data are commented on when the trends deviated from the qRT-PCR data. It is important to keep in mind that RNA-seq and qRT-PCR data are from different wood plugs (*n* = 4 for both data sets), i.e., the same samples are not used for both analyses. Wood samples for each experiment were selected to keep the variation small within experiments. This caused some variation between the experiments. The main effect believed to be reflected in the results is a slightly faster decay for qRT-PCR WPG37 samples than for the RNA-seq WPG37 samples. It is also worth to keep in mind that qRT-PCR included an additional harvest point (week 21).

### Genes involved in oxidative depolymerization: oxalic acid synthesis and oxalate decomposition.

Oxalic acid is assumed to play an important role as an iron chelator and a phase transfer agent in the CMF system (16, 34). The selected genes involved in *R. placenta* oxalic acid metabolism included those for glyoxylate dehydrogenase and oxaloacetate dehydrogenase related to oxalic acid synthesis (GlyD and OahA; Table 2) and oxalate decarboxylase related to oxalate degradation (OxaD; Table 2 and Fig. S4). GlyD was upregulated in unmodified wood compared to furfurylated samples (due to the high expression in unmodified wood at the first harvest point). For unmodified wood and WPG4, GlyD and OahA were upregulated at the first harvest points. For WPG24 and WPG37, increased OahA transcript levels were delayed until the last harvesting points.

Transcript levels of OxaD in qRT-PCR were low in the present study (or not detectable), and no statistically significant trends were found. In contrast, the RNA-seq data showed a strong induction at early-intermediate stages in the different experiments. The comparison with the lowly expressed qRT-PCR data should be interpreted with caution, but the general trends were similar (Fig. S4).

### Genes involved in oxidative depolymerization: redox enzymes.

Extracellular peroxide generation is a key component of oxidative lignocellulose degradation. The selected genes illustrated in Fig. S5 assumed to be involved in the early oxidative stage of *R. placenta* decay included three glucose-methanol-choline (GMC) oxidoreductases (CAZy family auxiliary activity 3 [AA3]; AOx1, AOx2, AOx3, and AOx4; Table 2), two copper radical oxidases (Cro1 and Cro2; Table 2), and a benzoquinone reductase (BqR; Table 2).

GMC oxidoreductases are flavoenzymes that oxidize a wide variety of alcohols and carbohydrates, with concomitant production of hydrogen peroxide or hydroquinones (35). In the present study, AOx1 and AOx2 were upregulated in WPG37 compared to unmodified wood, and for AOx2, WPG24 was also upregulated. AOx3 was highly expressed, but no significant differences in expression were found within (except an upregulation in WPG24 week 3) or between treatments. AOx4 showed low expression levels.

Copper radical oxidases (CAZy family AA5) are widely distributed among wood decay fungi (36), oxidize a variety of substrates, and produce H_2_O_2_. In the present study, Cro1 and Cro2 were upregulated at week 1 for unmodified wood and week 21 for WPG24. Since RNA-seq was not performed beyond week 18, this trend was not confirmed. Cro2 was upregulated in WPG24 compared to WPG4.

Benzoquinone reductases (CAZy family AA6) are intracellular enzymes involved in oxidative depolymerization of wood cell wall polymers by the synthesis of hydroquinone compounds that can act as mediators for laccases or reductants of iron in brown rot systems (15, 37). In the present study, BqR was upregulated in later stages of decay both for unmodified wood and for WPG37.

### Hydrolytic enzymes involved in polysaccharide depolymerization: hemicellulose and pectin degradation.

Figure S6 illustrates the selected genes involved in hemicellulose hydrolysis. One endomannanase in CAZy family GH5 (Man5a; Table 2) was found to be upregulated in unmodified wood versus WPG37. All treatments showed downregulation with increasing incubation time.

For the two endoxylanases in CAZy family GH10 (Xyl10a and Xyl10b; Table 2), there was a tendency for upregulation in furfurylated wood at 3 weeks incubation. For RNA-seq, there was a tendency for upregulation in unmodified wood at week 2.

Beta-xylosidase in CAZy family GH3 (βXyl, Table 2) hydrolyzes 1,4-β-d-xylans and xylobiose. βXyl was upregulated in WPG37 compared to WPG4. In unmodified wood, there was an upregulation at week 3 compared to week 5, while for furfurylated wood, there was an upregulation in WPG24 and WPG37 at the last harvest point (week 21).

Carbohydrate esterases catalyze the de-*O*- or de-*N*-acylation of substituted saccharides. The selected carbohydrate esterase 16a (CE16a; Table 2) was upregulated in unmodified wood compared to WPG4 and WPG37. All treatments showed downregulation with increasing incubation time. The carbohydrate esterase CE16b (Table 2) provided very low expression levels with qRT-PCR (especially unmodified wood and WPG4). A clearer trend was found with RNA-seq, especially for WPG24 and WPG37, with decreasing expression levels with increasing incubation time.

Polygalacturonase Gal28a (Table 2) hydrolyzes the alpha-1,4 glycosidic bonds between galacturonic acid residues (pectin). Gal28a was upregulated in unmodified wood compared to WPG4 and WPG37. All treatments showed downregulation with increasing incubation time.

### Hydrolytic enzymes involved in polysaccharide depolymerization: cellulose degradation.

The endoglucanases Cel5a and Cel5b (Table 2) cause chain breaks in amorphous cellulose. Cel5a was upregulated in WPG4 versus WPG37, and Cel5b was upregulated in unmodified wood and WPG4 versus WPG24 and WPG37 (Fig. S7). The expression in unmodified wood increased with increasing incubation time.

GH12 glucoside hydrolase (XyGEg; Table 2) shows high sequence similarity to several endoglucanases with activity on cellulose and xyloglucan. This enzyme was upregulated in WPG4 versus WPG37. In unmodified wood samples, it was a gradual increase in XyGEg with increasing incubation time.

GH3 beta-glucosidase (βGlu; Table 2) releases glucose by hydrolysis of cellobiose and was upregulated in unmodified wood and WPG4 versus WPG37. In WPG24 and WPG37, there was an upregulation at week 9 versus week 21.

Lytic polysaccharide monooxygenases (CAZy AA families 9, 10, 11, 13, 14, and 15) are important enzymes in lignocellulose depolymerization that cause oxidative chain breaks in crystalline and amorphous polysaccharides. We selected a cellulose-active AA9, lytic polysaccharide monooxygenase (LPMO; Table 2). For qRT-PCR, no statistically significant trends were detected for this gene except for upregulation at week 3 in WPG37.

### Expansins with a possible role in loosening plant-cell wall interactions.

Expansins are hypothesized to increase enzyme accessibility by loosening plant-cell wall interactions, although no catalytic mechanism is known (38–40). The two selected genes predicted to encode expansins (Exp1 and Exp2; Table 2) are given in Fig. S8. The expression patterns of the two expansins did not show a clear trend, but for Exp1 in WPG37, the expression was upregulated at weeks 3 and 9 compared to later decay stages.

## DISCUSSION

We have investigated if wood modification by furfuryl alcohol causes altered decomposition response in *Rhodonia* (*Postia*) *placenta*, using RNA-seq and qRT-PCR, in unmodified and furfurylated radiata pine wood.

From our four treatments (unmodified wood and the three levels of modification with furfuryl alcohol polymer), we found that WPG4 closely followed the trend on unmodified wood, while WPG24 seemed to be an accelerated version of the decay pattern of WPG37. For simplicity, we mainly focus on unmodified wood decay processes and the comparison of unmodified wood decay versus WPG37 decay in the Discussion.

In our unmodified wood decay experiments, we observed wood decay from incipient stages with mean mass loss of 0.8% in week 1, to severely decayed wood with a mean mass loss of 29% at week 5. Our results support the previously suggested two-step decay mechanism of brown rot fungi (13, 16, 18, 41), i.e., an oxidative nonenzymatic system followed by hydrolytic degradation. Week 1 of unmodified wood demonstrated an early decay transcriptome response supporting a Fenton-like reaction, with higher expression of oxidoreductase enzymes, polygalacturonase GH28, and oxalic acid synthesis genes (Fig. 3 and Table 2) in both the RNA-seq data and for the genes specific subject to qRT-PCR (Fig. 3 and S5).

This early response was followed by a polysaccharide degradation stage, with upregulation of transcripts encoding hemicellulases, e.g., endoxylanases (Xyl10b and Xyl10a), beta-xylosidase (βXyl), and endomannanase (Man5a), in weeks 2 and 3. In our study, there was an abrupt decline in transcripts encoding many core hemicellulases in week 4 during the later stages of decay (Fig. 3 and S7; Xyl10a, Xyl10b, βXyl, and Man5a). This is in agreement with observations made by Zhang and Schilling (18). It has been demonstrated that soluble sugars, in particular, cellobiose, act as the main inducing agent in the switch from oxidative (week 1) to hydrolytic depolymerization (18, 42).

Some transcripts encoding putative cellulases are also accumulated in week 2 and were further upregulated in week 3. This was particularly obvious from the qRT-PCR expression pattern of the GH3 beta-glucosidase (βGlu) and the LPMO (Fig. S7). Some cellulase transcripts are also highly expressed in the more advanced stages of decay, e.g., both the endoglucanase GH5 (Cel5b) and the glucoside hydrolase GH12 (Cel12a) are most highly expressed in week 5. This high expression of cellulases at a late decay stage was also observed by Zhang and Schilling (18). Thus, when the more easily accessible hemicelluloses have been degraded, expression of cellulases Cel5b and Cel12a is maintained at a high level, well into the phase where the fungus is experiencing starvation (discussed below).

When the decay of the unmodified wood was compared to the highly modified wood, we found large differences in the timing of gene expression. From previous studies, it is known that gene expression of *R. placenta* and other decay fungi is influenced by the wood substrate. For example, Vanden Wymelenberg et al. (24) found that 164 genes exhibited significant differences in transcript accumulation in a comparison of aspen (Populus grandidentata) and pine (Pinus strobus) as the substrates. Furthermore, for this fungal species, gene expression profiles were influenced by wood substrate composition between wood genotypes of a single species (Populus trichocarpa) and the duration of incubation (32). Hence, it is expected to find differences in response when the radiata pine substrate is modified (in this case, furfurylated).

We observed that transcripts encoding oxidoreductase enzymes were highly expressed in the first harvest point (week 3) as well as for the modified WPG37. This confirms the finding in Alfredsen et al. (27) where *R. placenta* expressed high levels of oxidoreductase enzymes and produced oxalic acid during 8 weeks colonization of furfurylated Scots pine. However, the patterns of repression of these oxidative processes and the oxalic acid synthesis found in unmodified wood at later stages are less clear (Fig. 3). This is particularly obvious in a comparison of the expression of transcripts encoding oxalate decarboxylase (OxaD) and AA3 GMC oxidoreductase (AOx1), which showed a distinct time of induction on unmodified wood but not on WPG37, where elevated expression was randomly distributed over time between samples. This can conceivably be explained by repeated exposure to regions with heavily modified substrate, thus reinducing oxidative processes to overcome furfurylation in order to expose degradable substrate to the fungus allowing further growth, or a direct response to the furfuryl polymer.

However, the strong induction of transcripts encoding core hydrolyzing enzymes and accessory enzymes observed in week 2 and 3 in unmodified wood seemed comparable to that in WPG37 from weeks 6 to 12 and, partially, week 15, as observed by the RNA-seq CAZyme analyses and the qRT-PCR analyses (Fig. 3 and S7). Thus, the switch to turn on the core hydrolyzing enzymes in the intermediate decay stages was observed in both untreated and furfurylated wood in our experiments; thus, we hypothesize that furfurylation does not influence the availability of these soluble sugars to such an extent that it inhibits induction. Previous studies have shown that *R. placenta* grown on wood modified with both furfurylation and acetylation shows similar or decreased levels of core wood-hydrolyzing enzymes (25, 27). Our study, with a longer time series, does not suggest reduced expression of these genes but rather a delayed and elongated process. The elongated process could also be the reason for the placement of all the specific plant cell wall (PCW)-related transcripts in the large cluster with no obvious induction time in WPG37. The longer incubation times used in this study and the elongated processes can explain why this trend was never observed in previous studies. Thus, based on the observation that a delayed but similar pattern was observed in both the unmodified wood and WPG37 experiments, we conclude that the furfurylation does not directly influence the expression of these core PCW-degrading enzymes.

There are several hypotheses regarding the mode of action of modified wood against brown rot decay fungi (43, 44), which are summarized in reference 45. Briefly, the authors of reference 45 hypothesize that unavailability of easily accessible nutrients (43, 44), enzyme nonrecognition (7), micropore blocking (46), and moisture exclusion due to OH group blocking/reduction (44) and/or reduction in void volume may be important modes of action (47, 48). In our study, we found no evidence that supports the enzyme nonrecognition hypothesis, but more data are needed to reject the hypothesis. The delay in gene expression in our study can be explained by initial blocking of wood polysaccharides by furfuryl alcohol polymers compared to unmodified wood. In addition, a further delay in decay of modified wood versus unmodified wood is expected due to lower wood moisture content in the modified substrate. Moreover, the strong enrichment of transcripts related to salt stress genetic functions in the WPG37-K1 clustering that was not found in the other treatments may support the conclusions by Ringman et al. (45) that moisture exclusion may be an important mode of action in this modification method.

As a support for the notion that the furfurylation is nontoxic for the fungus, we found no enrichment of genetic functions directly related to defense mechanisms in the modified wood compared to unmodified wood. *Rhodonia placenta* is known to be highly tolerant to substances such as copper, mainly due to the ability to produce oxalic acid that chelates and precipitates copper and other transition metals (49). However, in addition to these more general functions that are difficult to separate from wood decay mechanisms, copper-tolerant fungi are also known to express catalases and ATP pumps related to copper transport in response to toxins (50). These functional categories were not enriched in the differentially expressed gene sets in our study in a comparison of WPG37 to the unmodified wood, indicating that the fungus is not experiencing a more toxic atmosphere in the modified wood.

Notably, the most pronounced transcriptome difference in the unmodified wood compared to the modified wood is the strong induction of functions related to the ubiquitin/proteasome (U/P) pathway, protein degradation, and the Ras pathway. All of these functions are related to carbon starvation and are highly enriched in the late decay of the unmodified wood. Thus, we hypothesize that *R. placenta* growing on unmodified wood is starving and that the fungus has consumed the majority of available carbon sources in the wood in the latest harvest points of the unmodified wood experiment. This is further supported by the downregulation of CAZymes in the last 2 weeks in this experiment.

The ubiquitin/proteasome (U/P) pathway is a conserved pathway in all eukaryotic organisms and is important to various cellular processes, such as recycling of intracellular protein (where unnecessary proteins are degraded to amino acids that can be reused to produce new proteins) and programmed cell death. Recently, the pathway was shown to be activated by carbon starvation in the ectomycorrhizal basidiomycete species Paxillus involutus (51). In P. involutus, 45% of the transcripts were differentially regulated during carbon starvation. This large response is also shown in our study, where most enriched functions in unmodified wood compared to the WPG37 for the time series analyses could be connected to the U/P or the Ras pathway. This could reflect the higher need for translocation of nitrogen-rich resources in the unmodified wood substrate to areas that need new protein synthesis than is the case in WPG37 or simply reflect a general starving response due to a lack of substrate.

The other functions observed to be upregulated in unmodified wood compared to WPG37 were several domains related to Ras proteins. These proteins are involved in cell proliferation and growth of the mycelia. Carbon starvation supports an upregulation of transcripts encoding these proteins. In starving mycelia, it has been shown that the diameter of the hypha is reduced while it grows to cover a larger area (51, 52). Ras proteins have been shown to enhance the formation of pseudohyphal growth in starving yeast cultures. These pseudohyphae are thin long cells extending away from the culture, searching for nutrients (53). Ras proteins might well be involved in a change in growth to search for more nutrients in starving mycelia across the fungal kingdom.

In all our experiments, we investigated one strain, FPRL 280. Thaler et al. (54) suggested significant differences in the regulation of key lignocellulose-degrading enzymes between the previously sequenced MAD 698-R strain and the FPRL 280 strain used in this work. This proposed difference is supported by the current study, with a mapping success of only 40 to 70% when the reads of European strain FPRL 280 were mapped to the genome of MAD 698-R. The difference might have been present since the time of isolation, or (less likely) the changes might have occurred during storage of these strains. Divergence between fungal populations across continents, leading to cryptic speciation, has been suggested for several wood decay fungi (e.g., Fomitopsis pinicola [55] and Serpula himantioides [56]). This highlights the importance of providing verifiable strain information when publishing decay studies and in a comparison of American versus European wood decay testing.

Furthermore, for decay testing, the sample size, sample geometry, and wood anatomy influence the colonization rate. Sampling time and extent of decay are crucial when examining gene expression or secretome of fungal colonization of wood. The studies by Zhang et al. (13) and Zhang and Shilling (18) used thin wafers and harvested at different distances from the *R. placenta* hyphal front. This wafer approach works well to separate different initial decay stages and provides information about spatial separation along the decay gradient. For more advanced stages of decay, a different approach is needed. In the current study, we selected small and homogeneous (earlywood) samples in order to enable colonization on a uniform solid wood substrate for decay as fast as possible. Still, some variation in gene expression is expected, as new colonization pathways will be found close to areas with previously colonized wood. This effect is expected to increase with increasing WPG level, since areas that are long colonized will cease to provide nutrients able to sustain survival, while the others that are more recently invaded will provide nutritious substrates. In our experiments, the wood modification treatment modifies the composition of the wood, and the fungus is therefore forced to respond differently from when it encounters unmodified wood. Hence, comparison at similar mass losses between treatments was not the goal of this study, but rather, it was the shift of gene expression over time between and within the different treatments.

The novelty of this study compared to previous work on transcriptome studies of fungal decay of modified wood (including furfurylation) is the combination of (i) following the entire transcriptome instead of just a few selected genes as in previous studies of modified wood and (ii) including decay stages from initial decay to advanced stages of decay. Hence, this is a significantly more comprehensive data set than used in previous studies. The major findings based on this approach were (i) the identification of a decay threshold for the highest treatment level (WPG37) and (ii) that *R. placenta* colonizing furfurylated wood follows the same modality of gene expression as for unmodified radiata pine wood but with a delayed and elongated response (increasing delay with increasing furfurylation level).

Increased knowledge about brown rot decay mechanisms is important for an expanded understanding of the fungal decay process in general since ecosystem carbon flows are closely linked to wood-degrading fungi. From an industry perspective, the findings in this study show that successful inhibition of the initial oxidative decay is a clue to success for future wood protection systems.

In the present study, the entire gene expression pattern of a decay fungus is followed in untreated and modified wood from initial to advanced stages of decay. From these observations, we have demonstrated how the furfurylated modification delays the fungus gene expression while growing on this substrate. All treatments (modified and unmodified) expressed the expected staggered decay mechanism, i.e., enzymes implicated in oxidative decay were expressed first, and then hydrolytic enzymes were expressed later. Thus, we show that the fungus follows the same modality of gene expression on the modified wood. The major difference is that the responses are delayed and elongated compared to unmodified wood. However, from the downregulation of CAZymes in the latest harvest points in the WPG37, the fungus seems to be finalizing the decay also in the furfurylated wood at a mass loss of only 14%, compared to 29% in the unmodified wood. This suggests that the fungus never gets access to the remaining carbohydrates from the modified wood even at low mass loss. The lower levels of modification in the WPG4 and WPG24 treatments show intermediate expression differences and higher mass loss. The exact mode of action of the modification is still uncertain. However, the similar decay mechanisms and no obvious signs of gene expression related to detoxification of the furfuryl alcohol may indicate that the furfurylation functions as both a physical barrier and a factor that creates a less-hydrated environment for the fungus. These hypotheses should be investigated to further improve environmentally friendly modification processes. Further studies could include studies of chemical, mechanical, and structural properties of the decaying wood cell wall at a nanoscale using novel microscopy techniques, for example, combining atomic force microscopy and infrared spectroscopy.

## MATERIALS AND METHODS

### Wood material.

In order to get wood material as homogeneous as possible, plugs (Ø = 6 mm, h = 10 mm) were prepared from radiata pine (*Pinus radiata* D. Don) earlywood according to Beck et al. (57). The boards were provided by Kebony ASA, Skien, Norway. Before treatment, all samples were dried at 103°C for 18 h and then cooled in a desiccator before the initial dry weight was recorded. The furfurylation process was performed with three different concentrations of furfuryl alcohol, synthesis grade >98% (Merck, Darmstadt, Germany), according to the formula by Kebony, with furfuryl alcohol-to-water ratios of 7:10 (WPG37) in the commercial treatment level, 4:10 (WPG24), and 1:10 (WPG4). The samples were left soaked in the furfuryl alcohol solutions for 15 days. Sets of five samples were wrapped in aluminum foil and cured at 120°C for 16.5 h. All samples including the unmodified wood were leached according to EN 84 (69) as a preweathering test and dried at room temperature. In order to provide weight percent gain (WPG) and initial dry mass after treatment, the samples were dried at 103°C for 18 h and then cooled in a desiccator before the dry weight was recorded. The samples were left in a climate chamber at 65% relative humidity and 20°C until stable weight before they were wrapped in sealed plastic bags and sterilized by gamma irradiation (25 kilogray [kGy]) at the Norwegian Institute for Energy Technology.

### Decay test.

The brown rot fungus in this experiment was Rhodonia placenta (Fr.) Niemelä, K.H. Larss. & Schigel (syn. Postia placenta) strain FPRL 280 (= BAM113). The strain was obtained from Bundesanstalt für Materialforschung und –prüfung, 4.1 Division Biodeterioration and Reference Organisms (BAM), as a dikaryotic strain. The fungus was first grown on 4% (wt/vol) Difco malt agar medium (VWR), and plugs from actively growing mycelia were transferred to a liquid culture containing 4% (wt/vol) Difco malt (VWR). After 2 weeks, the liquid culture was homogenized with a tissue homogenizer (Ultra-Turrax T25; IKA Werke GmbH & Co. KG, Staufen, Germany).

A modified E10-16 soil/block test (20) was used. Agar plates (TC dish 100, standard; Sarstedt AG & Co., Nümbrecht, Germany) (Ø = 87 mm, h = 20 mm) containing soil (2/3 ecological compost soil and 1/3 sandy soil) were adjusted to 95% of their water-holding capacity according to ENV 807 (70). A plastic mesh was used to avoid direct contact between the samples and the soil. A 300-μl inoculum of homogenized liquid culture was added to each sample. Eight samples of the same treatment were added to each plate, and four replicate plates were used.

Samples were incubated at 22°C and 70% rH and harvested every third week in experiments with modified wood and every week for the unmodified wood material. Fungal mycelium was manually removed from the wood surface with delicate task wipes (Kimtech Science, UK) in order to remove most of the fungal mass for the wood mass loss measures. Eight samples from each treatment and each harvesting point were dried at 103°C for 18 h in order to provide data for mass loss (mean WPG for WPG4, 3.8% ± 0.7%; WPG24, 24.6% ± 4.1%; and WPG37, 36.1% ± 5.5%). The remaining samples were wrapped individually in aluminum foil and put directly into a container with liquid nitrogen. The samples were then stored at −80°C.

For RNA-seq analyses, samples from the following harvesting points were used: unmodified wood, weeks 1 to 5; WPG4, weeks 3, 6, and 9; and WPG24, weeks 3, 6, 9, 12, 15, and 18. For qRT-PCR, samples from the following harvesting points were used: unmodified wood, weeks 1, 3, and 5; WPG4, weeks 3, 6, and 9; WPG24, weeks 3, 9, 15, and 21; and WPG37, weeks 3, 9, 15, and 21. The harvest interval was shorter for the experiments on unmodified wood, as this wood was known to decay more rapidly. Furthermore, for WPG4, the experiment was ended after 9 weeks because of severe mass loss at this stage.

### RNA purification and cDNA synthesis.

Wood powder from frozen samples was obtained by cutting the plugs into smaller pieces with garden shears wiped with 70% alcohol and, thereafter, Molecular BioProducts RNase AWAY surface decontaminant (Thermo Scientific, Singapore). The wood samples were immediately cooled again in Eppendorf tubes in liquid nitrogen. Fine wood powder was produced in a 300 mill (Retsch mbH, Haan, Germany). The wood samples, the 100-mg stainless steel beads (Qiagen, Hilden, Germany), and the containers were chilled with liquid nitrogen before grinding at maximum speed for 1.5 min. They were then cooled in liquid nitrogen again before a second round of grinding.

For Illumina sequencing, the MasterPure plant RNA purification kit (Epicentre, Madison, WI, USA) was used, according to the manufacturer’s instructions, for total RNA extraction with a 100-mg wood sample. The mean WPGs for the four replicate samples were WPG4, 3.6% ± 0.5%; WPG24, 24.8% ± 1.2%; and WPG37, 39.7% ± 2.2%.

For qRT-PCR, the MasterPure complete DNA and RNA purification kit (Epicentre, Madison, WI, USA) was used, according to the manufacturer’s instructions, with a 90-mg wood sample (a different kit was used because the kit used for Illumina analyses was no longer produced). The mean WPGs for the four samples were WPG4, 4.1% ± 0.4%; WPG24, 20.9% ± 0.9%; and WPG37, 33.7% ± 3.8%. A NanoDrop 2000 spectrophotometer (Thermo Scientific, Singapore) was used to quantify RNA in each sample. To convert RNA to cDNA, the TaqMan reverse transcription reagent kit (Thermo Scientific) was used according to the manufacturer’s instructions. The total reaction volume was 50 μl. Three hundred nanograms of RNA was reacted with oligo(dT_16_) primer in RNase-free water (Qiagen, Hilden, Germany). The solution was incubated for two cycles in a PCR machine (GeneAmp PCR system 9700; Applied Biosystems, Foster City, CA, USA) at 65°C for 5 min and 4°C for 2 min. The PCR machine was paused and the master mix added. The next three cycles included 37°C for 30 min, 95°C for 5 min, and 4°C for an indefinite time. In addition to the test samples, two samples without RNA were added as controls and used for each primer pair. After the cDNA synthesis, 50 μl RNase-free water (Qiagen) was added to the samples and mixed well.

### RNA-seq sequencing, quality control, and trimming.

All Illumina libraries and sequencing were performed by the Norwegian Sequencing Centre (https://www.sequencing.uio.no/). All samples were prepared with the strand-specific TruSeq RNA-seq library preparation method. In order to produce a high-quality *de novo* transcriptome, we sequenced the strain grown on a 4% (wt/vol) Difco malt agar medium (VWR) in addition to 10 selected samples spanning the entire experimental setup on one lane on the HiSeq 2500 platform, producing 125-bp paired-end sequences. These libraries generated ∼395 million read pairs, which after quality control and trimming were reduced to ∼250 million read pairs.

For the experiment itself, we collected 80 samples, of which three failed library preparation, resulting in 77 NextSeq samples in total. The sequenced libraries generated ∼28 million trimmed reads on average.

The quality of the reads was evaluated using FastQC version 0.11.2 (58) and trimming performed with Trimmomatic version 0.36 (59), with the following parameters for the NextSeq samples: TruSeq3-SE.fa:2:30:10 MAXINFO:30:0.4 MINLEN:30. For the HiSeq 2500 samples, the built-in Trimmomatic option in Trinity for the NextSeq samples 2.2.0 (60) was used with the following parameters: TruSeq3-PE.fa:2:30:10 SLIDINGWINDOW:4:5 LEADING:5 TRAILING:5 MINLEN:30 MAXINFO:30:0.4.

### Transcriptome assembly and evaluation.

We initially attempted to use the *R. placenta* genome generated from an American *R. placenta* strain in our analysis (Postia_placenta_mad_698, full fasta sequence [soft masked]) from the Ensembl Fungi FTP server. The genome-based attempt using Bowtie 2, TopHat 2, and Cufflinks resulted in poor map back (∼50 to 70%) despite doing parameter sweeps (data not shown). We also attempted to use the genome-guided option of the *de novo* transcriptome assembler Trinity that yielded a more fragmented transcriptome compared to the clean *de novo* version (data not shown).

The final transcriptome was generated using a *de novo* strategy with the HiSeq trimmed reads and Trinity version 2.2.0 (seqType fq, max_memory 250G, SS_lib_type RF, CPU 20, bflyHeapSpaceMax 10G and bflyCPU 20) on our local computing cluster. The Trinity bfly process failed for 110 elements which were rerun with more memory. The finished transcriptome contained 56,520 contigs, with a total of 114,539 transcripts, including isoforms (Table S1).

We also evaluated the assembly using BUSCO version 2.0 using the recommended parameters and the corresponding fungal database created on 26 January 2017 with 85 species and 290 BUSCO genes (61). Of the 290 BUSCO genes, 288 were found to be complete (99.3%) (Table S2).

### Assembly annotation using Trinotate and the JGI Fungi portal.

The assembly was subjected to the Trinotate annotation pipeline, according to the manual, which provided a generic annotation of assembly. However, as there is a study (13) looking into key wood-degrading genes using the annotation from the *R. placenta* genome, we manually annotated our assembly using the predicted proteins reported at the JGI Fungi Portal (https://genome.jgi.doe.gov/mycocosm/home) using TBLASTN from BLAST+ (62), with an E value cutoff of 1e−50. Finally, we ended up with a custom annotation being a hybrid of the generic Trinotate annotation where identifiers from the *R. placenta* genome have been added.

### Mapping and abundance estimation of NextSeq samples.

Mapping was performed using the built-in mapping option in Trinity version 2.3.2 with the RSEM count estimation method, the Bowtie alignment method, and specifying SS_lib_type R. The counts were collected per “gene” using the abundance_estimates_to_matrix.pl script in Trinity and the resulting count matrix used for differential gene expression in R.

### Differential gene expression analysis.

In R version 3.4.1, the overall data were initially explored using VariancePartition version 1.8 (63). Differential gene expression analyses were performed using edgeR version 3.20.1 (64, 65). In addition, some of the plot functions in DESeq2 version 1.18.0 (66) were used to explore those overall data and to plot raw counts.

The overall workflow with code is available in the supplemental information, but briefly, we followed the steps described below:

The colData object describing the overall experiment was set up with time, treatment, mass loss, and condition, where condition in practice is the intercept between time and treatment. We initially filtered on counts per million (cpm; cutoff of 1 in at least 2 libraries) and set a full model consisting of treatment, time, condition, and mass loss to explore the data in VariancePartition. We also made heatmaps using pheatmap version1.0.8 and log_2_-transformed count data.

Multifactorial differential expression analysis was performed in both edgeR and DESeq2 (the latter to enable use of various plot functions). In DESeq, we opted for a ∼treatment+time formula. Running a more complex design with interaction (:) was not possible, as DESeq2 cannot handle partial models (time points for the different treatments do not overlap completely). Furthermore, we opted for the likelihood ratio test (LRT), which examines two models for the counts, the full model and a reduced model. This then determines if the increased likelihood of the data using the extra term(s) in the full model is more than expected.

In edgeR, we opted for an ∼0 + treatment+time formula without generating an intercept term. When estimating overall dispersion, we used the robust=TRUE option to better handle outliers in the data. For the multifactorial test itself, we chose to replace the standard glmFit with glmQLFit, which uses a quasi-likelihood F-test on the likelihood ratio statistics instead of the chi-square approximation. In this way, we should obtain a more conservative control of the type I error rate, as it takes into account the uncertainty in estimating dispersion for each gene, especially when the number of replicates is small.

To enable an exploration of specific contrasts between given conditions, we also ran an ∼0 + condition formula in edgeR using the same setup as described above.

We evaluated each treatment versus the unmodified wood over time from the treatment+time multifactorial analysis. For the pairwise contrasts between harvest times within treatments, we used the ∼condition multifactorial analysis.

The overall exploration of the data revealed that the WPG24 treatment series has a few outliers. RNA-seq data are highly variable, and the individual variation between replicates can be large. We found that 2 replicates in the WPG24 3-week condition were relatively deviating from the other two replicates. We reran the above-described analysis without the most extreme outlier (WPG24, 3 weeks, replicate 2) and observed a large change in the number of reported significant differentially expressed genes. However, seeing as these differences did not necessarily improve the results, we decided to keep all samples in the final data set.

### Clustering using MBCluster.Seq.

As an alternative to the pairwise differential expression analyses, the read count data were clustered based on similarity in expression patterns using the MBCluster.Seq package in R. The EM clustering algorithm was used.

### Functional summary.

Functional enrichment analysis was used to characterize the functions of the differential expressed genes of the various treatments and clusters. The functional enrichment of PFAM domains and GO terms in the DE gene sets was calculated using a Fisher’s exact test with Benjamini-Hochberg correction for multiple testing.

### CAZyme annotations.

The transcripts were translated using TransDecoder (https://github.com/TransDecoder/TransDecoder/wiki). All protein sequences were annotated in dbCAN2 (67). The standardized (median/median absolute deviation) gene expression patterns of the resulting annotated transcripts were plotted in a heatmap using R (68).

### qRT-PCR.

The qRT-PCR-specific primers used to determine the transcript levels of selected genes were designed with a target melting temperature (*T_m_*) of 60°C and to yield a 150-bp product. qRT-PCR was performed using ViiA 7 by Life Technologies (Applied Biosystems, Foster City, CA, USA). Primers were designed using Primer3 (http://bioinfo.ut.ee/primer3-0.4.0/primer3/), using the genome of *Postia placenta* MAD 698-R as the template (https://genome.jgi.doe.gov/Pospl1/Pospl1.home.html). Target genes were selected based on existing transcriptomic data of genes known to be upregulated during decay by *R. placenta* MAD 698-R (13). The genes chosen are hypothesized to be involved in either oxidative or hydrolytic depolymerization of wood cell wall components. The master mix included for each sample 5 μl Fast SYBR green master mix (Thermo Scientific, Singapore), 0.006 μl of 10 μM forward primer, 0.006 μl of 10 μM reverse primer, 2.88 μl of RNase-free water (Qiagen, Hilden, Germany), and 2 μl of test sample (total volume, 10 μl). The qRT-PCR run included the following stages: hold stage with an initial ramp rate 2.63°C/s and then 95.0°C for 20 s. PCR conditions were 40 cycles of initial ramp rate of 2.63°C/s, 95.0°C for 15 s, ramp rate of 2.42°C, followed by 60.0°C for 20 s. The melt curve stage had an initial ramp rate of 2.63°C/s, and then 95.0°C for 15 s, a ramp rate of 2.42°C/s, 60.0°C/s, and then 0.05°C/s.

Two constitutive housekeeping genes, β-tubulin (βt; 113871) and α-tubulin (αt; 123093), were used as a baseline for gene expression. The target genes (Tg) and the endogenous controls in this study are listed in Table 2. Protein identification (ID) was according to the *Postia placenta* MAD 698-R v1.0 genome from the Joint Genome Institute (https://genome.jgi.doe.gov/pages/search-for-genes.jsf?organism=Pospl1). Threshold cycle (*C_T_*) values obtained here were used to quantify gene expression.

The software used to export the *C_T_* values was the QuantStudio real-time PCR system (Applied Biosystems by Thermo Fisher Scientific, Foster City, CA, USA). The *C_T_* values of βt, αt, and Tg were used to quantify gene expression according to the following equation: $10^{4}\times^{2C_{T}\beta \text{t}-C_{T}\text{Tg}}$, giving an arbitrary baseline expression of β-tubulin and α-tubulin of 10^4^. As an internal control, the expression levels of βt and αt were compared using the same equation, showing a stable expression, with αt being expressed at approximately 80% relative to βt. Only data for βt were included in this paper.

### Statistical analysis of qRT-PCR.

All statistics were performed in JMP (version Pro 13; SAS Institute, Inc., Cary, NC, USA). The significance of differences in expression levels of each gene was calculated with Tukey’s honestly significant difference (HSD) test. A probability of ≤0.05 was the statistical type I error level.

### Data availability.

RNA-seq data generated for this study were deposited at the European Nucleotide Archive (ENA) under accession number PRJEB32334. The transcriptome assembly and all lists of differentially expressed genes were deposited in Dryad at https://doi.org/10.5061/dryad.vc6s461.

## Supplementary Material

Supplemental file 1
