# Supplementary material for: Wood Modification by Furfuryl Alcohol Resulted in a Delayed Decomposition Response in Rhodonia (Postia) placenta
Source: Appl Environ Microbiol. 2019 Jul 1;85(14):e00338-19. doi: 10.1128/AEM.00338-19 (PMC6606883; doi:10.1128/AEM.00338-19)
Supplement: Supplemental file 1 [file AEM.00338-19-s0001.pdf]

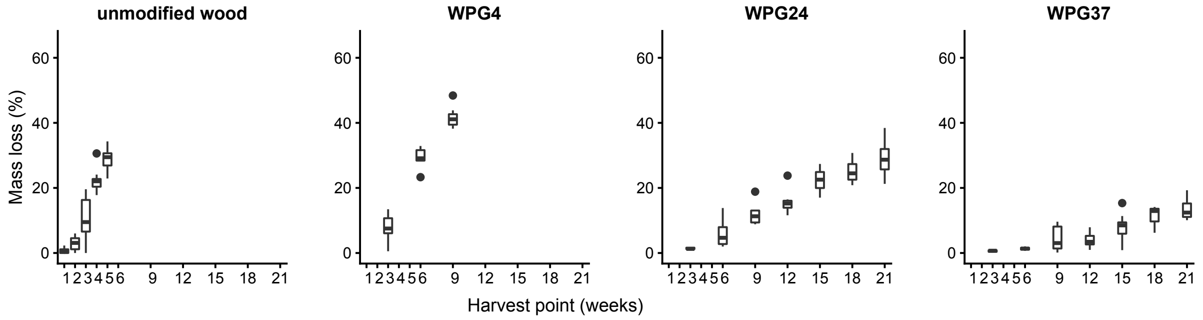

Supplementary Figure 1. Boxplots of the mass loss of all experiments of *Rhodonia placenta* grown on *Pinus radiata* and different levels of modification by furfurylated *P. radiata*. The weight of the treatment is excluded from the measurements. a) *R. placenta* grown on unmodified P. radiata. Wood harvested at five different harvest points (week). b) *R. placenta* grown on furfurylated *P. radiata*, Weight Percent Gain (WPG) 4%. Wood harvested at three different harvest points. For c) and d) The wood was harvested at six harvest points for *R. placenta* grown on furfurylated *P. radiata*, WPG 23% (c) and WPG 37% (d).

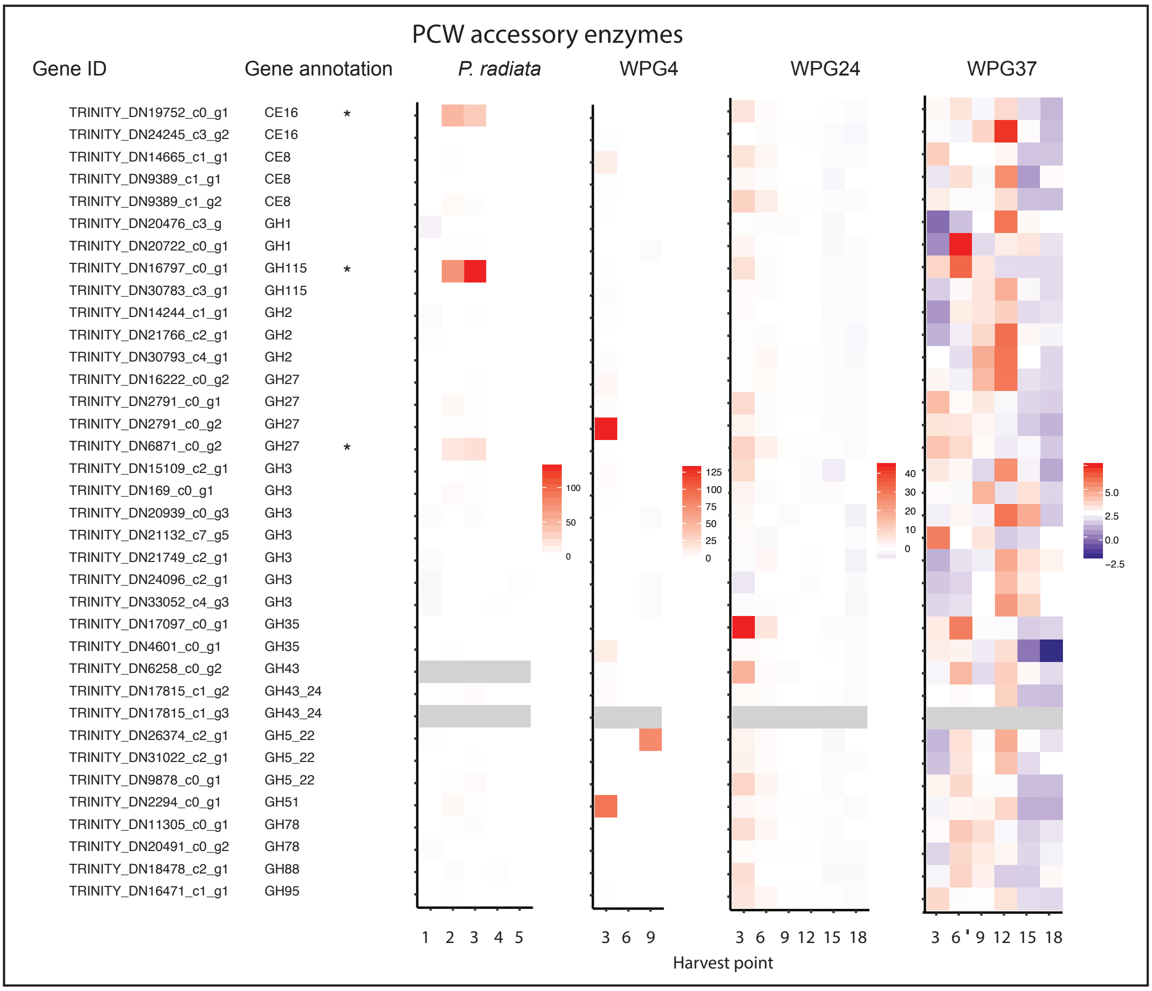

Supplementary Figure 2. Heatmaps based on dbcan2 annotations of accessory CAZymes suggested to be involved in plant wall decay the mean of all replicates from all experiments of *Rhodonia placenta* grown on *Pinus radiata* and different levels of modification by furfurylated *P. radiata*. Each experiment is plotted separately, with an independent scale. The Gene ID for all transcripts, the dbcan2 annotations are listed. * indicates the genes that were removed from the plot in Fig. 3.

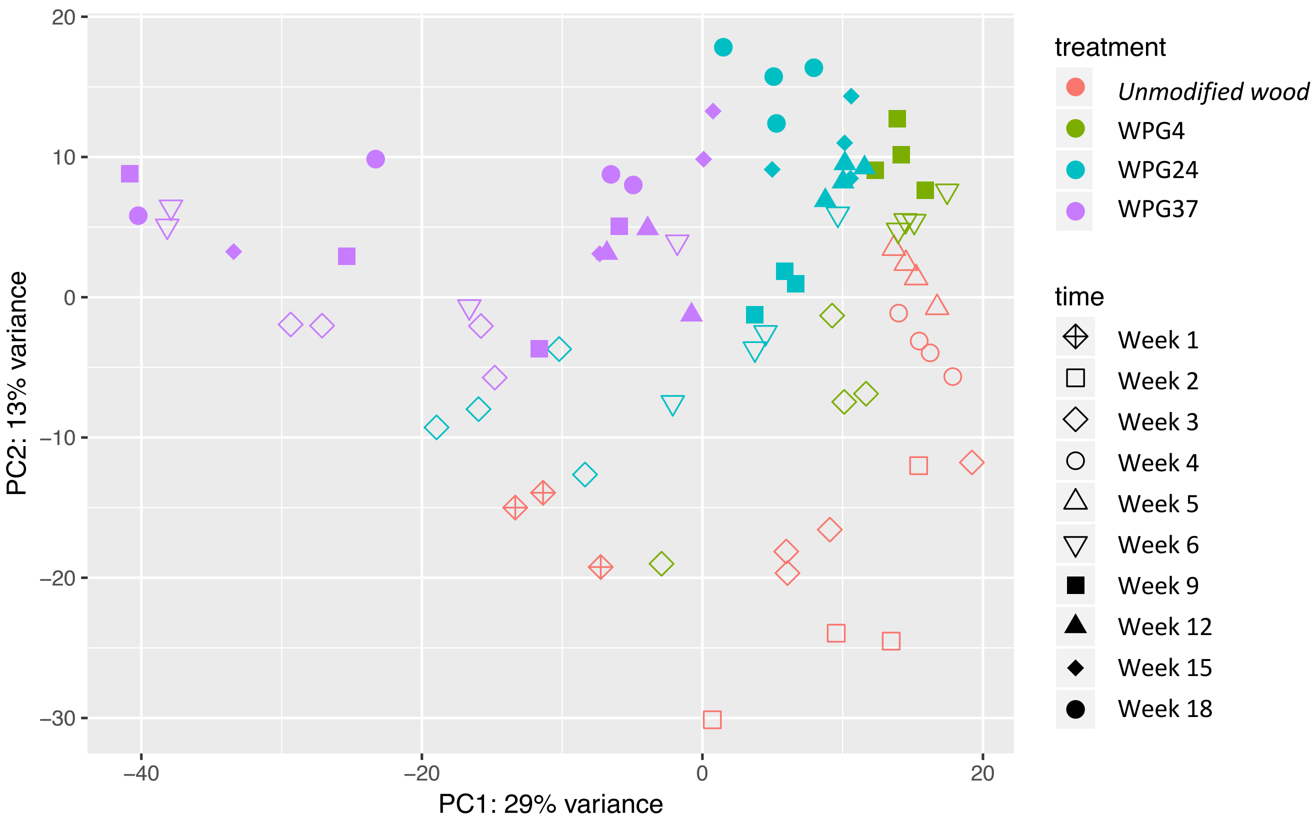

Figure S3. The figures of PCA plots of all the RNAseq replicates of *Rhodonia placenta* grown on unmodified radiata pine (*Pinus radiata*) and furfurylated radiata pine. In total 42% of the variance is explained by the two PCA axis.

Supplementary Figure 4

Glyoxylate dehydrogenase (GlyD) TRINITY_DN33196_c1_g1, Ppl 121561:

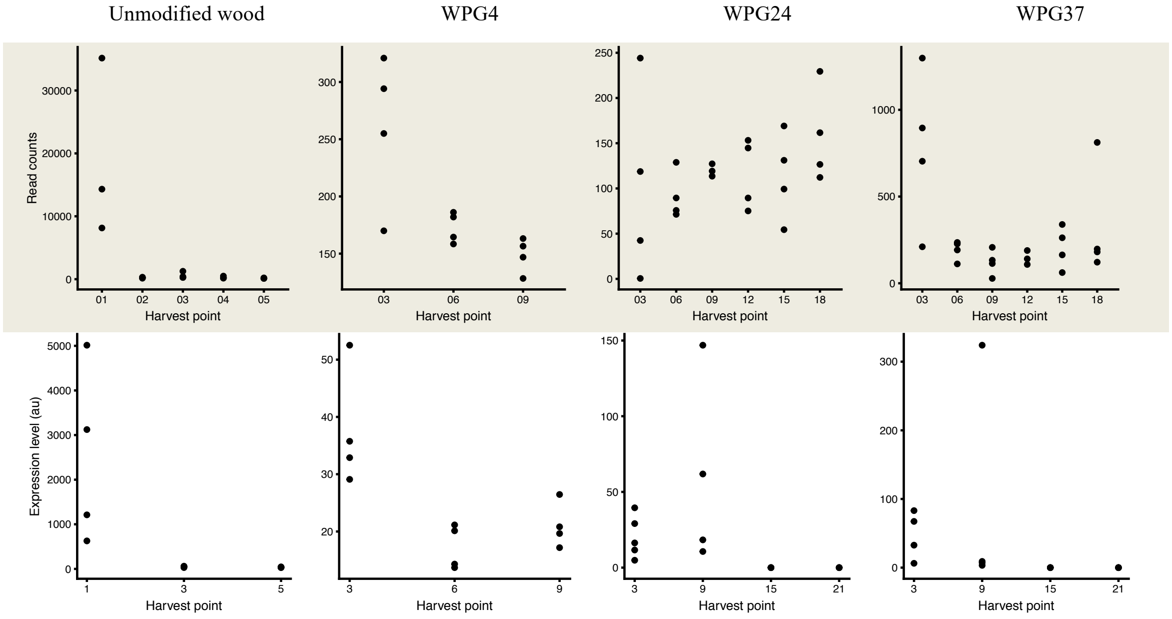

↑w1 ↑w3 NS NS

Between treatments: ↑*P. rad* vs. WPG4, WPG24, WPG37

Oxaloacetate acetylhydrolase (OahA) TRINITY_DN26529_c1_g1, Ppl112832

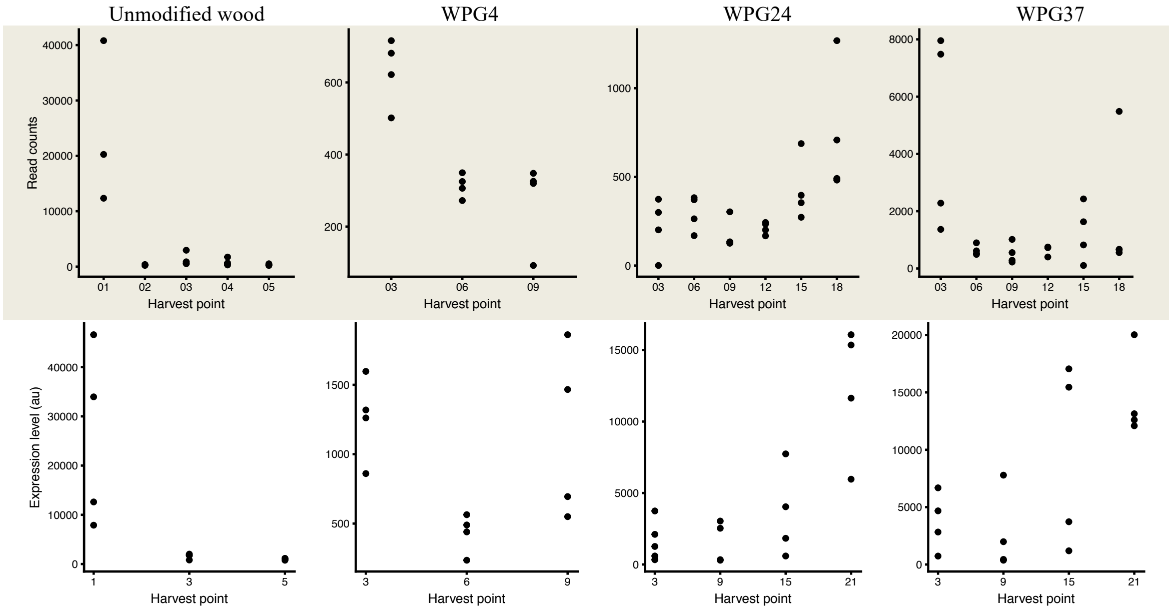

↑w1 ↑w3 vs. w6 ↑w21 ↑w21 vs. w3, w9

Between treatments: NS

Oxalate decarboxylase (OxaD) TRINITY_DN21938_c3_g2, Ppl43912:

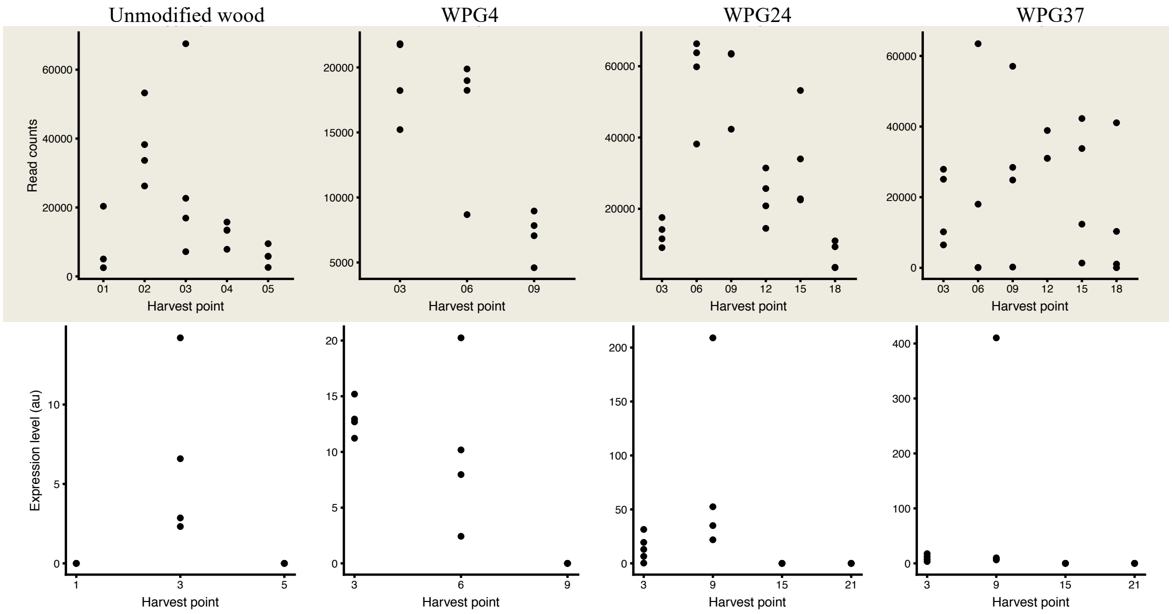

NS NS NS NS

Between treatments: NS

Supplementary Figure 4. The selected specific genes related to oxalic acid synthesis and decomposition. Shaded plots are based on RNAseq read counts, normalized on sequence library, non-shaded plots are based on qRT-PCR. For the qRT-PCR Tukey HSD are provided within treatments and between treatments (bottom left).

Supplementary Figure 5

AA3 GMC oxidoreductase (AOx1) TRINITY_DN18773_c0_g1, Ppl44331:

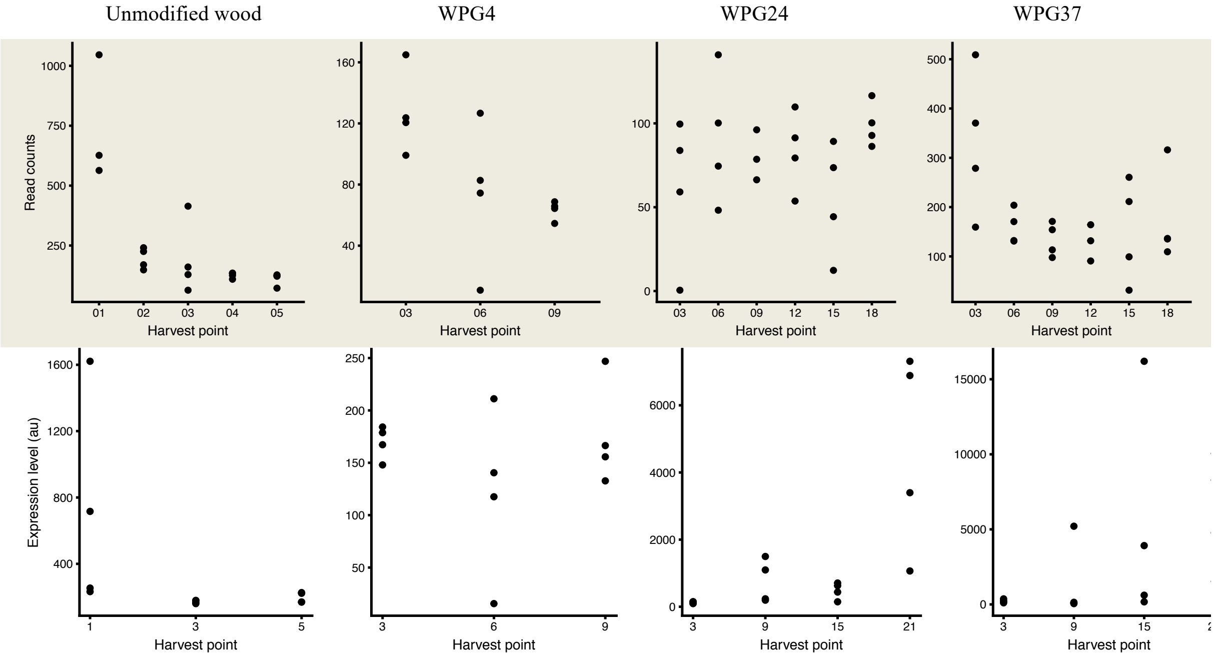

NS NS ↑w21 NS

Between treatments: ↑WPG37 vs. *P. rad.*, WPG4

AA3 GMC oxidoreductase (AOx2) TRINITY_DN20417_c3_g1, Ppl129158:

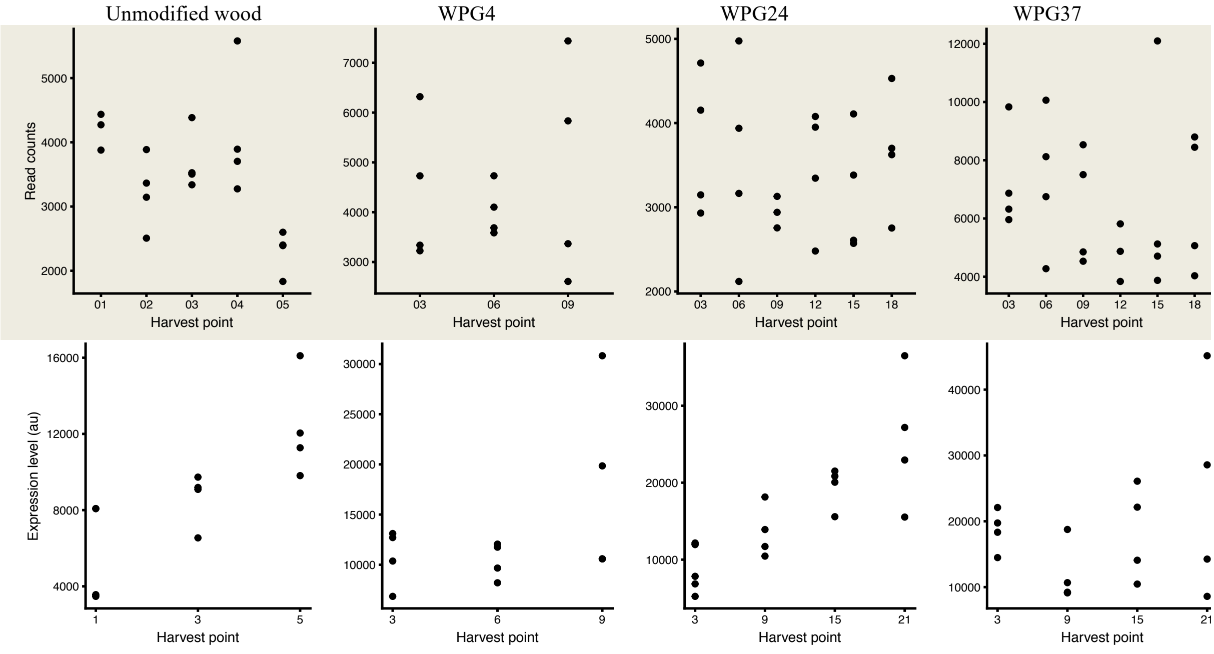

↑w5 vs. w1 NS ↑w21 vs. w3, w9 NS

Between treatments: ↑WPG24, WPG37 vs. *P. rad.*

AA3 GMC oxidoreductase (AOx3) TRINITY_DN28649_c4_g1, Ppl118723:

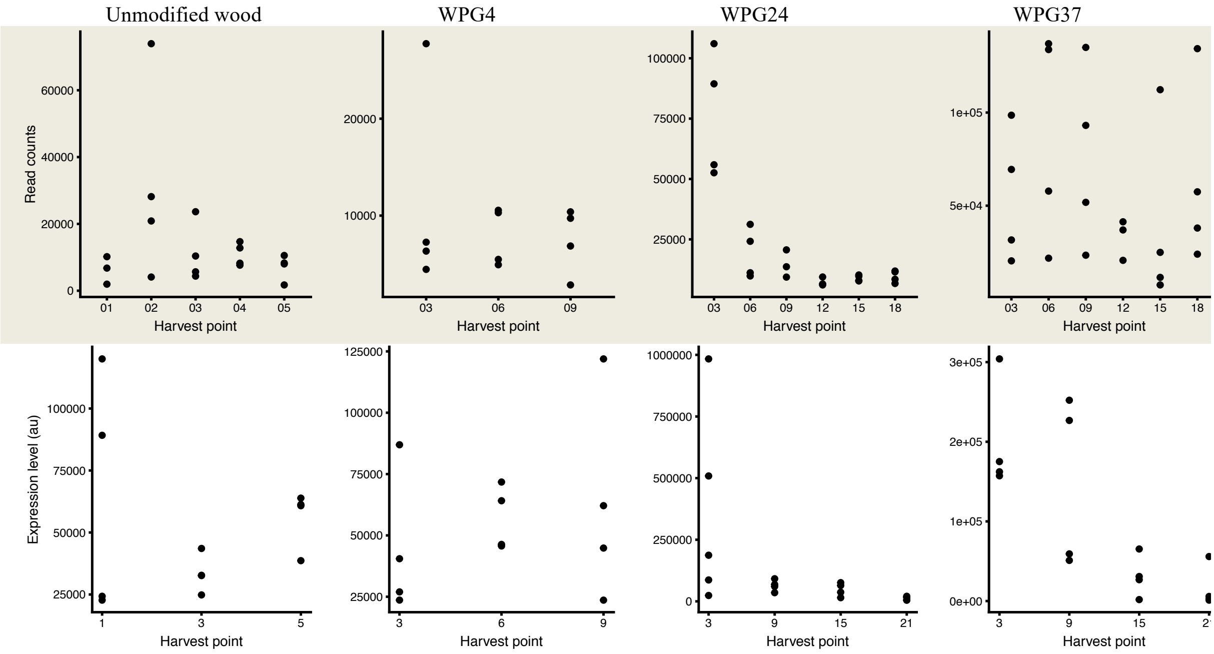

NS NS ↑w3 ↑w3 vs. w15,w21

Between treatments: NS

AA3 GMC oxidoreductase (AOx4) TRINITY_DN21062_c1_g1, Ppl55972

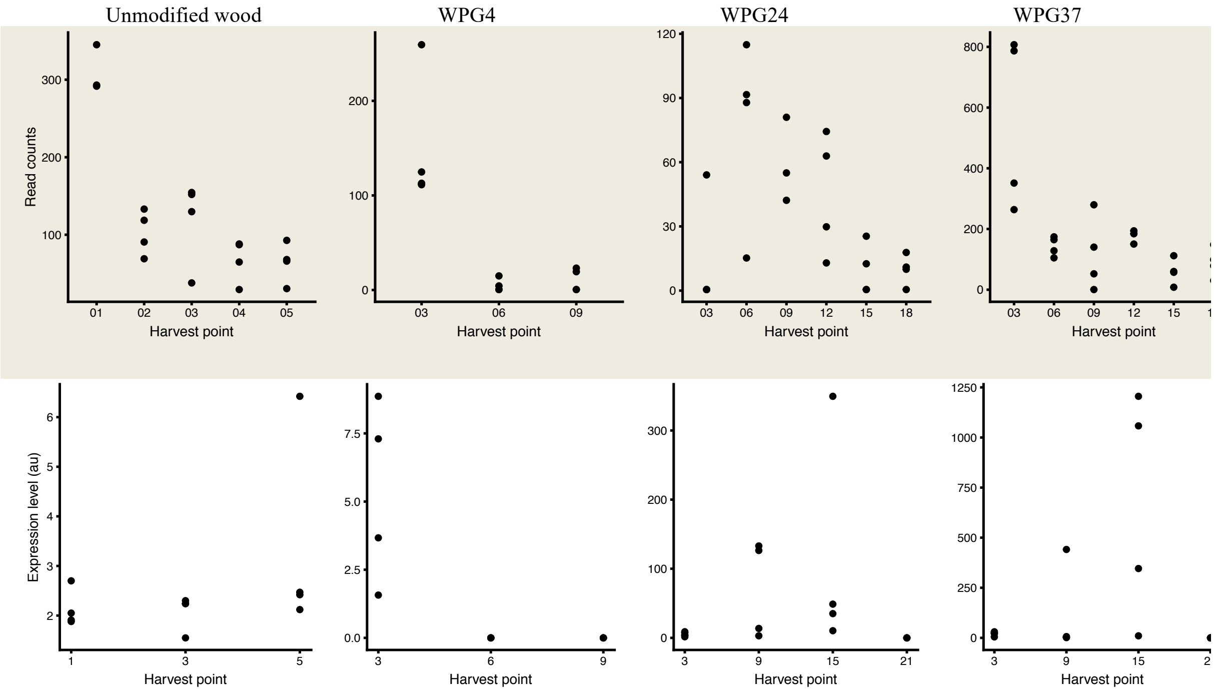

NS NS NS NS

Between treatments: NS

Similar mass loss: ↑WPG37

AA5 Copper radical oxidase (Cro1) TRINITY_DN21070_c1_g1, Ppl56703:

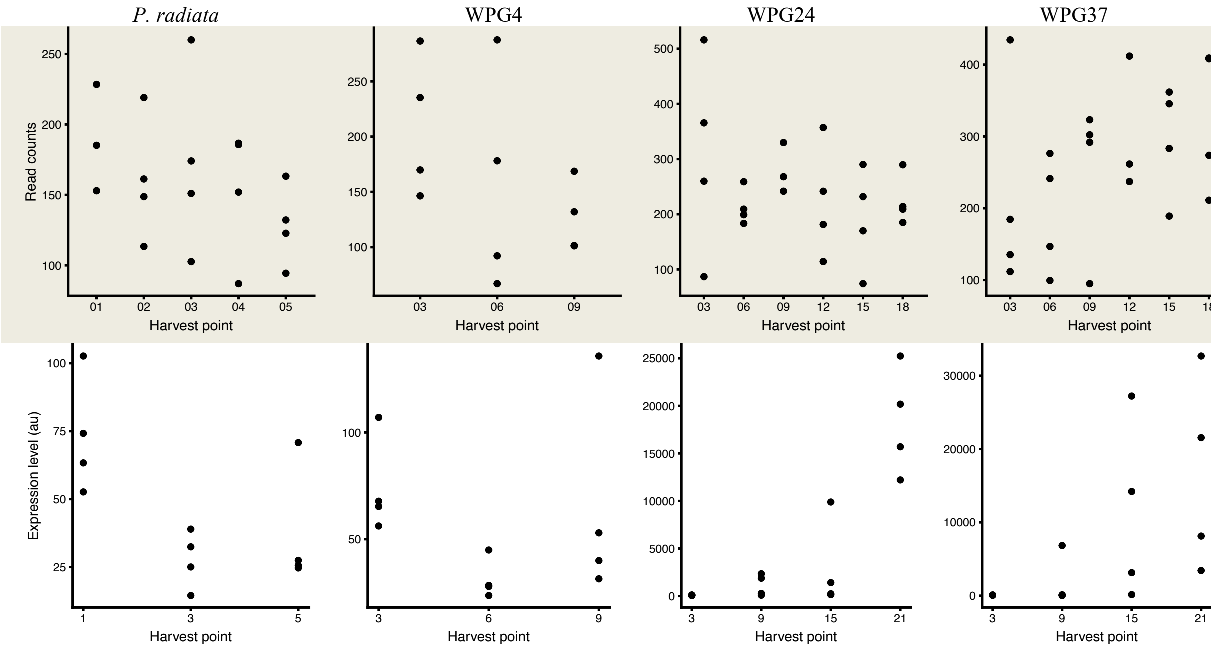

↑w1 vs. w3 NS ↑w21 NS

Between treatments: NS

AA5 Copper radical oxidase (Cro2) TRINITY_DN9270_c1_g1, Ppl104114:

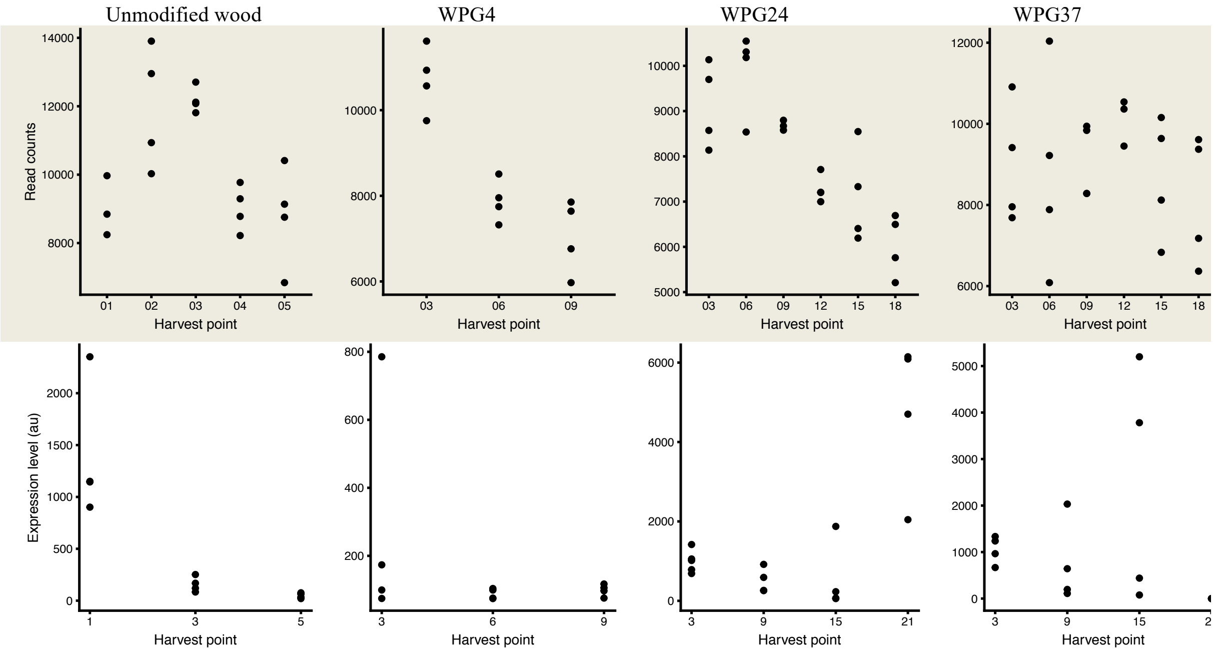

↑w1 NS ↑w21 ↑w3,w15

Between treatments: ↑WPG24 vs. WPG4

AA6 Benzoquinone reductase (BqR) TRINITY_DN21924_c2_g1, Ppl124517:

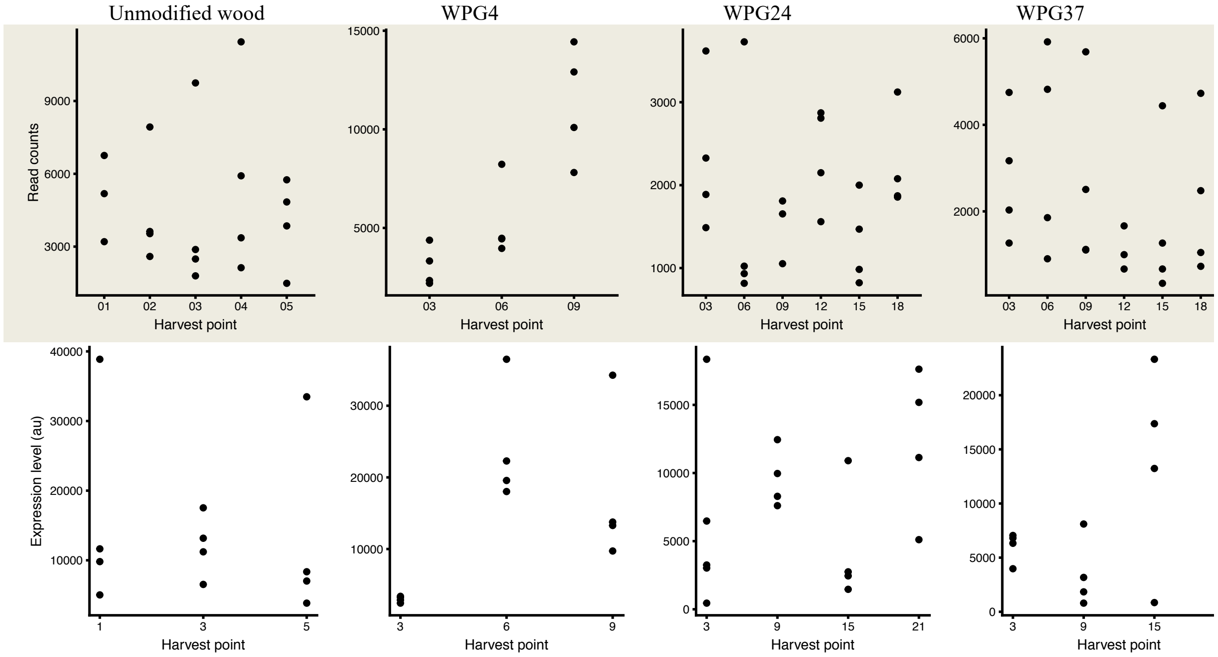

↑w6 vs. w3 | NS | NS | ↑w21 vs. w9

Between treatments: NS

Supplementary Figure 5. The genes related to oxidative decomposition. Shaded plots are based on RNAseq read counts, normalized on sequence library, non-shaded plots are based on qRT-PCR. For the qRT-PCR Tukey HSD are provided within treatments and between treatments (bottom left).

Supplementary Figure 6

GH5 Endomannanase (Man5a) TRINITY_DN30802_c4_g1, Ppl121831:

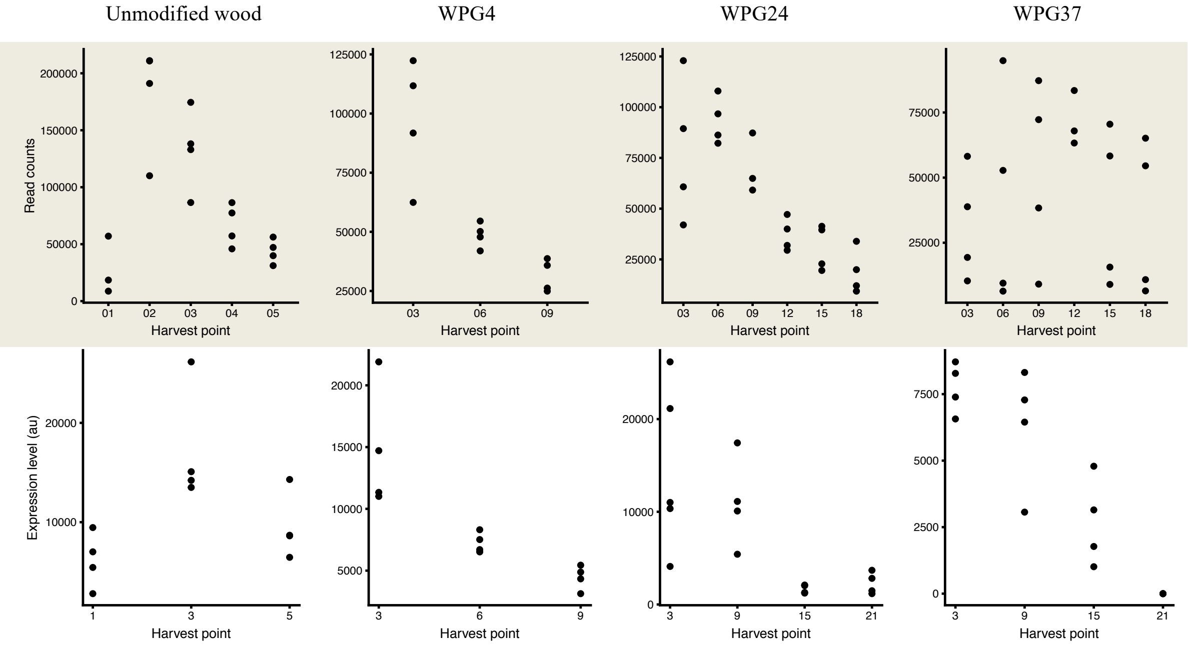

↑w3 vs. w1 ↑w3 ↑w3 vs. w15,w21 ↑w3,w9 vs. w15, w21

Between treatments: ↑*P. rad.* vs. WPG37

GH10 Endoxylanase (Xyl10a) TRINITY_DN11072_c0_g2, Ppl113670:

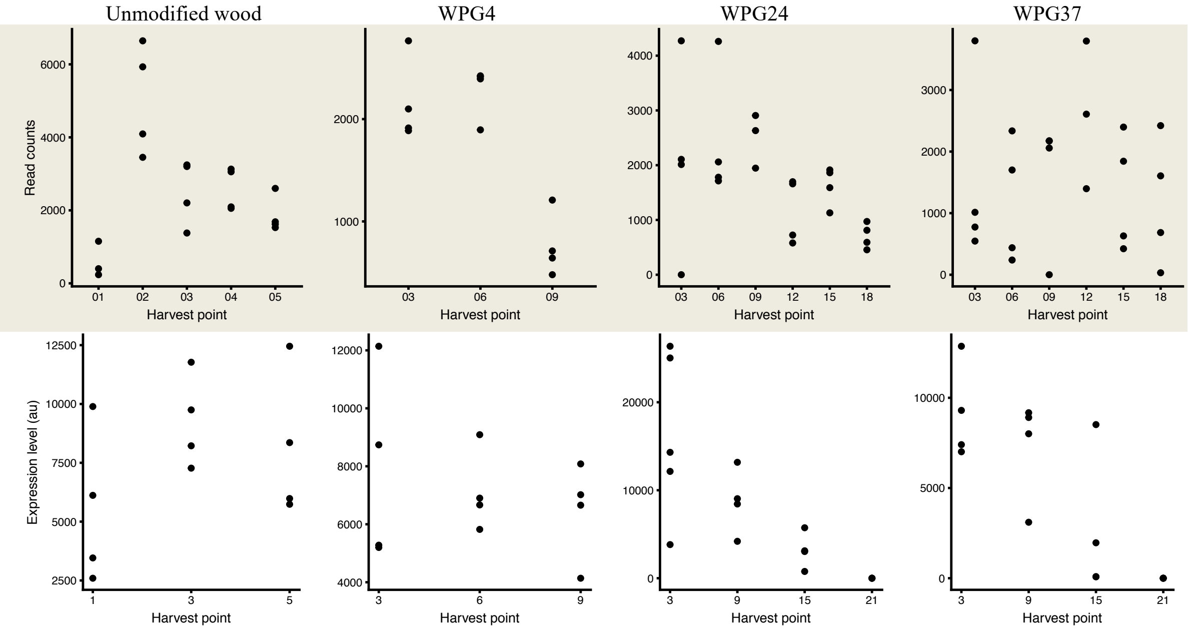

NS NS ↑w3 vs. w15,w21 ↑w3 vs. w15,w21

Between treatments: NS

GH10b Endoxylanase (Xyl10b) TRINITY_DN17151_c1_g1, Ppl105534:

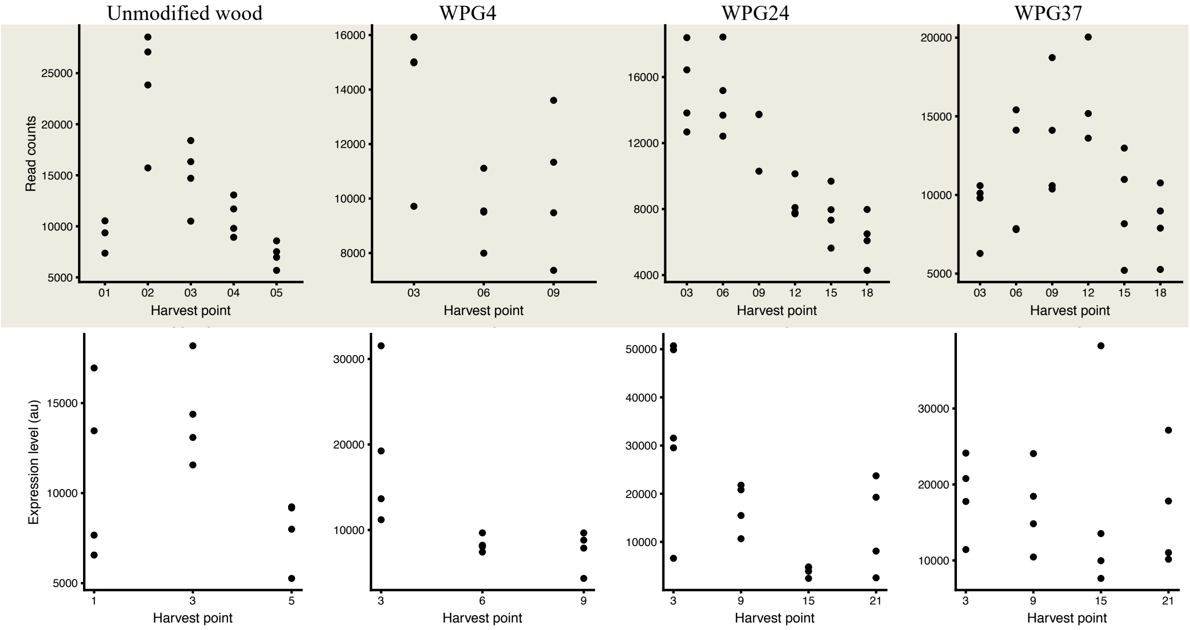

NS ↑w3 vs. w9 ↑w3 vs. w15 NS

Between treatments: NS

GH3 Beta xylosidase(bXyl) TRINITY_DN28569_c4_g1, Ppl51213 :

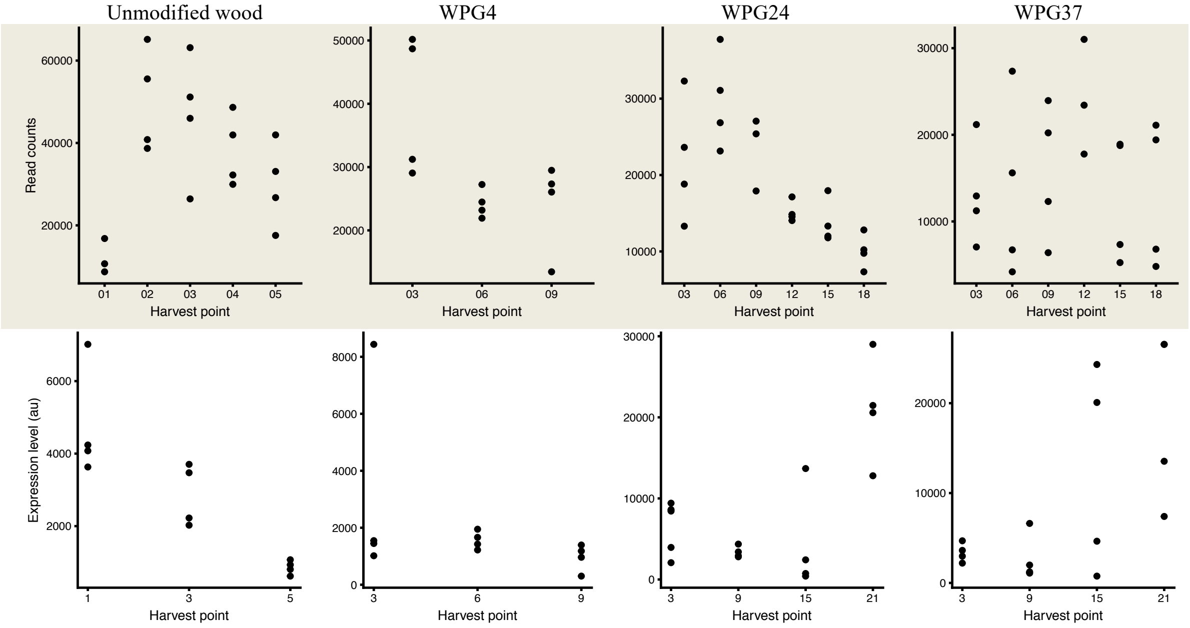

↑w3 vs. w5 NS ↑w21 ↑W21 vs. w3,w9

Between treatments: ↑WPG37 vs. WPG4

CE16 Carbohydrate esterase(CE16a) TRINITY_DN26470_c5_g1, Ppl125801:

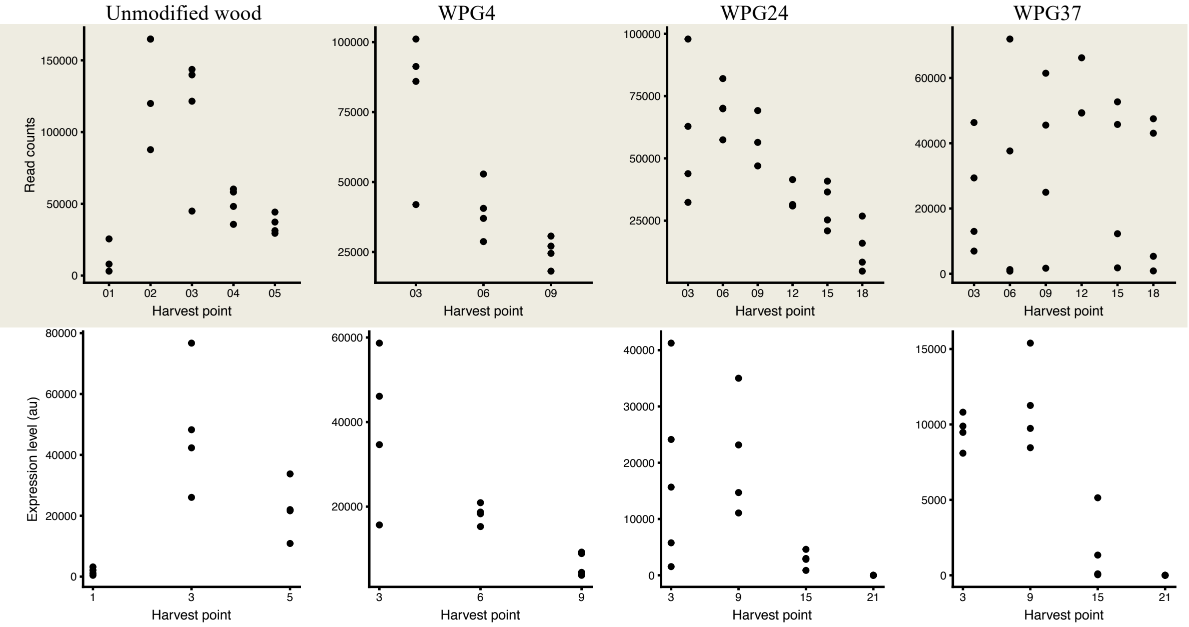

↑ w3 | ↑ w3 vs. w9 | NS | ↑w3,w6

Between treatments: ↑*P. rad*., WPG4 vs. WPG37

CE16 Carbohydrate esterase family 16 (CE16b) TRINITY_DN21066_c2_g6, Ppl48548

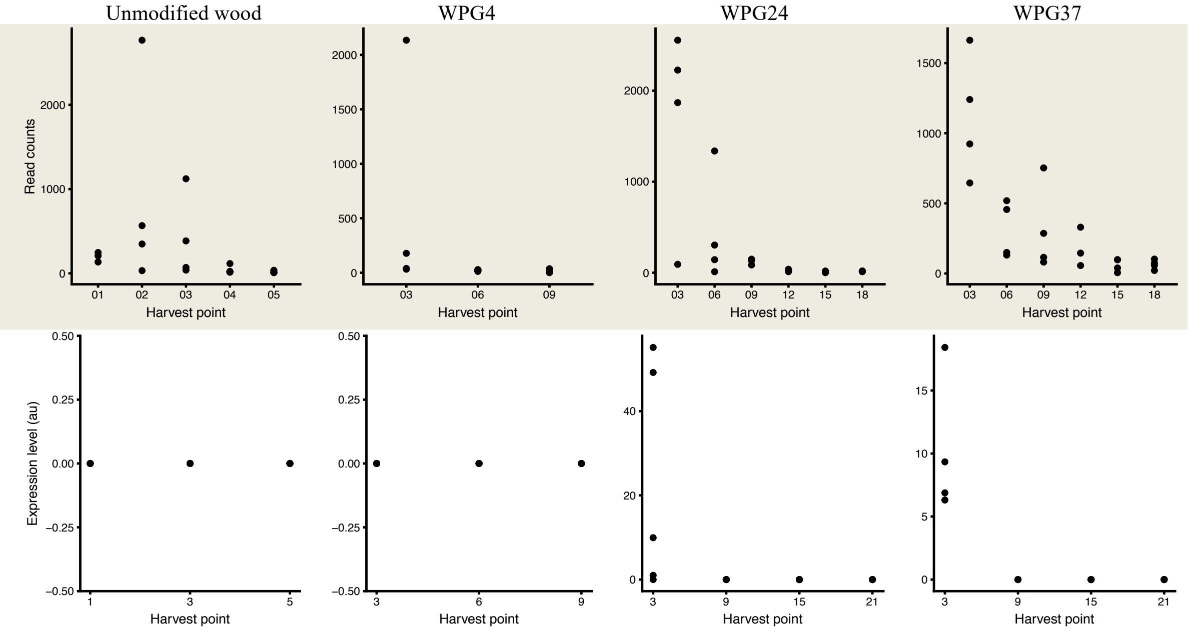

NS | NS | NS | NS

Between treatments: NS

GH28 Polygalacturonase (Gal28a) TRINITY_DN7127_c0_g2, Ppl111730:

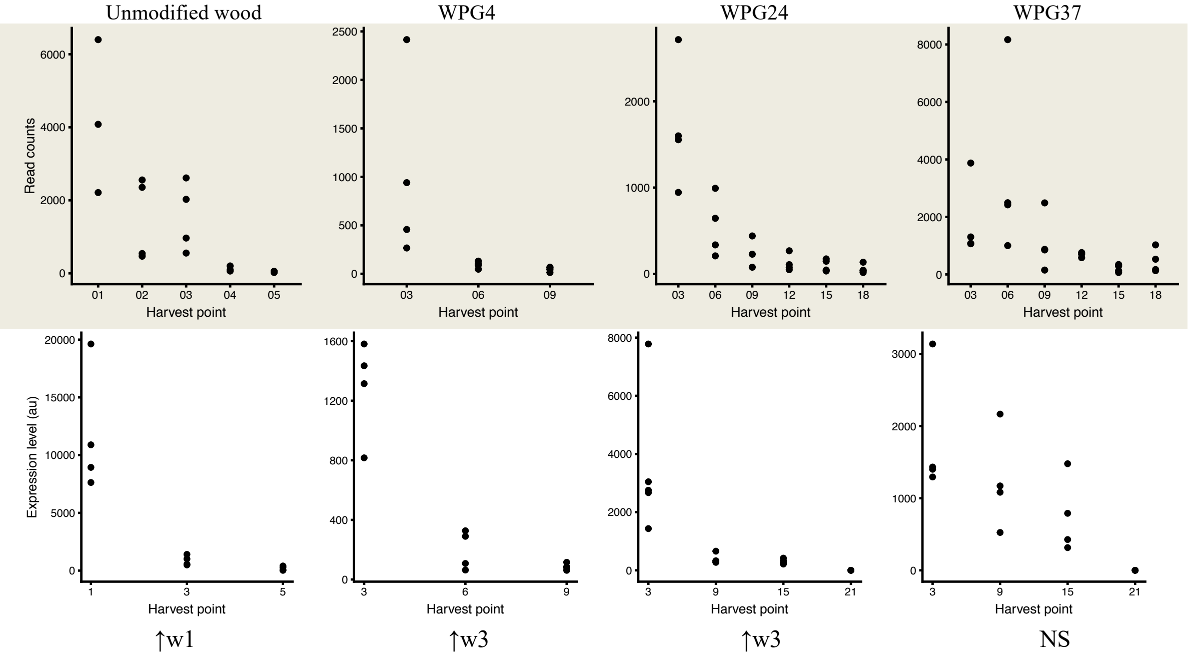

Between treatments: ↑*P. rad.* vs. WPG4, WPG37

Supplementary Figure 6. Genes related to hemicellulose and pectin degradation. Shaded plots are based on RNAseq read counts, normalized on sequence library, non-shaded plots are based on qRT-PCR. For the qRT-PCR Tukey HSD are provided within treatments and between treatments (bottom left).

Supplementary Figure 7

GH5 Endoglucanase (Cel5a) TRINITY_DN26393_c3_g1_i1, Ppl 115648:

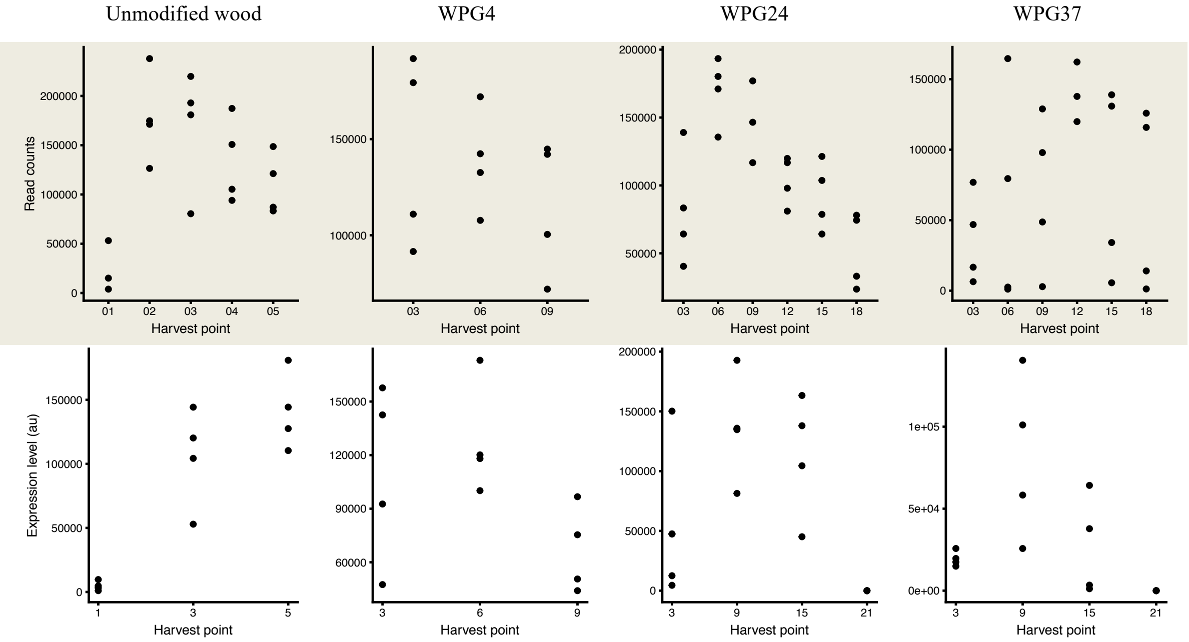

↑w5 vs. w1 NS ↑w9 vs. w21 ↑w9 vs. w3, w21

Between treatments: ↑WPG4 vs. WPG37

GH5 Endoglucanase (Cel5b) TRINITY_DN21725_c8_g1, Ppl103675:

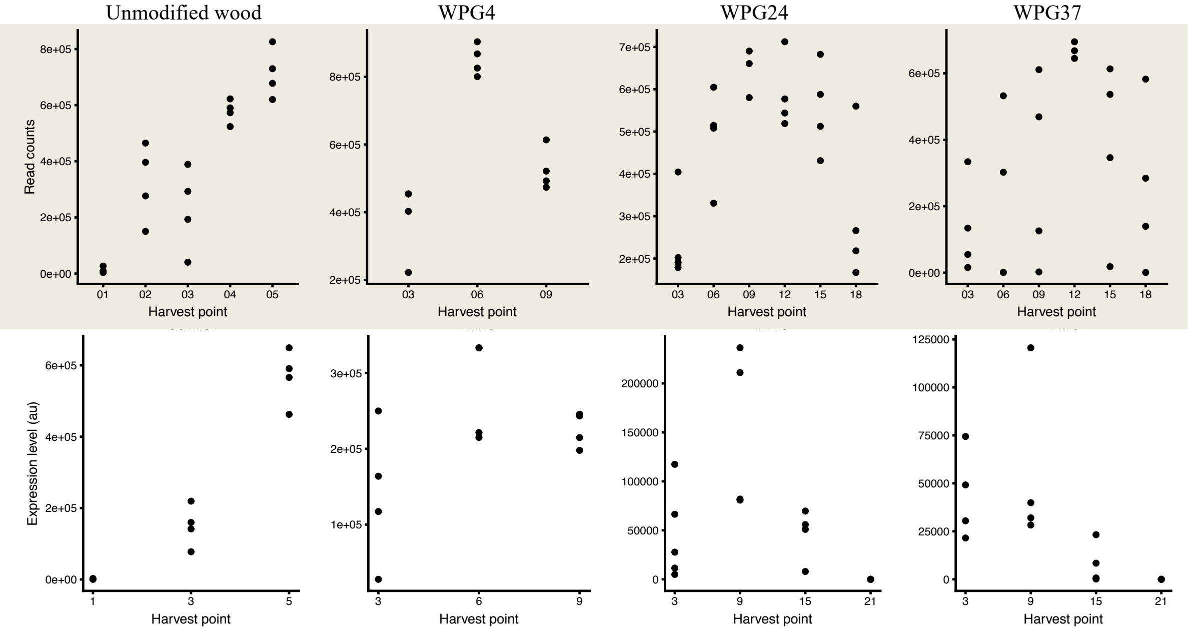

↑w5, w3 vs. w1 ↑w6 vs. w1 NS NS

Between treatments: ↑*P. rad.*, WPG4 vs. WPG24, WPG37

GH12 Glucoside hydrolase (Cel12A) TRINITY_DN33048_c6_g2, Ppl121191:

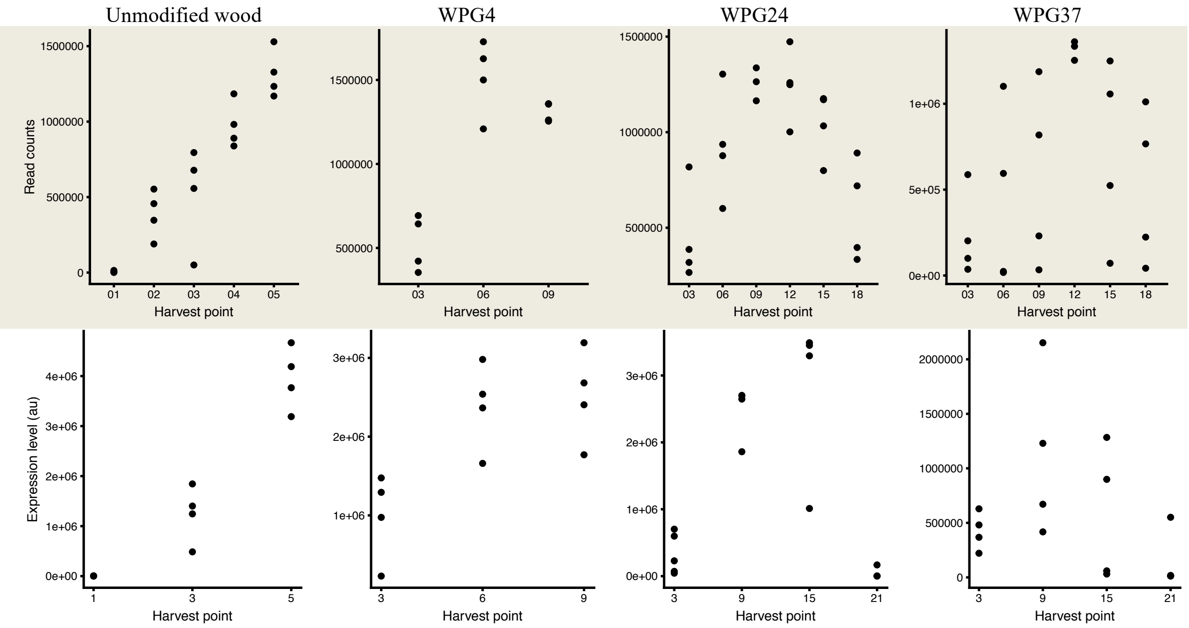

↑w5 ↓w3 ↑w9, w15 NS

Between treatments: ↑WPG4 vs. WPG37

GH3 Betaglucosidase (bGlu) TRINITY_DN21749_c2_g1, Ppl128500:

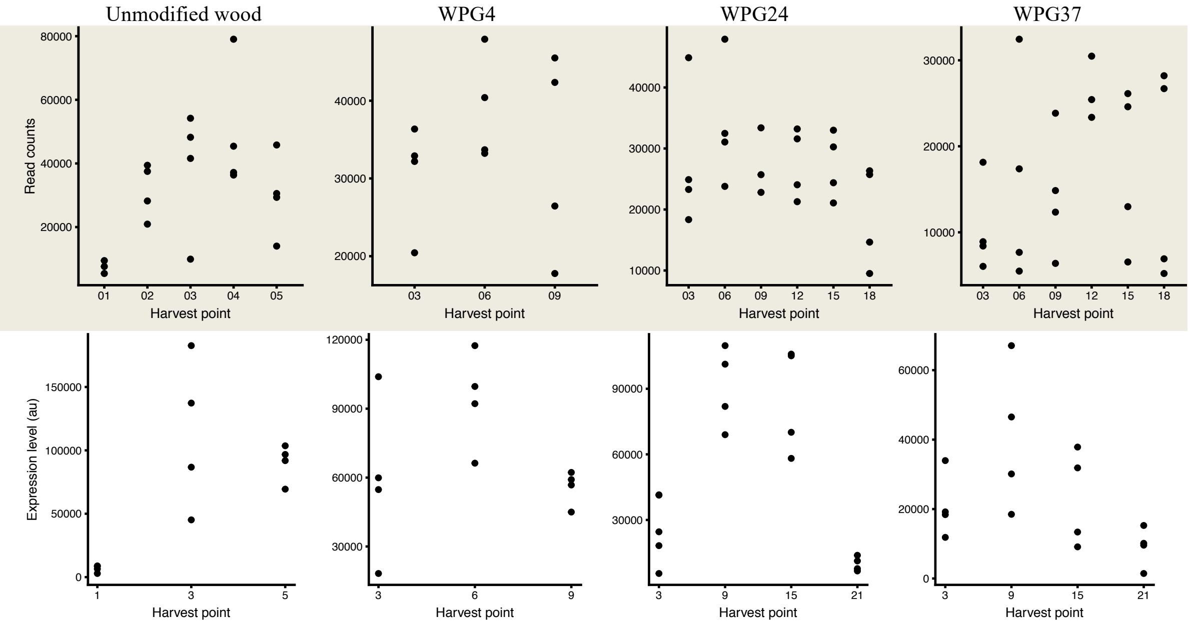

↑w3 vs. w1 NS ↑w9, w15 ↑w9 vs. w21

Between treatments: ↑*P. rad.*,WPG4 vs. WPG37.

AA9 Lytic polysaccharide monooxygenases (LPMO) TRINITY_DN16131_c0_g1, Ppl126811:

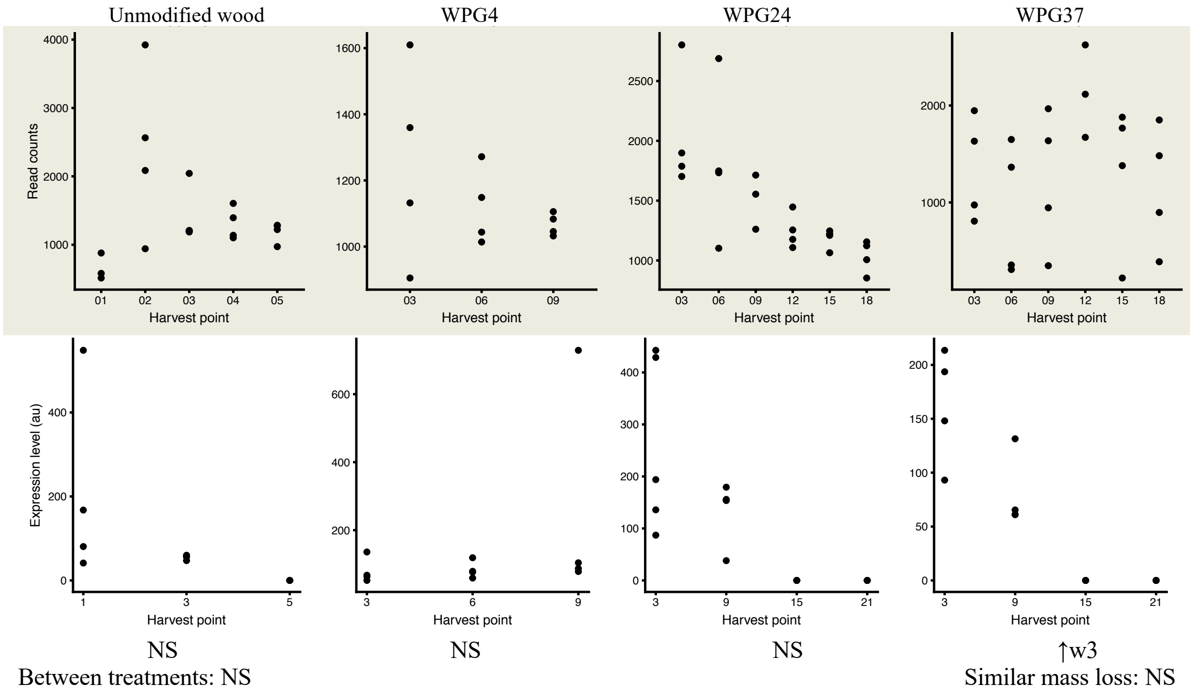

Supplementary Figure 7. The genes related to cellulose degradation. Shaded plots are based on RNAseq read counts, normalized on sequence library, non-shaded plots are based on qRT-PCR. For the qRT-PCR Tukey HSD are provided within treatments and between treatments (bottom left).

Supplementary Figure 8

Expansin (Exp1) TRINITY_DN6700_c0_g2, Ppl126976:

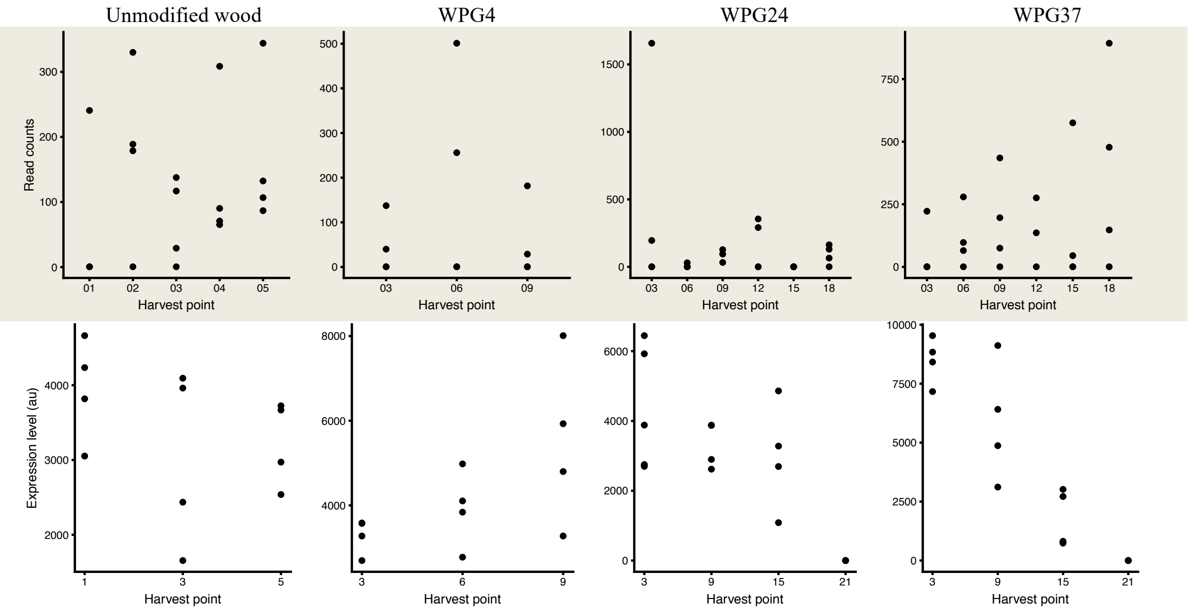

NS | NS | ↑w3, w9, w15 vs.w21 | ↑w3, w9

Between treatments: NS

Expansin (Exp2) TRINITY_DN24238_c3_g1, Ppl128179:

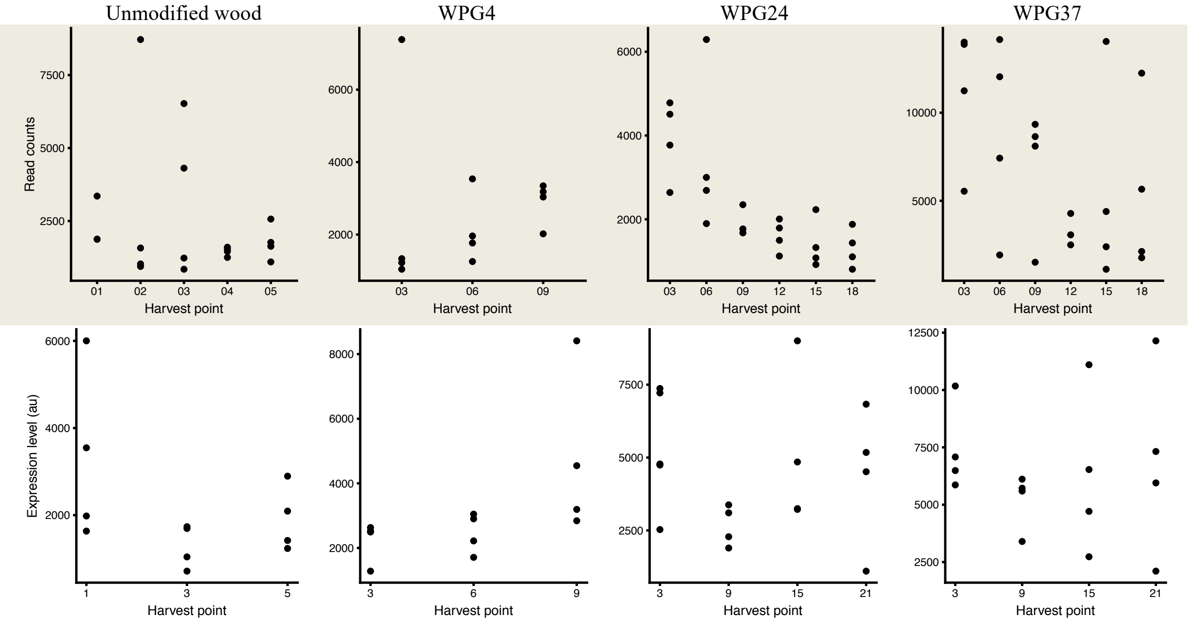

NS | NS | NS | NS

Between treatments: ↑WPG37 vs. *P. rad.*, WPG4

Supplementary Figure 8. Genes most likely involved in increasing enzyme accessibility. Shaded plots are based on RNAseq read counts, normalized on sequence library, non-shaded plots are based on qRT-

PCR. For the qRT-PCR Tukey HSD are provided within treatments and between treatments (bottom left).

Supplementary table 1. Results from Trinity_stats.pl with overall assembly statistics of the transcriptome of *Rhodonia placenta* strain FPRL 280

**Overall counts:**	
Total trinity contigs	56 520
Total trinity transcripts	114 539
Percent GC	54.04
**Stats based on ALL transcript contigs:**	
Contig N10	5 767
Contig N20	4 492
Contig N30	3 709
Contig N40	3 124
Contig N50	2 673
Median contig length	1 135
Average contig	1 612.38
Total assembled bases	184680754
**Stats based on ONLY LONGEST ISOFORM per 'GENE':**	
Contig N10	5 009
Contig N20	3 862
Contig N30	3 115
Contig N40	2 517
Contig N50	2 028
Median contig length	437
Average contig	1 006.22
Total assembled bases	56871752

Supplementary table 2. Number and percentage of conserved genes in BUSCO found in the transcriptome of *Rhodonia placenta* strain FPRL 280

Complete BUSCOs (C)	288 (99.3%)
Complete and single-copy BUSCOs (S)	172 (59.3 %)
Complete and duplicated BUSCOs (D)	116 (40.0%)
Fragmented BUSCOs (F)	2 (0.7 %)
Missing BUSCOs (M)	0 (0.0 %)
Total BUSCO groups searched (n)	290

Supplementary table 3. Enrichment analyses of GO terms and PFAM domains of significant differential expressed genes between clusters of similar expression patterns. Description indicates gene ontology variables; MF – Molecular function, BP – Biological process

Treatment	Cluster	GO terms and PFAM domains	Frequency	Adjusted p-value	Description	Comment
unmodified wood	K1	GO:0003735	159/281 (56.58%)	5.30E-05	MF	Structural constituent of ribosome
		GO:0006412	153/270 (56.67%)	8.23E-05	BP	Translation
		GO:0019843	26/30 (86.67%)	2.33E-04	MF	rRNA binding
		GO:0046686	15/16 (93.75%)	1.15E-02	BP	Response to cadmium ion
		**PF00009.22**	35/49 (71.43%)	2.49E-03	PFAM	GTP-binding elongation factor family, EF-Tu/EF-1A subfamily
	K2	GO:0005975	30/233 (12.88%)	5.16E-06	BP	Carbohydrate metabolic process
		GO:0004650	7/13 (53.85%)	1.36E-04	MF	Polygalacturonase activity
		GO:0004553	19/142 (13.38%)	1.79E-03	MF	Hydrolase activity, hydrolyzing O-glycosyl compounds
		**PF00295.12**	7/10 (70.00%)	3.30E-06	PFAM	GH28
		**PF04616.9**	3/5 (60.00%)	2.40E-01	PFAM	GH43
	K3	GO:0006355	25/275 (9.09%)	2.76E-02	BP	Regulation of transcription, DNA-templated
		**PF13417.1**	8/24 (33.33%)	2.36E-03	PFAM	Glutathione S-transferase
		**PF01036.13**	5/10 (50.00%)	1.77E-02	PFAM	Bacterial rhodopsins
	K4	No enrichment				
	K5	GO:0004553	34/142 (23.94%)	1.53E-07	MF	Hydrolase activity, hydrolyzing O-glycosyl compounds
		GO:0005975	39/233 (16.74%)	3.18E-04	BP	Carbohydrate metabolic process
		**PF14310.1**	6/7 (85.71%)	6.88E-04	PFAM	Fibronectin type III-like domain
		**PF01915.17**	7/13 (53.85%)	8.85E-03	PFAM	GH3
		**PF00933.16**	6/10 (60.00%)	1.70E-02	PFAM	GH3
	K6	No enrichment				
	K7	No enrichment				
	K8	GO:0005515	312/1361 (22.92%)	6.46E-08	MF	Protein binding
		**PF00628.24**	15/25 (60.00%)	2.53E-03	PFAM	PHD finger
		**PF00651.26**	36/95 (37.89%)	2.77E-03	PFAM	BTB/POZ domain
	K9	GO:0046872	37/525 (7.05%)	1.41E-05	MF	Metal ion binding
		GO:0000272	4/8 (50.00%)	2.09E-02	BP	
		**PF00096.21**	27/163 (16.56%)	2.77E-13	PFAM	Zinc finger
		**PF13894.1**	14/81 (17.28%)	2.56E-06	PFAM	C2H2-type zinc finger
	K10	GO:0008152	147/664 (22.14%)	1.89E-04	BP	Metabolic process

		GO:0006508	59/232 (25.43%)	1.45E-02	BP	Proteolysis
WPG4	K1	No enrichment				
	K2	No enrichment				
	K2	GO:0004553	21/142 (14.79%)	8.10E-11	MF	Hydrolase activity, hydrolyzing O-glycosyl compounds
		GO:0005975	25/233 (10.73%)	5.84E-10	BP	Carbohydrate metabolic process
		GO:0004650	5/13 (38.46%)	1.72E-03	MF	Polygalacturonase activity
		GO:0020037	21/419 (5.01%)	2.07E-02	MF	Heme binding
		GO:0016705	18/326 (5.52%)	2.28E-02	MF	Oxidoreductase activity, acting on paired donors, with incorporation or reduction of molecular oxygen
		**PF00295.12**	5/10 (50.00%)	5.71E-04	PFAM	GH28
		**PF00150.13**	6/25 (24.00%)	6.35E-03	PFAM	GH6
		**PF00067.17**	18/309 (5.83%)	4.82E-02	PFAM	CytP450
	K3	GO:0005515	465/1361 (34.17%)	7.49E-05	MF	Protein binding
		GO:0003824	137/367 (37.33%)	4.80E-02	MF	Catalytic activity
	K4	GO:0006078	3/5 (60.00%)	4.80E-02	BP	1->6)-beta-D-glucan biosynthetic process
	K5	GO:0005975	12/233 (5.15%)	3.22E-02	BP	Carbohydrate metabolic process
		**PF01425.16**	5/32 (15.62%)	4.65E-02	PFAM	Amidase
	K6	GO:0006412	198/270 (73.33%)	1.11E-05	BP	Translation
		GO:0003735	205/281 (72.95%)	1.25E-05	MF	Structural constituent of ribosome
		GO:0005524	769/1220 (63.03%)	2.74E-03	MF	ATP binding
	K7	**PF00755.15**	5/17 (29.41%)	1.12E-02	PFAM	Carnitine O-Palmitoyltransferase
	K8	No enrichment				
	K9	No enrichment				
	K10	No enrichment				
WPG24	K1	No enrichment				
	K2	No enrichment				
	K3	GO:0004553	41/142 (28.87%)	5.24E-07	MF	Hydrolase activity, hydrolyzing O-glycosyl compounds
		GO:0005975	56/233 (24.03%)	9.79E-07	BP	Carbohydrate metabolic process
		GO:0071555	11/24 (45.83%)	7.96E-03	BP	Cell wall organization
	K4	No enrichment				
	K5	GO:0003964	6/22 (27.27%)	1.65E-05	MF	RNA-directed DNA polymerase activity
		GO:0009036	5/19 (26.32%)	3.24E-04	MF	Type II site-specific deoxyribonuclease activity
		GO:0032199	5/19 (26.32%)	3.24E-04	BP	Reverse transcription involved in RNA-mediated transposition
		GO:0000737	5/20 (25.00%)	4.29E-04	BP	DNA catabolic process, endonucleolytic
		GO:0032197	5/21 (23.81%)	5.59E-04	BP	Transposition, RNA-mediated
		GO:0090305	5/25 (20.00%)	1.42E-03	BP	Nucleic acid phosphodiester bond hydrolysis
		GO:0006310	6/56 (10.71%)	5.68E-03	BP	DNA recombination
	K6	No enrichment				

	K7	No enrichment				
	K8	No enrichment				
	K9	GO:0005515	1132/1361 (83.17%)	1.08E-05	MF	Protein binding
		GO:0003676	310/358 (86.59%)	4.60E-03	MF	Nucleic acid binding
		GO:0016787	241/277 (87.00%)	2.40E-02	MF	Hydrolase activity
	K10	No enrichment				
WPG37	K1	GO:0003735	59/281 (21.00%)	3.96E-23	MF	Structural constituent of ribosome
		GO:0006412	55/270 (20.37%)	1.03E-20	BP	Translation
		GO:0019843	12/30 (40.00%)	7.64E-07	MF	rRNA binding
		GO:0009738	5/5 (100.00%)	9.23E-05	BP	Abscisic acid-activated signaling pathway
		GO:0009734	5/6 (83.33%)	5.35E-04	BP	Auxin-activated signaling pathway
		GO:0009651	7/15 (46.67%)	7.49E-04	BP	Response to salt stress
		GO:0009733	4/5 (80.00%)	1.08E-02	BP	Response to auxin
		GO:0046686	6/16 (37.50%)	2.13E-02	BP	Response to cadmium ion
		GO:0008422	4/6 (66.67%)	3.14E-02	MF	Kaempferol O-glucoside metabolic process
		GO:0009414	4/6 (66.67%)	3.14E-02	BP	Response to water deprivation
	K2	No enrichment				
	K3	No enrichment				
	K4	No enrichment				
	K5	No enrichment				
	K6	No enrichment				
	K7	No enrichment				
	K8	No enrichment				
	K9	No enrichment				
	K10	GO:0005515	1258/1361 (92.43%)	5.76E-08	MF	Protein binding
		GO:0055114	1077/1163 (92.61%)	4.90E-07	BP	Oxidation-reduction process
		GO:0016491	690/736 (93.75%)	1.96E-06	MF	Oxidoreductase activity
		GO:0008152	624/664 (93.98%)	3.56E-06	BP	Metabolic process
		GO:0043565	85/85 (100.00%)	7.11E-03	MF	Sequence-specific DNA binding
		GO:0003824	344/367 (93.73%)	2.53E-02	MF	Catalytic activity

Supplementary table 4. All genes were clustered into 10 clusters according to their similarity in expression patterns based on read counts from RNAseq data. Each treatment was analyzed separately. The table show how the genes suggested to be related to plant cell wall decay and selected for the qRT-PCR analyses are placed in these 10 clusters in order to visualize how specific functions are accumulating in some of the clusters.

Transcript ID	JGI Protein ID	Gene product (abbrevation)	unmodified wood	WPG4	WPG24	WPG37
TRINITY_DN33196_c1_g1	125161	Glyoxylate dehydrogenase (GlyD)	9	6	9	10
TRINITY_DN26529_c1_g1	112832	Oxaloacetate acetylhydrolase (OahA)	9	6	9	10
TRINITY_DN21938_c3_g2	43912	Oxalate decarboxylase (OxaD)	2	5	9	10
TRINITY_DN18773_c0_g1	44331	AA3 GMC oxidoreductase (AOx1)	4	6	9	10
TRINITY_DN20417_c3_g1	129158	AA3 GMC oxidoreductase (AOx2)	8	3	9	10
TRINITY_DN28649_c4_g1	118723	AA3 GMC oxidoreductase (AOx3)	10	6	3	10
TRINITY_DN21062_c1_g1	55972	AA3_3 Alcohol oxidase (AOx4)	4	7	9	10
TRINITY_DN21070_c1_g1	56073	AA5 Copper radical oxidase (Cro1)	8	6	9	10
TRINITY_DN9270_c1_g1	104114	AA5 Copper radical oxidase (Cro2)	10	6	9	10
TRINITY_DN21924_c2_g1	124517	AA6 Benzoquinone reductase (BqR)	1	8	9	10
TRINITY_DN26393_c3_g1_i1	115648	GH5 Endoglucanase (Cel5a)	5	6	9	10
TRINITY_DN21725_c8_g1	103675	GH5 Endoglucanase (Cel5b)	5	8	9	10
TRINITY_DN33048_c6_g2	121191	GH12 Glycoside hydrolase (Cel12A)	5	8	9	10
TRINITY_DN16131_c0_g1	126811	AA9 Lytic polysaccharide monooxygenase (LPMO)	10	6	9	10
TRINITY_DN21749_c2_g1	128500	GH3 Betaglucosidase (bGlu)	5	3	9	10
TRINITY_DN30802_c4_g1	121831	GH5 Endomannanse (Man5a)	2	2	9	10
TRINITY_DN11072_c0_g2	113670	GH10 Endoxylanase (Xyl10a)	5	5	9	10
TRINITY_DN17151_c1_g1	105534	GH10b Endoxylanase (Xyl10b)	10	6	9	10
TRINITY_DN28569_c4_g1	51213	GH3 Beta xylosidase (bXyl)	5	6	9	10
TRINITY_DN26470_c5_g1	125801	Carbohydrate esterase family 16 (CE16a)	5	2	9	10
TRINITY_DN21066_c2_g6	48548	Carbohydrate esterase family 16 (CE16b)	2	7	3	4
TRINITY_DN7127_c0_g2	111730	GH28 Polygalacturonase (Gal28a)	4	2	3	10
TRINITY_DN6700_c0_g2	126976	Potential expansin (Exp1)	8	3	9	10
TRINITY_DN24238_c3_g1	128179	Potential expansin (Exp2)	10	6	9	10

Supplementary table 5. Number of differential expressed genes among different harvest points of *Rhodonia placenta* growing on unmodified wood (*Pinus radiata*). **Bold** indicates up regulated in column week, and down regulated in row, *Italics* indicates the opposite

Treatment	Harvest point	Week 2	Week 3	Week 4	Week 5
unmodified wood	Week 1	2450 (**1119**/*1331*)	2074 (**1051**/*1023*)	2273 (**1237**/*1036*)	2238 (**1244**/*994*)
	Week 2	-	10(**10**)	279(**133**/*146*)	978(**475**/*503*)
	Week 3	-	-	50(**32**/*18*)	449(**149**/*300*)
	Week 4	-	-	-	131(**125**/*6*)

Supplementary table 6. Number of differential expressed genes among different harvest points of *Rhodonia placenta* growing on wood of radiata pine with three different levels of modification with furfuryl alcohol; i.e. Weigh Percent Gain (WPG) 4%, 24% and 37%. **Bold** indicates up regulated in column week, and down regulated in row, *Italics* indicates the opposite

Treatment	Harvest point	Week 6	Week 9	Week 12	Week 15	Week 18
WPG	Week 3	103(**36**/*67*)	481(**171**/*310*)	-	-	-
	Week 6	-	0	-	-	-
WPG24	Week 3	2738(**1311**/*1427*)	1320(**963**/*357*)	3979(**2182**/*1797*)	4766(**2353**/*2413*)	4213(**2533**/*1680*)
	Week 6	-	0	0	17(**9**/*8*)	773(**452**/*321*)
	Week 9	-	-	0	0	142(**72**/*70*)
	Week 12	-	-	-	0	35(**29**/*6*)
	Week 15	-	-	-	-	66(**61**/*7*)
WPG37	Week 3	15(**4**/*11*)	9(**4**/*5*)	57(**32**/*25*)	78(**29**/*49*)	140(**64**/*76*)
	Week 6	-	0	0	2(**1**/*1*)	73(**70**/*3*)
	Week 9	-	-	5(**0**/*5*)	0	4(**4**/*0*)
	Week 12	-	-	-	0	40(**2**/*38*)
	Week 15	-	-	-	-	5(**5**/*0*)

Supplementary table 7. Enrichment analyses of GO terms and PFAM domains of significant differential expressed genes between harvest points of *Rhodonia placenta* growing on unmodified wood (radiata pine) and radiata pine with three different levels of modification with furfuryl alcohol. All pairwise comparisons are within treatment between different harvest points. Down week 1 – week 2, indicates lower expression levels in week 1 compared to week 2, and Up week 1 – week 2, indicates higher expression levels in week 1 compared to week 2. Description indicates gene ontology variables; MF – Molecular function, BP – Biological process

Treatment	Contrasts between time	GO terms and PFAM domains	Frequency	Adjusted p-value	Description	Comment
unmodified wood	Down week 1 – Up week 2	GO:0004553	40/142 (28.17%)	3.17E-13	MF	hydrolase activity, hydrolyzing O-glycosyl compounds
		GO:0005975	49/233 (21.03%)	4.86E-11	BP	carbohydrate metabolic process
		GO:0008152	83/664 (12.50%)	1.26E-06	BP	metabolic process
		GO:0055114	117/1163 (10.06%)	1.98E-04	BP	oxidation-reduction process
		GO:0016787	39/277 (14.08%)	1.95E-03	MF	hydrolase activity
		GO:0003824	47/367 (12.81%)	2.79E-03	MF	catalytic activity
		GO:0016491	77/736 (10.46%)	7.40E-03	MF	oxidoreductase activity
	Up week 1 – Down week 2	GO:0046872	50/525 (9.52%)	3.78E-03	MF	metal ion binding
		**PF00096.21**	36/163 (22.09%)	5.02E-10		C2H2 type zinc finger
		**PF13894.1**	8/81 (22.22%)	2.78E-04		C2H2 type zinc finger
	Down week 1 – Up week 3	GO:0004553	34/142 (23.94%)	2.03E-10	MF	hydrolase activity, hydrolyzing O-glycosyl compounds
		GO:0005975	41/233 (17.60%)	3.33E-08	BP	carbohydrate metabolic process
		GO:0008152	73/664 (10.99%)	1.15E-05	BP	metabolic process
		GO:0016491	74/736 (10.05%)	3.56E-04	MF	oxidoreductase activity
		GO:0055114	104/1163 (8.94%)	4.74E-04	BP	oxidation-reduction process
		GO:0016787	35/277 (12.64%)	3.98E-03	MF	hydrolase activity
		**PF14310.1**	5/7 (71.43%)	1.37E-02		Fibronectin type III-like domain
		**PF01055.21**	5/8 (62.50%)	3.45E-02		GH31
	Up week 1 – Down week 3	GO:0005515	88/1361 (6.47%)	2.19E-04	MF	protein binding
		GO:0046872	41/525 (7.81%)	7.65E-03	MF	metal ion binding
		**PF00096.21**	35/163 (21.47%)	1.37E-12		C2H2 type zinc finger
		**PF13894.1**	16/81 (19.75%)	2.32E-04		C2H2 type zinc finger
		**PF00646.28**	18/136 (13.24%)	1.74E-02		F-box domain
	Down week 1 – Up week 4	GO:0008152	75/664 (11.30%)	4.37E-03	BP	metabolic process
		GO:0016491	81/736 (11.01%)	4.89E-03	MF	oxidoreductase activity
		GO:0004553	24/142 (16.90%)	1.99E-02	MF	hydrolase activity, hydrolyzing O-glycosyl compounds
		**PF07690.11**	36/222 (16.22%)	1.02E-02		major Facilitator Superfamily

		**PF08659.5**	26/142 (18.31%)	1.89E-02		KR domain
		**PF00106.20**	32/198 (16.16%)	3.23E-02		short chain dehydrogenase
	Up week 1 – Down week 4	GO:0005515	93/1361 (6.83%)	9.76E-03	MF	protein binding
		GO:0046872	45/525 (8.57%)	1.41E-02	MF	metal ion binding
		**PF00096.21**	30/163 (18.40%)	5.01E-07		C2H2 type zinc finger
		**PF13894.1**	14/81 (17.28%)	3.83E-02		C2H2 type zinc finger
	Down week 1 – Up week 5	GO:0008152	76/664 (11.45%)	1.21E-04	BP	metabolic process
		GO:0016491	80/736 (10.87%)	4.85E-04	MF	oxidoreductase activity
		GO:0055114	107/1163 (9.20%)	1.69E-02	BP	oxidation-reduction process
		GO:0016787	36/277 (13.00%)	1.84E-02	MF	hydrolase activity
		**PF07690.11**	33/222 (14.86%)	2.90E-02		Major Facilitator Superfamily
	Up week 1 – Down week 5	GO:0005515	94/1361 (6.91%)	4.66E-04	MF	protein binding
		**PF01794.14**	6/11 (54.55%)	3.27E-03		Ferric reductase like transmembrane component
		**PF00096.21**	22/163 (13.50%)	8.44E-03		C2H2 type zinc finger
		**PF08022.7**	6/14 (42.86%)	1.87E-02		FAD-binding domain
WPG4	Up week 3 – Down week 6	GO:0055114	16/1163 (1.38%)	8.68E-03	BP	oxidation-reduction process
		**PF07993.7**	4/44 (9.09%)	3.99E-02		male sterility protein
	Down week3 – Up week 6	No enrichment				
	Up week 3 – Down week 9	GO:004262	9/66 (13.64%)	3.59E-03	MF	ATPase activity, coupled to transmembrane movement of substances
		GO:0006508	16/232 (6.90%)	8.66E-03	BP	proteolysis
		GO:0055114	43/1163 (3.70%)	1.59E-02	BP	oxidation-reduction process
		GO:0005975	15/233 (6.44%)	3.66E-02	BP	carbohydrate metabolic process
		GO:0020037	21/419 (5.01%)	4.89E-02	MF	heme binding
		**PF00664.18**	7/42 (16.67%)	2.35E-02		ABC transporter transmembrane region
		**PF00295.12**	4/10 (40.00%)	3.09E-02		GH28
	Down week 3 – Up week 9	No enrichment				
WPG24	Up week 3 – Down week 6	GO:0004553	21/142 (14.79%)	1.98E-03	MF	hydrolase activity, hydrolyzing O-glycosyl compounds
		**PF07716.10**	6/16 (37.50%)	4.65E-02		basic region leucine zipper
	Down week 3 – Up week 6	No enrichment				
		**PF03144.20**	8/22 (36.36%)	2.19E-02		elongation factor Tu domain 2
	Up week 3 – Down week 9	No enrichment				
	Down week 3 – Up week 9	No enrichment				
	Up week 3 – Down week 12	GO:0005975	47/233 (20.17%)	9.53E-10	BP	carbohydrate metabolic process
		GO:0004553	35/142 (24.65%)	1.96E-09	MF	hydrolase activity, hydrolyzing O-glycosyl compounds
		GO:0004650	8/13 (61.54%)	2.08E-04	MF	polygalacturonase activity
		GO:0016491	79/736 (10.73%)	2.53E-03	MF	oxidoreductase activity
		GO:0004190	18/87 (20.69%)	6.34E-03	MF	aspartic-type endopeptidase activity

	GO:0055114	111/1163 (9.54%)	6.86E-03	BP	oxidation-reduction process
	GO:0004499	9/26 (34.62%)	1.59E-02	MF	N,N-dimethylaniline monooxygenase activity
	**PF00295.12**	8/10 (80.00%)	1.29E-05		GH28
	**PF00108.18**	6/10 (60.00%)	1.15E-02		thiolase, N-terminal domain
	**PF00150.13**	9/25 (36.00%)	1.65E-02		GH5
	**PF00026.18**	17/81 (20.99%)	1.88E-02		eukaryotic aspartyl protease
	**PF00743.14**	9/26 (34.62%)	2.37E-02		flavin-binding monooxygenase-like
Down week 3 – Up week 12	No enrichment				
	**PF01926.18**	23/85 (27.06%)	1.43E-02		50S ribosome-binding GTPase
Up week 3 – Down week 15	GO:0005975	60/233 (25.75%)	4.02E-12	BP	carbohydrate metabolic process
	GO:0004553	43/142 (30.28%)	7.76E-11	MF	hydrolase activity, hydrolyzing O-glycosyl compounds
	GO:0004650	9/13 (69.23%)	1.08E-04	MF	polygalacturonase activity
	GO:0016491	104/736 (14.13%)	2.04E-04	MF	oxidoreductase activity
	GO:0055114	142/1163 (12.21%)	7.25E-03	BP	oxidation-reduction process
	GO:0004190	21/87 (24.14%)	9.42E-03	MF	aspartic-type endopeptidase activity
	**PF00295.12**	9/10 (90.00%)	3.02E-06		GH28
	**PF00026.18**	20/81 (24.69%)	2.35E-02		Eukaryotic aspartyl protease
	**PF00150.13**	10/25 (40.00%)	2.69E-02		GH5
Down week 3 – Up week 15	GO:0005515	178/1361 (13.08%)	9.27E-03	MF	protein binding
	GO:0004812	16/55 (29.09%)	3.51E-02	MF	aminoacyl-tRNA ligase activity
	GO:0006418	15/50 (30.00%)	4.06E-02	BP	tRNA aminoacylation for protein translation
	**PF14226.1**	11/26 (42.31%)	3.36E-02		non-haem dioxygenase in morphine synthesis N-terminal
Up week 3 – Down week 18	GO:0004553	48/142 (33.80%)	4.93E-19	MF	hydrolase activity, hydrolyzing O-glycosyl compounds
	GO:0005975	61/233 (26.18%)	3.79E-18	BP	carbohydrate metabolic process
	GO:0016491	90/736 (12.23%)	9.85E-06	MF	oxidoreductase activity
	GO:0004650	9/13 (69.23%)	1.17E-05	MF	polygalacturonase activity
	GO:0006508	37/232 (15.95%)	5.68E-04	BP	proteolysis
	GO:0055114	120/1163 (10.32%)	5.68E-04	BP	oxidation-reduction process
	GO:0004190	20/87 (22.99%)	7.17E-04	MF	aspartic-type endopeptidase activity
	GO:0004499	10/26 (38.46%)	2.75E-03	MF	N,N-dimethylaniline monooxygenase activity
	GO:0006725	5/8 (62.50%)	4.95E-02	BP	cellular aromatic compound metabolic process
	GO:0008199	5/8 (62.50%)	4.95E-02	MF	ferric iron binding
	**PF00295.12**	9/10 (90.00%)	3.58E-07		GH28
	**PF00150.13**	12/25 (48.00%)	3.06E-05		GH5
	**PF00775.16**	5/5 (100.00%)	1.39E-03		dioxygenase
	**PF00026.18**	19/81 (23.46%)	2.75E-03		ukaryotic aspartyl protease
	**PF00743.14**	10/26 (38.46%)	4.98E-03		flavin-binding monooxygenase-like

		**PF01036.13**	6/10 (60.00%)	1.64E-02		bacteriorhodopsin-like protein
		**PF13738.1**	10/30 (33.33%)	2.16E-02		pyridine nucleotide-disulphide oxidoreductase
	Down week 3 – Up week 18	GO:0005515	203/1361 (14.92%)	2.08E-04	MF	protein binding
		**PF01926.18**	25/85 (29.41%)	1.35E-02		50S ribosome-binding GTPase
WPG37	Up week 3 – Down week 6	No enrichment				
	Down week 3 – Up week 6	No enrichment				
	Up week 3 – Down week 9	No enrichment				
	Down week 3 – Up week 9	No enrichment				
	Up week 3 – Down week 12	GO:0055114	10/1163 (0.86%)	1.28E-03	BP	oxidation-reduction process
		**PF07690.11**	5/222 (2.25%)	7.33E-03		major Facilitator Superfamily
	Down week 3 – Up week 12	No enrichment				
	Up week 3 – Down week 15	GO:0055114	15/1163 (1.29%)	2.87E-06	BP	oxidation-reduction process
	Down week 3 – Up week 15	No enrichment				
	Up week 3 – Down week 18	GO:0055114	14/1163 (1.20%)	7.83E-03	BP	oxidation-reduction process
		**PF00067.17**	8/309 (2.59%)	1.20E-02		cytochrome P450
	Down week 3 – Up week 18	GO:0016705	8/326 (2.45%)	7.29E-03	MF	oxidoreductase activity, acting on paired donors, with incorporation or reduction of molecular oxygen
		GO:0005506	8/367 (2.18%)	1.73E-02	MF	iron ion binding
		GO:0020037	8/419 (1.91%)	4.45E-02	MF	heme binding

Supplementary table 8. Enrichment analyses of GO terms and PFAM domains of significant differential expressed genes between pairwise comparisons within treatment between harvest points. Down W1 – UP WX, indicates lower expression levels in week 1 compared to week 2, and Up W1 – DOWN W2, indicates higher expression levels in week 1 compared to week 2. Description indicates gene ontology variables; MF – Molecular function, BP – Biological process, CC Cellular Component

Contrasts between harvest points	No. of DE genes	GO terms and PFAM domains	Frequency	Adjusted p-value	Description	Comment
UP unmodified – DOWN WPG4 W3	1610	**PF00096.21**	36/163 (22.09%)	4.777700e-06	PFAM	Zf-C2H2
		**PF00646.28**	25/136 (18.38%)	2.905107e-02	PFAM	F-box domain
		**PF02212.13**	5/7 (71.43%)	3.541229e-02	PFAM	Dynamin GTPase effector domain
		GO:0005515	137/1361 (10.07%)	5.967188e-04	MF	Protein binding
		GO:0006355	39/275 (14.18%)	7.109401e-03	BP	Regulation of transcription, DNA-templated
DOWN unmodified – UP WPG4 W3	544	**PF14310.1**	5/7 (71.43%)	3.282215e-04	PFAM	Fibronectin type III-like domain
		**PF07690.11**	22/222 (9.91%)	5.895353e-04	PFAM	Major Facilitator Superfamily
		**PF01055.21**	5/8 (62.50%)	8.536436e-04	PFAM	GH31
		**PF00135.23**	6/19 (31.58%)	9.484138e-03	PFAM	Carboxyesterase
		**PF01915.17**	5/13 (38.46%)	1.731718e-02	PFAM	GH3
		**PF00106.20**	18/198 (9.09%)	1.962144e-02	PFAM	Short chain dehydrogenase
		GO:0004553	27/142 (19.01%)	4.157745e-13	MF	Hydrolase activity, hydrolyzing O-glycosyl compounds
		GO:0005975	29/233 (12.45%)	2.907641e-09	BP	Carbohydrate metabolic process
		GO:0055114	66/1163 (5.67%)	1.127352e-06	BP	Oxidation-reduction process
		GO:0008152	46/664 (6.93%)	1.407060e-06	BP	Metabolic process
		GO:0016491	48/736 (6.52%)	4.306897e-06	MF	Oxidoreductase activity
		GO:0055085	29/490 (5.92%)	3.762305e-02	BP	Transmembrane transport
UP unmodified – DOWN WPG4 W6	2820	GO:0005515	252/1361 (18.52%)	7.468407e-06	MF	Protein binding
		GO:0005622	63/249 (25.30%)	2.275122e-04	CC	Intracellular
		GO:0005840	55/231 (23.81%)	9.104926e-03	CC	Ribosome
DOWN unmodified – UP WPG4 W6	728	**PF07690.11**	29/222 (13.06%)	5.467143e-07	PFAM	Major Facilitator Superfamily
		**PF00106.20**	21/198 (10.61%)	4.073485e-03	PFAM	Short chain dehydrogenase
		**PF00083.19**	16/133 (12.03%)	1.110959e-02	PFAM	Sugar transporter
		**PF14310.1**	4/7 (57.14%)	3.291028e-02	PFAM	Fibronectin type III-like domain
		GO:0016491	55/736 (7.47%)	6.382151e-07	MF	Oxidoreductase activity
		GO:0008152	50/664 (7.53%)	3.301042e-06	BP	Metabolic process
		GO:0055114	70/1163 (6.02%)	2.534849e-05	BP	Oxidation-reduction process
		GO:0055085	38/490 (7.76%)	1.391373e-04	BP	Transmembrane transport
		GO:0004553	18/142 (12.68%)	3.183569e-04	MF	Hydrolase activity, hydrolyzing O-glycosyl compounds
		GO:0022857	16/135 (11.85%)	3.452869e-03	MF	Transmembrane transporter activity
		GO:0016787	22/277 (7.94%)	4.241502e-02	MF	Hydrolase activity

UP unmodified – DOWN WPG4 W9	3707	**PF13246.1**	6/6 (100.00%)	3.068035e-02		Cation ATPase	
		GO:0005515	311/1361 (22.85%)	3.443033e-08	MF	Protein binding	
		GO:0055085	119/490 (24.29%)	3.036991e-03	BP	Transmembrane transport	
DOWN unmodified – UP WPG4 W9	656	**PF07690.11**	26/222 (11.71%)	9.680994e-07	PFAM	Major Facilitator Superfamily	
		**PF00083.19**	16/133 (12.03%)	1.101851e-03	PFAM	Sugar transporter	
		**PF05049.8**	7/32 (21.88%)	2.132557e-02	PFAM	Interferon – inducible GTPase	
		GO:0055085	36/490 (7.35%)	1.275267e-05	BP	Transmembrane transport	
		GO:0022857	16/135 (11.85%)	3.380597e-04	MF	Transmembrane transporter activity	
		GO:0055114	57/1163 (4.90%)	1.294189e-03	BP	Oxidation-reduction process	
UP unmodified – DOWN WPG24 W3	3712	**PF00096.21**	53/163 (32.52%)	4.155756e-04	PFAM	Zf-C2H2	
		**PF00646.28**	43/136 (31.62%)	1.036248e-02	PFAM	F-box domain	
		**PF00227.21**	12/21 (57.14%)	2.254947e-02	PFAM	Proteasome subunit	
		GO:0005515	308/1361 (22.63%)	2.567437e-14	MF	Protein binding	
		GO:0005839	12/23 (52.17%)	2.126102e-02	CC	Proteasome core complex	proteasome
		GO:0051603	12/23 (52.17%)	2.126102e-02	BP	Proteolysis involved in cellular protein catabolic process	
DOWN unmodified – UP WPG24 W3	1399	**PF00106.20**	34/198 (17.17%)	8.731136e-05	PFAM	Short chain dehydrogenase	
		**PF08659.5**	27/142 (19.01%)	2.165692e-04	PFAM	KR domain	
		**PF00107.21**	18/76 (23.68%)	7.601752e-04	PFAM	Zinc-binding dehydrpgenase	
		**PF00150.13**	10/25 (40.00%)	1.093827e-03	PFAM	GH5	
		**PF01055.21**	6/8 (75.00%)	1.218823e-03	PFAM	GH31	
		**PF13561.1**	21/105 (20.00%)	1.916497e-03	PFAM	Enoyl-(Acyl carrier protein) reductase	
		**PF08240.7**	15/72 (20.83%)	3.054123e-02	PFAM	Alcohol dehydrogenase GroES-like domain	
		**PF13434.1**	7/17 (41.18%)	3.423496e-02	PFAM	L-lysine 6-monooxygenase (NADPH-requiring)	
		GO:0004553	47/142 (33.10%)	2.577934e-20	MF	Hydrolase activity, hydrolyzing O-glycosyl compounds	
		GO:0016491	108/736 (14.67%)	2.903727e-16	MF	Oxidoreductase activity	
		GO:0005975	53/233 (22.75%)	7.345190e-15	BP	Carbohydrate metabolic process	
		GO:0055114	137/1163 (11.78%)	7.410880e-13	BP	Oxidation-reduction process	
		GO:0008152	85/664 (12.80%)	7.511140e-09	BP	Metabolic process	
UP unmodified – DOWN WPG24 W6	2783	**PF00096.21**	46/163 (28.22%)	4.953326e-05	PFAM	Zf-C2H2	
		**PF00646.28**	36/136 (26.47%)	6.455078e-03	PFAM	F-box domain	
		**PF13894.1**	24/81 (29.63%)	2.757299e-02	PFAM	C2H2-type zinc finger	
		**PF00096.21**	46/163 (28.22%)	4.953326e-05	PFAM	Zf-C2H2	
		GO:0005515	230/1361 (16.90%)	2.295601e-09	MF	Protein binding	
DOWN unmodified – UP WPG24W6	708	**PF07690.11**	30/222 (13.51%)	9.720199e-07	PFAM	Major Facilitator Superfamily	
		**PF01055.21**	5/8 (62.50%)	2.802654e-03	PFAM	GH31	
		**PF00083.19**	17/133 (12.78%)	8.340887e-03	PFAM	Sugar transporter	
		**PF00106.20**	21/198 (10.61%)	1.533123e-02	PFAM	Short chain dehydrogenase	
		GO:0004553	28/142 (19.72%)	2.419416e-11	MF	Hydrolase activity, hydrolyzing O-glycosyl compounds	
		GO:0055114	87/1163 (7.48%)	3.908357e-10	BP	Oxidation-reduction process	
		GO:0008152	59/664 (8.89%)	5.421983e-09	BP	Metabolic process	

		GO:0016491	62/736 (8.42%)	1.441541e-08	MF	Oxidoreductase activity
		GO:0005975	30/233 (12.88%)	2.466955e-07	BP	Carbohydrate metabolic process
		GO:0022857	17/135 (12.59%)	2.560444e-03	MF	Transmembrane transporter activity
		GO:0055085	37/490 (7.55%)	3.570715e-03	BP	Transmembrane transport
		GO:0047681	5/9 (55.56%)	3.908570e-03	MF	Aryl-alcohol dehydrogenase (NADP+) activity
		GO:0016021	52/822 (6.33%)	7.498024e-03	CC	Integral component of membrane
		GO:0019439	5/12 (41.67%)	2.255878e-02	MF	Aromatic compound catabolic process
UP unmodified – DOWN WPG24 W9	1695	**PF00096.21**	40/163 (24.54%)	1.152787e-05	PFAM	Zf-C2H2
		**PF02212.13**	6/7 (85.71%)	3.400614e-03	PFAM	Dynamin GTPase effector domain
		**PF00125.19**	11/26 (42.31%)	8.286247e-03	PFAM	Core histone H2A/H2B/H3/H4
		**PF07714.12**	45/261 (17.24%)	4.856672e-02	PFAM	Tyrosine kinase
		GO:0005515	180/1361 (13.23%)	4.460870e-08	MF	Protein binding
		GO:0006468	53/342 (15.50%)	6.432827e-03	BP	Protein phosphorylation
		GO:0004672	52/338 (15.38%)	9.648461e-03	MF	Protein kinase activity
DOWN unmodified – UP WPG24 W9	533	**PF00106.20**	22/198 (11.11%)	3.745026e-05	PFAM	Short chain dehydrogenase
		**PF08659.5**	16/142 (11.27%)	2.153148e-03	PFAM	KR domain
		**PF07690.11**	20/222 (9.01%)	4.263682e-03	PFAM	Major Facilitator Superfamily
		**PF00135.23**	6/19 (31.58%)	7.441096e-03	PFAM	Carboxyesterase
		**PF13460.1**	9/54 (16.67%)	1.433089e-02	PFAM	NAD(P)H-binding
		**PF01055.21**	4/8 (50.00%)	2.969745e-02	PFAM	GH31
		**PF13561.1**	12/105 (11.43%)	3.249352e-02	PFAM	Enoyl-(Acyl carrier protein) reductase
		**PF00107.21**	10/76 (13.16%)	4.180794e-02	PFAM	Zinc-binding dehydrogenase
		GO:0016491	58/736 (7.88%)	4.645686e-12	MF	Oxidoreductase activity
		GO:0055114	74/1163 (6.36%)	3.314468e-11	BP	Oxidation-reduction process
		GO:0008152	53/664 (7.98%)	5.266987e-11	BP	Metabolic process
		GO:0004553	16/142 (11.27%)	5.508938e-04	MF	Hydrolase activity, hydrolyzing O-glycosyl compounds
		GO:0047681	4/9 (44.44%)	3.743465e-02	MF	Aryl-alcohol dehydrogenase (NADP+) activity
		GO:0051287	9/69 (13.04%)	4.920881e-02	MF	NAD binding
UP unmodified – DOWN WPG24 W12	2574	**PF00096.21**	44/163 (26.99%)	8.347055e-05	PFAM	Zf-C2H2
		**PF02212.13**	6/7 (85.71%)	1.272597e-02	PFAM	Dynamin GTPase effector domain
		GO:0005515	199/1361 (14.62%)	2.016899e-04	MF	Protein binding
		GO:0006468	60/342 (17.54%)	3.465631e-02	BP	Protein phosphorylation
		GO:0004672	59/338 (17.46%)	4.644362e-02	MF	Protein kinase activity
DOWN unmodified – UP WPG24 W12	882	**PF07690.11**	33/222 (14.86%)	1.930592e-07	PFAM	Major Facilitator Superfamily
		**PF00107.21**	15/76 (19.74%)	4.050231e-04	PFAM	Zinc-binding dehydrpgenase
		**PF00083.19**	19/133 (14.29%)	2.380004e-03	PFAM	Sugar transporter
		**PF08240.7**	13/72 (18.06%)	6.467311e-03	PFAM	Alcohol dehydrogenase GroES-like domain
		O:0016491	70/736 (9.51%)	2.815393e-10	MF	Oxidoreductase activity

		GO:0055114	90/1163 (7.74%)	1.081507e-08	BP	Oxidation-reduction process
		GO:0008152	55/664 (8.28%)	2.091701e-05	BP	Metabolic process
		GO:0022857	19/135 (14.07%)	5.804096e-04	MF	Transmembrane transporter activity
		GO:0016787	27/277 (9.75%)	4.984093e-03	MF	Hydrolase activity
		GO:0055085	38/490 (7.76%)	1.614836e-02	BP	Transmembrane transport
UP unmodified – DOWN WPG24 W15	2948	**PF00096.21**	43/163 (26.38%)	3.643544e-03	PFAM	Zf-C2H2
		**PF02212.13**	6/7 (85.71%)	2.317659e-02	PFAM	Dynamin GTPase effector domain
		GO:0005622	54/249 (21.69%)	3.261125e-03	CC	Intracellular
		GO:0005515	209/1361 (15.36%)	6.319389e-03	MF	Protein binding
		GO:0033178	6/8 (75.00%)	4.399613e-02	CC	Proton-transporting two-sector ATPase complex, catalytic domain
DOWN unmodified – UP WPG24 W15	882	**PF07690.11**	36/222 (16.22%)	5.620654e-08	PFAM	Major Facilitator Superfamily
		**PF00083.19**	22/133 (16.54%)	2.054770e-04	PFAM	Sugar transporter
		GO:0016491	67/736 (9.10%)	1.200781e-06	MF	Oxidoreductase activity
		GO:0022857	22/135 (16.30%)	3.880504e-05	MF	Transmembrane transporter activity
		GO:0055085	47/490 (9.59%)	9.233721e-05	BP	Transmembrane transport
		GO:0055114	86/1163 (7.39%)	1.048232e-04	BP	Oxidation-reduction process
		GO:0008152	56/664 (8.43%)	4.125120e-04	BP	Metabolic process
		GO:0016787	27/277 (9.75%)	3.780954e-02	MF	Hydrolase activity
UP unmodified – DOWN WPG24 W18	2221	**PF00107.21**	14/76 (18.42%)	8.432786e-03	PFAM	Zinc-binding dehydrogenase
		**PF00096.21**	39/163 (23.93%)	1.369471e-03	PFAM	Zf-C2H2
		**PF02212.13**	6/7 (85.71%)	7.749420e-03	PFAM	Dynamin GTPase effector domain
		**PF00125.19**	11/26 (42.31%)	3.112105e-02	PFAM	Core histone H2A/H2B/H3/H4
		GO:0005840	56/231 (24.24%)	4.215594e-08	CC	Ribosome
		GO:0005622	55/249 (22.09%)	2.517659e-06	CC	Intracellular
		GO:0003735	56/281 (19.93%)	8.263977e-05	MF	Structural constituent of ribosome
		GO:0006412	53/270 (19.63%)	3.006881e-04	BP	Translation
DOWN unmodified – UP WPG24 W18	801	**PF07690.11**	37/222 (16.67%)	8.973996e-10	PFAM	Major Facilitator Superfamily
		**PF00083.19**	20/133 (15.04%)	8.325642e-04	PFAM	Sugar transporter
		**PF00106.20**	25/198 (12.63%)	1.141259e-03	PFAM	Short chain dehydrogenase
		**PF13561.1**	16/105 (15.24%)	8.209142e-03	PFAM	Enoyl-(Acyl carrier protein) reductase
		GO:0016491	71/736 (9.65%)	2.992889e-10	MF	Oxidoreductase activity
		GO:0055114	88/1163 (7.57%)	2.352223e-07	BP	Oxidation-reduction process
		GO:0008152	60/664 (9.04%)	3.799051e-07	BP	Metabolic process
		GO:0022857	20/135 (14.81%)	1.834058e-04	MF	Transmembrane transporter activity
		GO:0055085	42/490 (8.57%)	8.160709e-04	BP	Transmembrane transport

UP unmodified – DOWN WPG37 W3	1044	**PF00096.21**	25/163 (15.34%)	4.859654e-03	PFAM	Zf-C2H2
		**PF14531.1**	9/28 (32.14%)	1.224654e-02	PFAM	Kinase-like
		**PF00646.28**	21/136 (15.44%)	2.358085e-02	PFAM	F-box domain
		**PF00627.26**	5/9 (55.56%)	4.574065e-02	PFAM	UBA (ubiquitin-associated) protein domain
		GO:0005515	130/1361 (9.55%)	6.093688e-11	MF	Protein binding
DOWN unmodified – UP WPG37 W3	495	**PF00106.20**	25/198 (12.63%)	1.502376e-07	PFAM	Short chain dehydrogenase
		**PF08659.5**	21/142 (14.79%)	2.350456e-07	PFAM	KR domain
		**PF13561.1**	17/105 (16.19%)	2.916457e-06	PFAM	Enoyl-(Acyl carrier protein) reductase
		**PF00067.17**	27/309 (8.74%)	9.091704e-05	PFAM	Cytochrome P450
		**PF00107.21**	13/76 (17.11%)	1.042658e-04	PFAM	Zinc-binding dehydrogenase
		**PF05368.8**	7/20 (35.00%)	4.820377e-04	PFAM	NmrA-like family
		**PF07690.11**	21/222 (9.46%)	6.728202e-04	PFAM	Major Facilitator Superfamily
		**PF00171.17**	9/41 (21.95%)	1.005710e-03	PFAM	Aldehyde dehydrogenase family
		**PF13460.1**	10/54 (18.52%)	1.374849e-03	PFAM	NAD(P)H-binding
		**PF00724.15**	6/24 (25.00%)	2.730157e-02	PFAM	NADH: flavin oxidoreductase /NADH oxidase family
		**PF01370.16**	11/94 (11.70%)	4.047907e-02	PFAM	NADH dehydrogenase (ubiquinone)
		GO:0016491	75/736 (10.19%)	1.286305e-24	MF	Oxidoreductase activity
		GO:0055114	94/1163 (8.08%)	2.373710e-24	BP	Oxidation-reduction process
		GO:0008152	49/664 (7.38%)	2.620519e-09	BP	Metabolic process
		GO:0005506	30/367 (8.17%)	6.471919e-06	MF	Iron ion binding
		GO:0016705	27/326 (8.28%)	2.899454e-05	MF	Oxidoreductase activity, acting on paired donors, with incorporation or reduction of molecular oxygen
		GO:0020037	31/419 (7.40%)	3.701968e-05	MF	Heme binding
		GO:0050662	11/101 (10.89%)	3.078028e-02	MF	Coenzyme binding
UP unmodified – DOWN WPG37 W6	2403	**PF00227.21**	13/21 (61.90%)	1.423343e-04	PFAM	Proteasome subunit
		**PF10584.4**	7/8 (87.50%)	2.964410e-03	PFAM	Proteasome_A_N
		**PF00125.19**	13/26 (50.00%)	3.866294e-03	PFAM	Core histone H2A/H2B/H3/H4
		GO:0005515	238/1361 (17.49%)	2.434218e-10	MF	Protein binding
		GO:0005839	13/23 (56.52%)	1.454295e-04	CC	Proteasome core complex
		GO:0051603	13/23 (56.52%)	1.454295e-04	BP	Proteolysis involved in cellular protein catabolic process
		GO:0004298	13/28 (46.43%)	2.750044e-03	MF	Threonine-type endopeptidase activity
		GO:0019773	7/10 (70.00%)	1.617852e-02	CC	Proteasome core complex, alpha-subunit complex
		GO:0004672	63/338 (18.64%)	3.418649e-02	MF	Protein kinase activity
DOWN unmodified – UP WPG37 W6	653	**PF07690.11**	30/222 (13.51%)	8.533673e-08	PFAM	Major Facilitator Superfamily
		**PF00106.20**	25/198 (12.63%)	1.313163e-05	PFAM	Short chain dehydrogenase
		**PF00107.21**	15/76 (19.74%)	2.645054e-05	PFAM	Zinc-binding dehydrogenase
		**PF08659.5**	20/142 (14.08%)	5.862712e-05	PFAM	KR domain
		**PF13561.1**	16/105 (15.24%)	3.987460e-04	PFAM	Enoyl-(Acyl carrier protein) reductase
		**PF00067.17**	27/309 (8.74%)	6.123613e-03	PFAM	Cytochrome P450
		**PF00083.19**	16/133 (12.03%)	9.444995e-03	PFAM	Sugar transporter

		**PF08240.7**	11/72 (15.28%)	2.315062e-02	PFAM	Alcohol dehydrogenase GroES-like domain
		GO:0016491	74/736 (10.05%)	2.176798e-17	MF	Oxidoreductase activity
		GO:0055114	92/1163 (7.91%)	2.819332e-15	BP	Oxidation-reduction process
		GO:0008152	51/664 (7.68%)	1.015270e-06	BP	Metabolic process
		GO:0055085	38/490 (7.76%)	1.280558e-04	BP	Transmembrane transport
		GO:0005506	31/367 (8.45%)	3.052299e-04	MF	Iron ion binding
		GO:0019439	6/12 (50.00%)	5.237034e-04	BP	Aromatic compound catabolic process
		GO:0020037	32/419 (7.64%)	1.809850e-03	MF	Heme binding
		GO:0047681	5/9 (55.56%)	2.459728e-03	MF	Aryl-alcohol dehydrogenase (NADP+) activity
		GO:0016705	27/326 (8.28%)	2.583631e-03	MF	oxidoreductase activity, acting on paired donors, with incorporation or reduction of molecular oxygen
		GO:0022857	16/135 (11.85%)	3.314913e-03	MF	Transmembrane transporter activity
UP unmodified – DOWN WPG37 W9	2266	**PF00227.21**	13/21 (61.90%)	7.270874e-05	PFAM	Proteasome subunit
		**PF00125.19**	14/26 (53.85%)	2.587435e-04	PFAM	Core histone H2A/H2B/H3/H4
		**PF10584.4**	7/8 (87.50%)	2.016023e-03	PFAM	Proteasome_A_N
		**PF02212.13**	6/7 (85.71%)	1.445209e-02	PFAM	Dynamin GTPase effector domain
		**PF00096.21**	38/163 (23.31%)	3.997227e-02	PFAM	Zf-C2H2
		**PF01399.22**	9/17 (52.94%)	4.054754e-02	PFAM	PCI domain
		GO:0005515	248/1361 (18.22%)	2.108436e-16	MF	Protein binding
		GO:0005839	13/23 (56.52%)	7.407123e-05	CC	Proteasome core complex
		GO:0051603	13/23 (56.52%)	7.407123e-05	BP	Proteolysis involved in cellular protein catabolic process
		GO:0004298	13/28 (46.43%)	1.446003e-03	MF	Threonine-type endopeptidase activity
		GO:0019773	7/10 (70.00%)	1.107768e-02	CC	Proteasome core complex, alpha-subunit complex
		GO:0004175	7/11 (63.64%)	2.762722e-02	MF	Endopeptidase activity
DOWN unmodified – UP WPG37 W9	617	**PF07690.11**	28/222 (12.61%)	6.736319e-07	PFAM	Major Facilitator Superfamily
		**PF00106.20**	26/198 (13.13%)	1.150830e-06	PFAM	Short chain dehydrogenase
		**PF08659.5**	20/142 (14.08%)	2.845723e-05	PFAM	KR domain
		**PF13561.1**	17/105 (16.19%)	3.764197e-05	PFAM	Enoyl-(Acyl carrier protein) reductase
		**PF00107.21**	14/76 (18.42%)	1.095751e-04	PFAM	Zinc-binding dehydrogenase
		**PF00067.17**	28/309 (9.06%)	8.673887e-04	PFAM	Cytochrome P450
		**PF00083.19**	17/133 (12.78%)	1.217861e-03	PFAM	Sugar transporter
		**PF01055.21**	5/8 (62.50%)	1.380587e-03	PFAM	GH31
		**PF13460.1**	9/54 (16.67%)	4.284313e-02	PFAM	NAD(P)H-binding
		GO:0016491	72/736 (9.78%)	1.959615e-17	MF	Oxidoreductase activity
		GO:0055114	90/1163 (7.74%)	9.594555e-16	BP	Oxidation-reduction process
		GO:0008152	53/664 (7.98%)	1.661888e-08	BP	Metabolic process
		GO:0005506	33/367 (8.99%)	8.492336e-06	MF	Iron ion binding
		GO:0016705	28/326 (8.59%)	3.044840e-04	MF	Oxidoreductase activity, acting on paired donors, with incorporation or reduction of molecular oxygen
		GO:0022857	17/135 (12.59%)	3.554264e-04	MF	Transmembrane transporter activity

		GO:0019439	6/12 (50.00%)	3.925440e-04	BP	Aromatic compound catabolic process
		GO:0020037	32/419 (7.64%)	6.189797e-04	MF	Heme binding
		GO:0047681	5/9 (55.56%)	1.932421e-03	MF	Aryl-alcohol dehydrogenase (NADP+) activity
		GO:0055085	34/490 (6.94%)	2.424179e-03	BP	Transmembrane transport
		GO:0004553	15/142 (10.56%)	1.499954e-02	MF	Hydrolase activity, hydrolyzing O-glycosyl compounds
UP unmodified – DOWN WPG37 W12	1873	**PF00646.28**	34/136 (25.00%)	2.706560e-04	PFAM	F-box domain
		**PF00125.19**	12/26 (46.15%)	1.796382e-03	PFAM	Core histone H2A/H2B/H3/H4
		**PF00096.21**	34/163 (20.86%)	1.959258e-02	PFAM	Zf-C2H2
		GO:0005515	200/1361 (14.70%)	1.264286e-11	MF	Protein binding
DOWN unmodified – UP WPG37 W12	610	**PF07690.11**	34/222 (15.32%)	3.589247e-10	PFAM	Major Facilitator Superfamily
		**PF00083.19**	21/133 (15.79%)	6.857682e-06	PFAM	Sugar transporter
		**PF00106.20**	24/198 (12.12%)	1.166655e-04	PFAM	Short chain dehydrogenase
		**PF08659.5**	19/142 (13.38%)	5.094792e-04	PFAM	KR domain
		**PF13561.1**	16/105 (15.24%)	6.674237e-04	PFAM	Enoyl-(Acyl carrier protein) reductase
		**PF00107.21**	13/76 (17.11%)	1.866623e-03	PFAM	Zinc-binding dehydrogenase
		**PF00067.17**	28/309 (9.06%)	4.330735e-03	PFAM	Cytochrome P450
		**PF08240.7**	11/72 (15.28%)	3.296372e-02	PFAM	Alcohol dehydrogenase GroES-like domain
		GO:0016491	74/736 (10.05%)	1.782601e-17	MF	Oxidoreductase activity
		GO:0055114	93/1163 (8.00%)	6.503982e-16	BP	Oxidation-reduction process
		GO:0008152	61/664 (9.19%)	5.438036e-12	BP	Metabolic process
		GO:0022857	18/135 (13.33%)	1.336872e-04	MF	Transmembrane transporter activity
		GO:0005506	31/367 (8.45%)	2.844494e-04	MF	Iron ion binding
		GO:0055085	37/490 (7.55%)	3.386116e-04	BP	Transmembrane transport
		GO:0020037	32/419 (7.64%)	1.689337e-03	MF	Heme binding
		GO:0047681	5/9 (55.56%)	2.421381e-03	MF	Aryl-alcohol dehydrogenase (NADP+) activity
		GO:0016705	27/326 (8.28%)	2.432141e-03	MF	Oxidoreductase activity, acting on paired donors, with incorporation or reduction of molecular oxygen
		GO:0019439	5/12 (41.67%)	1.408900e-02	BP	Aromatic compound catabolic process
		GO:0016021	46/822 (5.60%)	4.605929e-02	CC	Integral component of membrane
UP unmodified – DOWN WPG37 W15	1681	**PF00096.21**	35/163 (21.47%)	2.132706e-04	PFAM	Zf-C2H2
		**PF00227.21**	10/21 (47.62%)	2.170399e-03	PFAM	Proteasome subunit
		**PF00125.19**	11/26 (42.31%)	2.917628e-03	PFAM	Core histone H2A/H2B/H3/H4
		**PF10584.4**	6/8 (75.00%)	6.660838e-03	PFAM	Proteasome_A_N
		**PF13894.1**	19/81 (23.46%)	2.999535e-02	PFAM	C2H2-type zinc finger
		**PF14531.1**	10/28 (35.71%)	4.611037e-02	PFAM	Kinase-like
		GO:0005515	166/1361 (12.20%)	4.525796e-08	MF	Protein binding
		GO:0005839	10/23 (43.48%)	2.077529e-03	CC	Proteasome core complex

		GO:0051603	10/23 (43.48%)	2.077529e-03	BP	Proteolysis involved in cellular protein catabolic process
		GO:0004298	10/28 (35.71%)	1.679805e-02	MF	Threonine-type endopeptidase activity
		GO:0019773	6/10 (60.00%)	2.282638e-02	CC	Proteasome core complex, alpha-subunit complex
		GO:0004175	6/11 (54.55%)	4.699426e-02	MF	Endopeptidase activity
DOWN unmodified – UP WPG37 W15	643	**PF07690.11**	34/222 (15.32%)	3.589247e-10	PFAM	Major Facilitator Superfamily
		**PF00083.19**	21/133 (15.79%)	6.857682e-06	PFAM	Sugar transporter
		**PF00106.20**	24/198 (12.12%)	1.166655e-04	PFAM	Short chain dehydrogenase
		**PF08659.5**	19/142 (13.38%)	5.094792e-04	PFAM	KR domain
		**PF13561.1**	16/105 (15.24%)	6.674237e-04	PFAM	Enoyl-(Acyl carrier protein) reductase
		**PF00107.21**	13/76 (17.11%)	1.866623e-03	PFAM	Zinc-binding dehydrogenase
		**PF00067.17**	28/309 (9.06%)	4.330735e-03	PFAM	Cytochrome P450
		**PF08240.7**	11/72 (15.28%)	3.296372e-02	PFAM	Alcohol dehydrogenase GroES-like domain
		GO:0016491	72/736 (9.78%)	2.056484e-15	MF	Oxidoreductase activity
		GO:0055114	91/1163 (7.82%)	6.958420e-14	BP	Oxidation-reduction process
		GO:0022857	21/135 (15.56%)	1.264243e-06	MF	Transmembrane transporter activity
		GO:0008152	51/664 (7.68%)	2.763521e-06	BP	Metabolic process
		GO:0055085	41/490 (8.37%)	1.040032e-05	BP	Transmembrane transport
		GO:0005506	33/367 (8.99%)	5.612170e-05	MF	Iron ion binding
		GO:0020037	33/419 (7.88%)	1.184699e-03	MF	Heme binding
		GO:0016705	28/326 (8.59%)	1.441167e-03	MF	Oxidoreductase activity, acting on paired donors, with incorporation or reduction of molecular oxygen
UP unmodified – DOWN WPG37 W18	1681	**PF00125.19**	11/26 (42.31%)	2.629458e-03	PFAM	Core histone H2A/H2B/H3/H4
		**PF10584.4**	6/8 (75.00%)	6.259235e-03	PFAM	Proteasome_A_N
		**PF14531.1**	10/28 (35.71%)	4.210987e-02	PFAM	Kinase-like
		**PF00646.28**	26/136 (19.12%)	4.876051e-02	PFAM	F-box domain
		GO:0005515	162/1361 (11.90%)	1.769267e-07	MF	Protein binding
		GO:0019773	6/10 (60.00%)	2.100160e-02	CC	Proteasome core complex, alpha-subunit complex
		GO:0004175	6/11 (54.55%)	4.328024e-02	MF	Endopeptidase activity
DOWN unmodified – UP WPG37 W18	610	**PF07690.11**	32/222 (14.41%)	5.320101e-10	PFAM	Major Facilitator Superfamily
		**PF00067.17**	30/309 (9.71%)	4.553502e-05	PFAM	Cytochrome P450
		**PF00083.19**	18/133 (13.53%)	1.770193e-04	PFAM	Sugar transporter
		**PF00106.20**	22/198 (11.11%)	2.839922e-04	PFAM	Short chain dehydrogenase
		**PF00107.21**	13/76 (17.11%)	5.661292e-04	PFAM	Zinc-binding dehydrogenase
		**PF08659.5**	17/142 (11.97%)	2.185837e-03	PFAM	KR domain
		**PF13561.1**	14/105 (13.33%)	4.643872e-03	PFAM	Enoyl-(Acyl carrier protein) reductase
		**PF01055.21**	4/8 (50.00%)	4.665164e-02	PFAM	GH31
		GO:0055114	89/1163 (7.65%)	4.762138e-16	BP	Oxidation-reduction process

GO:0016491	68/736 (9.24%)	1.338700e-15	MF	Oxidoreductase activity
GO:0005506	35/367 (9.54%)	2.914483e-07	MF	Iron ion binding
GO:0055085	39/490 (7.96%)	5.182263e-06	BP	Transmembrane transport
GO:0008152	47/664 (7.08%)	5.553846e-06	BP	Metabolic process
GO:0016705	30/326 (9.20%)	1.315417e-05	MF	Oxidoreductase activity, acting on paired donors, with incorporation or reduction of molecular oxygen
GO:0020037	34/419 (8.11%)	3.291736e-05	MF	Heme binding
GO:0022857	18/135 (13.33%)	4.439663e-05	MF	Transmembrane transporter activity
GO:0019439	6/12 (50.00%)	3.345246e-04	BP	Aromatic compound catabolic process
GO:0047681	5/9 (55.56%)	1.690378e-03	MF	Aryl-alcohol dehydrogenase (NADP+) activity

Supplementary table 9. qRT-PCR primers used for qRT-PCR analyses of selected genes with function related to plant cell wall decay. Primers are designed based on JGI protein id for *Rhodonia* (*Postia*) *placenta* genome MAD-698-R.

Gene (abbreviation)	JGI protein id	Function	Forward primer/reverse primer
Housekeeping genes			
β-tubulin (βt)	113871	Major component of the eukaryotic cytoskeleton.	CAGGATCTTGTCGCCGAGTAC/ CCTCATACTCGCCCTCCTCTT
α-tubulin (αt)	123093	Major component of the eukaryotic cytoskeleton.	GGAGTCGCCTTGACCACAA/ TGCCCTCACCAACGTACCA
**1: Genes involved in oxidative depolymerisation**			
1.1: Oxalate synthesis and oxalate decomposition			
Glyoxylate dehydrogenase (GlyD)	121561	Involved in oxalate synthesis	CGGAGCTGGACCTTTGTTAC/ GCGCGAAGGCAAATCTAATA
Oxaloacetate acetylhydrolase (OahA)	112832	Involved in oxalate synthesis	AAGGCGTTCTTCGAGGTCAT/ AAAGCAGCAACCCGAGAAG
Oxalate decarboxylase (OxaD)	43912	Involved in oxalate decomposition	GAACCTATAACTACGAGGCAAGC/ CCAGGAATACCAGAGGCTCA
1.2: Redox enzymes			
AA3 GMC oxidoreductase (AOx1)	44331	Involved in oxidative depolymerization. Likely source of H_2O_2.	GGAGGTACAGACGGACGAAC/ AGAGTCGACGACACCGTTCT
AA3 GMC oxidoreductases (AOx2)	129158	Involved in oxidative depolymerization. Likely source of H_2O_2.	TACTCGACGGCCCTCACTAT/ CCGCTTGAGACTGAACACTG
AA3 GMC oxidoreductase (AOx3)	118723	Involved in oxidative depolymerization. Likely source of H_2O_2.	ACACCAAGGAGGACGACGAG/ GACGAGCAAGGCAGACGAGTA
AA3_3 Alcohol oxidase () (AOx4)	55972	Involved in oxidative depolymerization. Likely source of H_2O_2.	CCCATTAAGCTGAGCGATCC/ TCGTCGCCACAACCTCTTTA
AA5 Copper radical oxidase (Cro1)	56703	Involved in oxidative depolymerization. Likely source of H_2O_2.	CGGCGATGTTTCGGACGTTAT/ CCGCCATTCCAATAGTAGAGC
AA5 Copper radical oxidase (Cro2)	104114	Involved in oxidative depolymerization. Likely source of H_2O_2.	CGCAGACGATGGAGGTGGTC/ GTGACACCGCACCGTTACCA
AA6 Benzoquinone reductases (BqR)	124517	Involved in oxidative depolymerization. Possibly involved in reduction/regeneration of chelator/reductants	CGTACAAAGAACGCCCTCTC/ GTGGCCGTACATGGAGTAGA
**2: Hydrolytic enzymes involved in polysaccharide depolymerisation and LPMO**			
1.1: Cellulose degradation			
GH5 Endoglucanase (Cel5a)	115648	Major endocellulase	TTCTGTCCATGACACCGTACA/ TCCTCTTGGTGTAGGTCCGTA
GH5 Endoglucanase (Cel5b)	103675	Major endocellulase	CTCGCATACGTGCAATCG/ GGAGTAGGGCGTCACAGAGA
GH12 Glucoside hydrolase (XyGEg)	121191	Endoglucanase active on cellulose or xyloglucan	TCAACGTCGAGAGCTTCAG/ GACGAAGAGCTAAGGACACCA
AA9 Lytic polysaccharide monooxygenases (LPMO)	126811	Polysaccharide depolymerization via oxidative cleavage of glycosidic bonds.	GCCAGATATCACGGTCACCT/ TCGTAGATGTCGGGAACGTA
GH3 Betaglucosidase (bGlu)	128500	Hydrolyses cellobiose, releasing glucose	AGGCACAAGCCAAGTCGTCA/ CTTGGCAATCGTGAAAGTGGT
2.1: Hemicellulose and pectin degradation			
Endomannanase (Man5a)	121831	Involved in glucomannan depolymerization, highly expressed	GCTGACTGGCACCGACTACC/ CCCACGAACGCATCCAAATAG

GH10 Endoxylanase (Xyl10a)	113670	Involved in xylose depolymerization	CTTCGGCTCTGCTACGGACAA/ ACCATACGCAGTTGTGTCCTCT
GH10b Endoxylanase (Xyl10b)	105534	Involved in xylose depolymerization	TCGGAGCCTGAGCCATTTGT/ TGCTGCGGTGTAATTGTTGG
GH3 Beta xylosidase (bXyl)	51213	Hemicellulose depolymerization	GTGCGTTTCCCGACTGTGC/ GCGGTGTTGCCGGTATTGT
CE16 Carbohydrate esterase (CE16a)	125801	Deacetylation of polysaccharides	ACACCGTGCACAACATCCT/ CGTGCTCCAAGTCTGATGAT
CE 16 Carbohydrate esterase family 16 (CE16b)	48548	Deacetylation of polysaccharides	CTCTGGTGGGACGAGGTG/ CAAATCAGCCACGTTATCCA
GH28 Polygalacturonase (Gal28a)	111730	Involved pectin depolymerization	CCGGCAATACAATTTCTGGCA/ GTTCCGGGAGTACCGTCATT
**3: Expansins**			
Expansin (Exp1)	126976	Most likely involved in increasing enzyme accessibility	TGTCGGAATGAGCGGTCT/ ATGCATGAACCGCCTTTGT
Expansin (Exp2)	128179	Most likely involved in increasing enzyme accessibility	AATGTGACTTGGGCCATTGT/ AATACCGTGCAAGCGTCAGT
